# Comparative efficacy of lipid-lowering therapies on the cardio-renal-metabolic axis in diabetic kidney disease: a Bayesian network meta-analysis addressing residual CRM risk

**DOI:** 10.3389/fendo.2026.1842847

**Published:** 2026-05-29

**Authors:** Jun Luo, Yinzhong Dai, Chenguang Wu, Chengying Lan, Junwei Shi, Jinfeng Qi, Shimei Hua, Yan An, Lifan Wang, Ping Li, Peng Liu

**Affiliations:** 1 Renal Division, Department of Medicine, Heilongjiang Academy of Chinese Medicine Sciences, Harbin, China; 2 Xiyuan Hospital, China Academy of Chinese Medical Sciences, Beijing, China; 3 Beijing Key Lab for Immune-Mediated Inflammatory Diseases, China-Japan Friendship Hospital, Beijing, China

**Keywords:** Bayesian network meta-analysis, cardio-renal-metabolic, diabetic kidney disease, lipid-lowering therapies, residual cardiovascular risk

## Abstract

**Background:**

In diabetic kidney disease (DKD), dyslipidemia accelerates renal decline and heart failure risk, making Cardio-Renal-Metabolic (CRM) risk reduction critical. The bidirectional relationship between cardiac and renal dysfunction further highlights the necessity of lipid-lowering therapy as a common intervention pathway; however, its comparative efficacy remains unclear.

**Objective:**

To compare the Cardio-Renal-Metabolic (CRM) outcomes of various lipid-lowering agents.

**Methods:**

We searched PubMed, Embase, Web of Science, and Cochrane Library for Randomized Controlled Trials (RCTs) up to May 10, 2025; a Bayesian network meta-analysis compared their effects.

**Results:**

From 20 RCTs, Cerivastatin best reduced Total Cholesterol (TC) (Mean Difference (MD): -94.03, 95% CI: -185.37 to -2.16), while Simvastatin best lowered Low-Density Lipoprotein Cholesterol (LDL-C) (MD: -56.05, 95% CI: -101.64 to -11.66). For cardiorenal outcomes, Atorvastatin, Rosuvastatin, and Fenofibrate potentially improved Urine Albumin-to-Creatinine Ratio (UACR). Atorvastatin (MD: -3.19, 95% CI: -5.12 to -1.27) and Fenofibrate (MD: -1.44, 95% CI: -2.78 to -0.09) most robustly reduced cardiovascular event rates (CVER) in hyperlipidemic DKD.

**Conclusion:**

Lipid-lowering agents have divergent effects; Atorvastatin and Fenofibrate best reduce CVER despite variable renal effects, supporting a phenotype-driven CRM strategy prioritizing residual cardiovascular risk reduction.

**Systematic review registration:**

https://www.crd.york.ac.uk/PROSPERO/view/, identifier CRD420251049719.

## 1 Introduction

Type 2 Diabetes Mellitus (T2DM) and its major and most severe microvascular complication, diabetic kidney disease (DKD) (1), collectively constitute one of the most pressing global public health challenges of the 21st century (2, 3). According to relevant statistics, the number of adults globally living with diabetes has quadrupled, soaring from 108 million in 1980 to 463 million in 2019, and is projected to reach 700 million by 2045 (4). Furthermore, an estimated 25% to 40% of individuals with diabetes will subsequently develop DKD (5). DKD is not only the leading etiology of End-Stage Kidney Disease (ESKD) (6, 7), but also a potent cardiovascular (CV) risk multiplier (8, 9). Beyond its role in amplifying cardiovascular risk, DKD exemplifies a complex bidirectional dysfunction: hemodynamic instability resulting from cardiac impairment, such as reduced arterial perfusion and venous congestion, can act as a secondary driver of renal structural damage, thereby creating a self-perpetuating cardiorenal vicious cycle. Patients with DKD have significantly elevated rates of cardiovascular events and mortality compared to those without DKD (10). The mortality risk in this population is predominantly driven by cardiovascular complications, rather than renal failure per se. This dire reality highlights the imperative need to place renal protection and cardiovascular risk control on an equally important footing in the management of DKD (11). Cardio-Renal-Metabolic (CRM) axis refers to the pathophysiological interconnection where systemic metabolic derangements, particularly dyslipidemia, drive reciprocal cardiac and renal dysfunction. This framework views DKD not in isolation, but as a core component of integrated cardiorenal failure.

Based on a large body of evidence confirming that statins significantly reduce the risk of Atherosclerotic Cardiovascular Disease (ASCVD), major global authoritative guidelines, including the Kidney Disease: Improving Global Outcomes (KDIGO) 2024 Guidelines and the American Diabetes Association (ADA) 2024 Guidelines, explicitly recommend the use of statins for both primary and secondary prevention in the majority of adult DKD patients (12, 13). This recommendation has thus become a cornerstone of cardiovascular risk management for patients with DKD (14). Moreover, while the American Heart Association (AHA) guidelines reaffirm statins as the cornerstone of therapy, they also recommend PCSK9 inhibitors, ezetimibe, and bile acid sequestrants for secondary prevention of ASCVD (15). Furthermore, the current AHA and ESC (European Society of Cardiology) guidelines emphasize risk-based lipid targets, particularly for patients at high or very high cardiovascular risk, such as those with DKD, to guide clinical intensity of therapy beyond simple percentage reductions (16). Meanwhile, KDIGO suggests exploring potential benefits of fibrates and niacin in patients with Chronic Kidney Disease (CKD) (12). However, these recommendations are not entirely based on RCTs evidence specifically in the DKD population. Accordingly, the objective of this study is to systematically evaluate and compare the relative efficacy of different lipid-lowering agents on cardiorenal outcomes in the DKD population based on existing RCT evidence.

The heart, kidney, and metabolic systems exhibit profound pathological synergy within the CRM framework (17). Although current intensive management strategies have alleviated systemic stress to some extent, lipid-mediated residual risk remains a primary driver of progressive renal decline and cardiovascular events. In this context, prioritizing precise lipid management as an independent core of CRM protection is essential to disrupt the vicious cycle of cardio-renal damage.

## 2 Methods

This study was conducted in accordance with the PRISMA-NMA reporting guidelines, and the prospective protocol was registered (PROSPERO-ID: CRD420251049719).

### 2.1 Search strategy

We systematically searched electronic databases (PubMed, Embase, Cochrane Library, and Web of Science) from their inception up to May 10, 2025. We also examined prior reviews and conference proceedings, with no language restrictions. Following the search, RCTs were included according to our pre-specified criteria. This systematic search was supplemented by various methods, including but not limited to, hand-searching relevant conference reports, dissertations, and theses. Furthermore, we actively contacted experts in the field, as well as corresponding authors, to obtain reports and materials that were inaccessible through standard database searches. We used a combination of free-text words and Medical Subject Headings (MeSH terms), linked by Boolean operators, to construct the core search strategy: (Hypolipidemic Agents or Hydroxymethylglutaryl CoA Reductase Inhibitors or Fibric Acids or Ezetimibe) and (Diabetic Nephropathies). While maintaining conceptual consistency, this core search strategy was tailored to meet the specific query syntax and requirements of each individual database. The full search strategy is available in the Attachment.

### 2.2 Inclusion and exclusion criteria

RCTs were included if they satisfied the following conditions: 1. Study Duration and Publication: The study duration was ≥12 weeks. There were no restrictions on blinding, allocation concealment implementation, or language of publication. 2. Study Population: Participants were patients with DKD. DKD was clinically diagnosed in diabetic patients who presented with persistent albuminuria (Urine Albumin-to-Creatinine Ratio (UACR) ≥30 mg/g) and/or a sustained estimated Glomerular Filtration Rate (eGFR<60 ml/min/1.73m^2^), after the exclusion of other renal diseases (18). 3. Intervention: The intervention group received any approved, commercially available lipid-lowering monotherapy or combination therapy. This included, but was not limited to, statins, fibrates, cholesterol absorption inhibitors (ezetimibe), and other lipid-lowering agents (probucol), with no restriction on dosage. 4. Comparison: The intervention was compared with placebo, standard care, or another lipid-lowering agent that met the inclusion criteria. 5. Outcomes: Studies were required to report a minimum of one outcome from the list below: Lipid Outcomes: Total Cholesterol (TC), Triglycerides (TG), Low-Density Lipoprotein Cholesterol (LDL-C), High-Density Lipoprotein Cholesterol (HDL-C); Renal Outcomes: eGFR, 24-hour urine total protein quantity (24hUTP), UACR, and Serum Creatinine (Scr); Cardiovascular Outcomes: Cardiovascular Event Rate (CVER); Safety Outcomes: Mortality Rate; Other Outcomes: HbA1c, etc. Exclusion Criteria: 1. Cross-over studies were excluded. 2. Other relevant literature where the intervention did not involve lipid-lowering therapy. 3. Reviews, meta-analyses, animal experiments, conference abstracts, non-clinical therapeutic studies, and similar literature types. 4. Literature for which the full text could not be obtained. 5. Duplicate publications.

### 2.3 Study selection and data extraction

Two reviewers (J.L. and Y.D.) independently screened the identified literature according to the pre-specified inclusion and exclusion criteria. The screening process was conducted sequentially: first, by reviewing the titles and abstracts for initial screening, followed by a full-text review for final eligibility assessment. We resolved any disagreements about study inclusion through discussion and consensus with a third reviewer (C.W.). Moreover, we examined the reference lists of existing meta-analyses and systematic reviews to locate additional eligible studies potentially overlooked by the primary search. For all studies that met the inclusion criteria, two independent reviewers (J.S. and J.Q.) extracted the data. The extracted information encompassed the following details: Study Characteristics: First author, publication year, country, and study design. Participant Baseline Information: Sample size, gender, duration of diabetes, baseline renal function (eGFR, Scr) and lipid levels (TC, LDL-C). Any discrepancies in the extracted data were resolved by mediation and discussion with a third independent reviewer (C.L.).

### 2.4 Quality assessment

In line with the Cochrane Handbook for Systematic Reviews of Interventions (version 5.1), we employed the Risk of Bias 2 (RoB 2) tool to assess the quality of the included RCTs. The RoB 2 tool appraises bias risk within five domains: the randomization process, deviations from intended interventions, missing outcome data, outcome measurement, and selection of reported results. Each domain was assigned a risk level of High, Moderate, or Low.

### 2.5 Statistical analysis

We employed Revman 5.4 software to perform the bias risk evaluation. The NMA was conducted using the gemtc package within RStudio. Statistical heterogeneity across the included studies was assessed using the I^2^ statistic. A fixed-effects model was employed if I^2^ < 50%, whereas a random-effects model was used if I^2^ > 50% to account for significant heterogeneity. All interventions were analyzed using a Markov Chain Monte Carlo (MCMC) model within the Bayesian framework. We ran four Markov chains, each with 50,000 iterations, discarding the first 10,000 iterations as the burn-in period. Model convergence was assessed using the Brooks-Gelman-Rubin statistic (R-hat value), where values close to 1 indicated good convergence. The Network Plot was constructed for each outcome to visually represent the geometric structure of the evidence network formed by all included studies. To evaluate the relative effectiveness and ranking of various interventions for each outcome, we calculated the probability that each intervention would rank first to last. This was visualized using The Ranking Probability plot. Furthermore, we calculated the Surface Under the Cumulative Ranking (SUCRA) curve values. The SUCRA value ranges from 0 to 1, with a higher value indicating a superior efficacy of the intervention. Finally, potential publication bias was evaluated using funnel plots and quantified via Egger’s regression test. We conducted a pooled analysis of outcome data reported at 12 months of treatment. Sensitivity analysis was performed using the leave-one-out method. For dichotomous outcomes, the Odds Ratio (OR) was used as the measure of effect size. For continuous variables, we used the Mean Difference (MD) or the Standardized Mean Difference (SMD). All effect sizes were reported with their associated 95% Confidence Interval (CI). The certainty of evidence for the primary outcomes was evaluated using the Grading of Recommendations Assessment, Development and Evaluation (GRADE) approach. We assessed the evidence network across five principal domains, including risk of bias, inconsistency, indirectness, imprecision, and publication bias. Based on these comprehensive evaluations, the overall certainty of evidence for each specific outcome was categorized into one of four distinct levels, specifically high, moderate, low, or very low. This systematic assessment provides a transparent framework for interpreting the robustness of the network meta-analysis findings.

## 3 Results

### 3.1 Study selection

**Figure 1:**
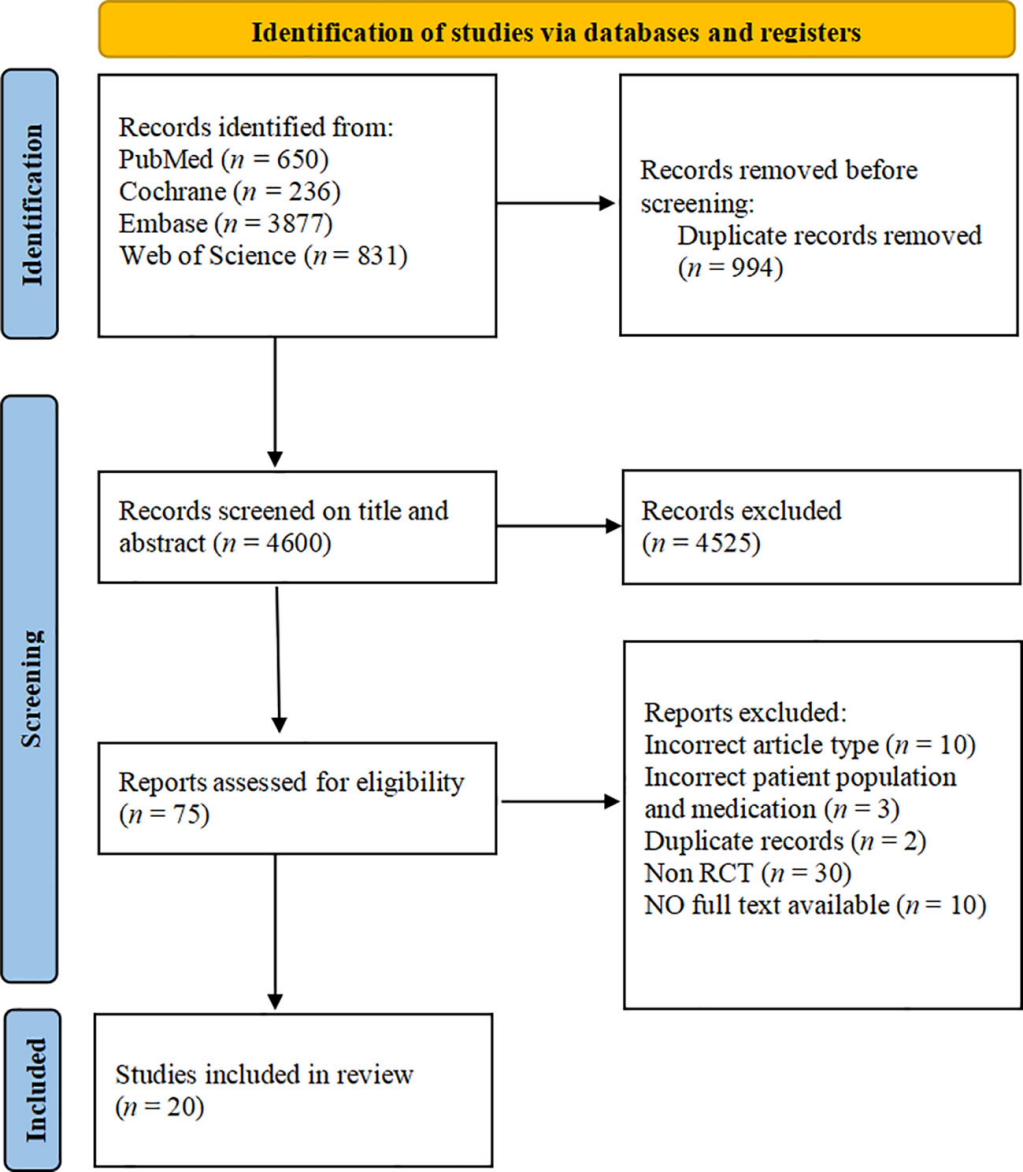
PRISMA flow diagram of study selection.

Our initial systematic search across the relevant databases identified 5,594 records. After importing these records into Endnote and performing deduplication, the titles and abstracts were screened, leading to the exclusion of mechanistic studies, animal experiments, and other irrelevant literature. The full texts of the remaining articles were then assessed for eligibility. Ultimately, 20 studies met the inclusion criteria and were included in the meta-analysis (19–38). The study selection process strictly adhered to the PRISMA guidelines, and the detailed flow diagram is presented in **Figure 1**.

### 3.2 Characteristics of included studies

**Figure 2:**
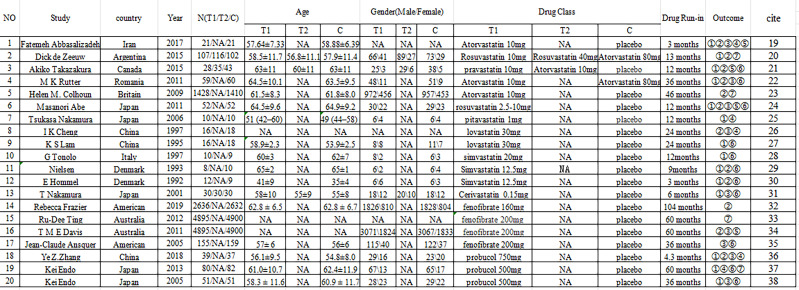
Characteristics of included studies.

A total of 20 RCTs were included in this study, comprising either two-arm or three-arm clinical trials (19–38). These studies collectively involved 29,282 patients diagnosed with DKD complicated by hyperlipidemia. The control groups received either placebo or standard care, while the experimental groups were treated with various types of lipid-lowering agents. Specifically, the included interventions were: Atorvastatin (5 studies; 10/80 mg doses); Rosuvastatin (2 studies; 10/40 mg doses); Simvastatin (3 studies); Lovastatin (2 studies); Fenofibrate (4 studies); Probucol (3 studies); In addition, one study each for Pravastatin, Pitavastatin, and Cerivastatin was included. Among the total included studies, 2 studies were three-arm trials (20, 21), and the remaining 18 studies were two-arm trials. Details are provided in the **Figure 2** and **Supplementary Table 1**.

T1 (treatment group1); T2 (treatment group2); C (control group); N (sample size); NA (not available). ①: Lipid Outcomes; ②: eGFR; ③: Scr; ④: 24hUTP; ⑤: UACR; ⑥: HbA1c; ⑦: Adverse Events.

### 3.3 Results of quality assessment (risk of bias)

**Figure 3:**
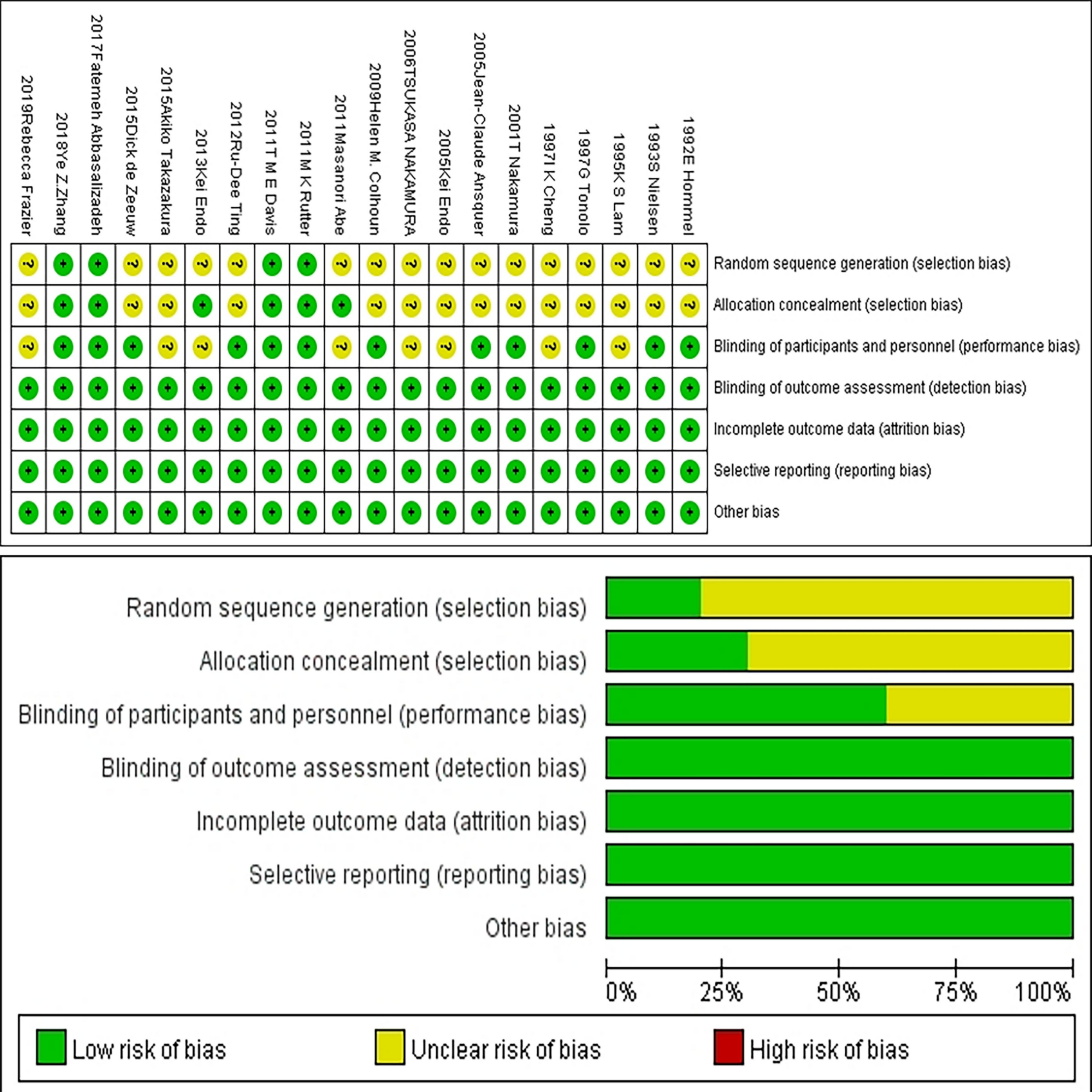
Risks of bias assessment results of included studies.

The risk of bias assessment, conducted using the RoB 2, yielded the following results across the five domains: 1. Randomization-Related Bias: Four studies used a random number table method and were assessed as having a Low risk of bias (19, 22, 34, 36) The remaining studies merely mentioned randomization without specifying the method (20, 21, 23–33, 35, 37), and were therefore assessed as having Uncertain risk (or Some Concerns). 2. Bias Due to Deviations from Intended Interventions (Allocation Concealment): One study reported using the sealed envelope method and was consequently judged to be at Low risk of bias (37). A low risk of bias was assigned to four studies that implemented a centralized randomization method (19, 22, 34, 36). Due to the lack of description regarding allocation concealment, the remaining 15 studies were categorized as having an Uncertain risk of bias. 3. Bias in the Measurement of the Outcome (Blinding of Participants and Outcome Assessors): Five studies used a single-blind method and were assessed as having Uncertain risk (24–27, 37). The remaining studies were double-blind and were assessed as Low risk (18, 20, 22, 23, 28–31, 33–36, 38). 4. Bias Arising from Missing Outcome Data and Bias in Selection of Reported Results: All studies had complete data with no instances of data attrition or drop-out, and no evidence of selective reporting was found. Consequently, these domains were rated as Low risk for all studies. 5. Other Biases: No obvious biases were identified in the other domains. The risk of bias results for the included studies are presented in **Figure 3**.

Risk of bias summary about each risk of bias item for each included study. The graph illustrated the percentage distribution of each risk of bias item among the included studies.

### 3.4 Results of the meta-analysis

#### 3.4.1 Lipid profile improvement

**Figure 4:**
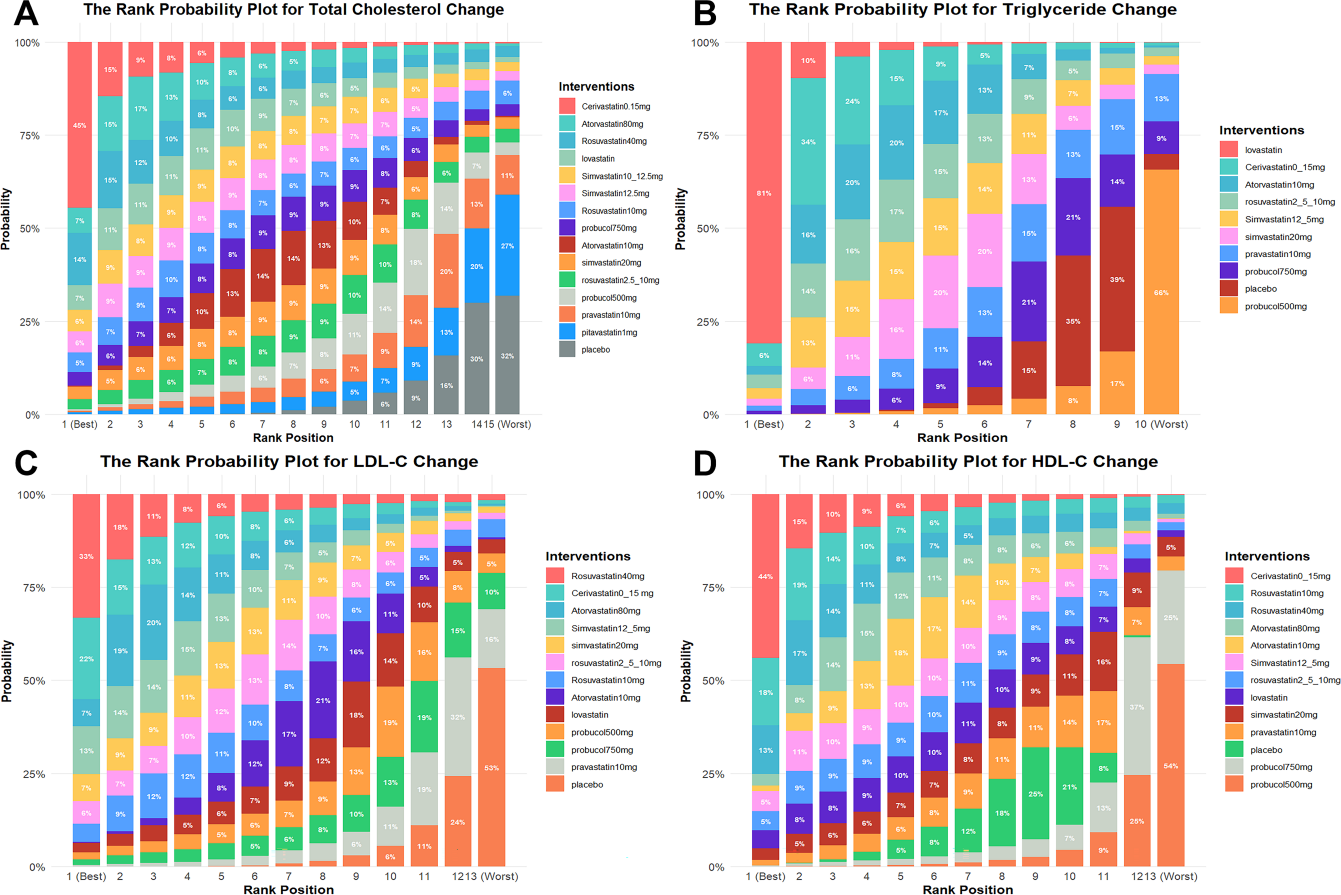
The ranking probability plot. **(A)** TC; **(B)** TG; **(C)** LDL-C; **(D)** HDL-C.

**Figure 5:**
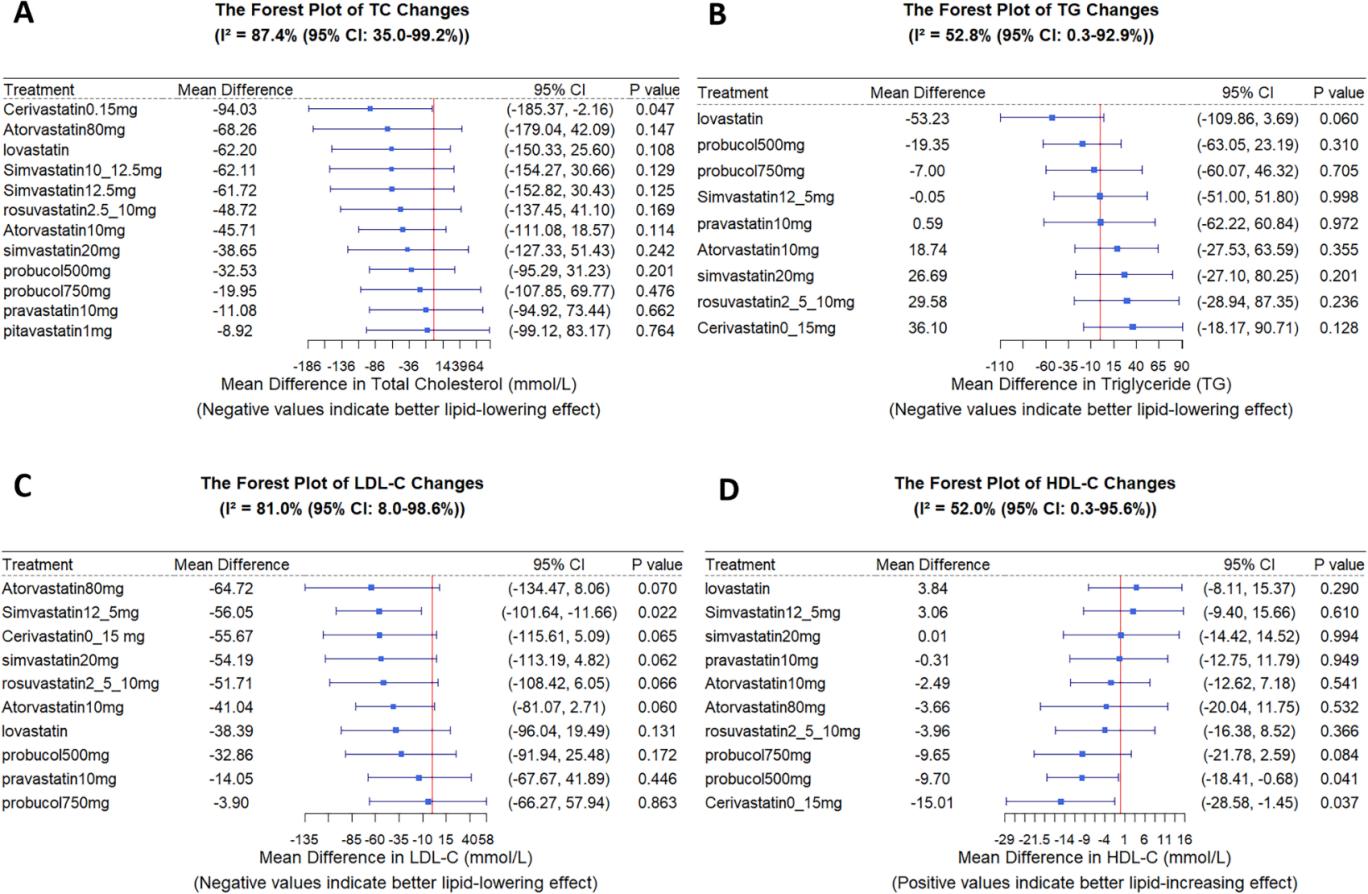
The forest plot. **(A)** TC; **(B)** TG; **(C)** LDL-C; **(D)** HDL-C.

The NMA included 14 studies that reported lipid profile outcomes: TC in 14 studies (19–22, 24, 25, 27–31, 36–38), TC in 12 studies (19–21, 24, 27–31, 36–38), LDL-C in 12 studies (19–22, 24, 27–31, 36, 37), and HDL-C in 13 studies (19–22, 24, 27–31, 36–38). TC Reduction in DKD patients: The Ranking Probability plot (**Figure 4A**) showed that Cerivastatin had the highest probability of being ranked first (45%) in improving TC. The forest plot analysis (**Figure 5A**) further indicated that Cerivastatin was associated with a MD = -94.03, 95% CI: (-185.37 to -2.16) in lowering TC, suggesting it might offer the optimal efficacy for this outcome. TG Reduction in DKD patients: Lovastatin was most likely (probability: 81%) to be the best treatment for lowering TG among DKD patients (**Figure 4B**). The effect size for Lovastatin was MD = -53.23, 95% CI: ((-109.86 to 3.69) **Figure 5B**). Although the Confidence Interval for all interventions included zero, Lovastatin showed the largest point estimate, indicating it is likely the most effective intervention. LDL-C Reduction in DKD patients: The Ranking Probability plot (**Figure 4C**) showed that Rosuvastatin 40 mg had the highest probability of being ranked first (33%), but direct comparison data for this intervention were lacking. The forest plot (**Figure 5C**) suggested that Simvastatin 12.5 mg had the best efficacy, with an MD = -56.05, (95% CI: -101.64 to -11.66). For HDL-C, the 95% Confidence Interval of all interventions crossed the null value (zero), indicating no statistically significant improvement compared with control. Although Lovastatin had the largest point estimate (MD = 3.84, 95% CI: –8.11 to 15.37) in the Forest Plot (**Figure 5D**), the wide Confidence Interval crossing zero precludes any claim of efficacy on HDL‑C; moreover, it did not show a statistically significant advantage in the Ranking Probability Plot (**Figure 4D**).

The x-axis represents the rank order of superiority, the y-axis represents the probability percentage, different colors in the graph denote different interventions, allowing for intuitive comparison of the likelihood of each intervention achieving the top rank for a specific lipid indicator.

This set of forest plots visually compares the effects of different lipid-lowering drugs and dosages on TC, TG, LDL-C, and HDL-C, where each point represents the mean difference, and the horizontal line denotes the 95% CI.

#### 3.4.2 Renal outcomes improvement

Twelve studies reported the post-treatment change in eGFR in DKD patients with hyperlipidemia (19–24, 26, 29, 30, 33, 34, 36). The Ranking Probability plot (**Supplementary Figure 1A**) indicated that Lovastatin showed the most pronounced effect in improving eGFR in DKD patients with hyperlipidemia, with a 47% probability of being ranked first. The forest plot (**Supplementary Figure 2A**) further supported this, showing that Lovastatin yielded the largest Mean Difference (MD=8.76, 95% CI: -10.38 to 27.72), suggesting the highest potential for optimal therapeutic effect. However, its Confidence Interval was notably wide and included zero, which likely reflects the limited sample size and thus low precision of the estimate. Therefore, based on current evidence, the apparent numerical superiority of Lovastatin should be interpreted with caution. Notably, Fenofibrate 54/160mg was ranked low for the eGFR outcome and demonstrated a statistically significant negative effect. However, data from the original study suggested that this observation was attributable to a reversible increase in serum creatinine induced by the drug during the initial treatment phase (32). Long-term follow-up in that study, conversely, indicated that Fenofibrate could slow the rate of eGFR in DKD patients with hyperlipidemia decline. This complex pharmacological characteristic will be explored in depth in the discussion section.

Nine studies provided detailed data on the change in Scr from baseline to post-treatment in DKD patients with hyperlipidemia (19, 22, 24, 26, 31, 33–36, 38). The Forest Plot (**Supplementary Figure 2B**) showed that the Confidence Interval of all reported studies encompassed the null value (zero effect), indicating low certainty in the results. Cerivastatin exhibited the largest point estimate for the Mean Difference (MD = -8.74, 95% CI: -40.85 to 23.23), and The Ranking Probability plot (**Supplementary Figure 1B**) indicated a 22% probability of it being the top-ranked treatment in terms of efficacy.

Five articles reported the change in 24hUTP from baseline to post-treatment in DKD patients with hyperlipidemia. The forest plot (**Supplementary Figure 2C**) demonstrated that the Confidence Interval of all included studies crossed the line of no effect, indicating no statistically significant correlation. Pitavastatin yielded the largest point estimate for the Mean Difference (MD = -55.03, 95% CI: -1239.95 to 1118.08), and The Ranking Probability plot (**Supplementary Figure 1C**) suggested a 27% probability of it being the most efficacious treatment.

Four articles reported pre-treatment and post-treatment UACR values in DKD patients with hyperlipidemia. However, due to the heterogeneity of the reported data, a quantitative meta-analysis could not be completed. Atorvastatin 10 mg was shown to be superior to placebo in reducing UACR in one study (19), though this effect lacked statistical significance. Conversely, in another publication (21), the UACR in the Atorvastatin group was significantly reduced at 12 months. Relative to placebo, Atorvastatin elicited a significantly more pronounced reduction in UACR, an improvement that was sustained throughout the treatment period. Rosuvastatin 2.5—10mg group showed a significant reduction in UACR at 6 months, decreasing from 141 ± 86 mg/gCr to 82 ± 54 mg/gCr (24). The average UACR reduction was 40.1%, which was significantly superior to the 5.8% change observed in the control group. In the article detailing Fenofibrate 200 mg (34), the Fenofibrate group achieved a 23.7% reduction in UACR, which was significantly superior to the 11.5% reduction observed in the placebo group.

#### 3.4.3 Cardiovascular outcomes

In the analysis of CVER, The Ranking Probability plot (**Supplementary Figure 3A**) showed that Probucol 500 mg had a high probability (98%) of being the best treatment for reducing CVER risk in DKD patients with hyperlipidemia. Despite this high ranking, the corresponding Forest Plot (**Supplementary Figure 4A**) indicated a non-significant effect (MD = -2.44, 95% CI: -5.78 to 0.9). Conversely, while the other two interventions showed only moderate rank probabilities, their Forest Plot results demonstrated a statistically significant protective effect: Atorvastatin 10 mg: MD = -3.19, 95% CI: -5.12 to -1.27. Fenofibrate 200mg: MD = -1.44, 95% CI: -2.78 to -0.09. The exceptionally high probability ranking for Probucol may primarily be attributed to zero-event data and the small sample size effect present in its source study. Thus, based on the direct evidence presented in the forest plot, Atorvastatin and Fenofibrate are supported by more robust clinical evidence for reducing CVER in DKD patients with hyperlipidemia.

#### 3.4.4 Safety outcomes

Given the paucity of studies, the pooled effect on mortality was non-significant, with all Confidence Interval crossing the line of no effect. The Ranking Probability Plot (**Supplementary Figure 3B**) showed Atorvastatin had the highest probability (61%) of being the most effective in reducing mortality in DKD patients with hyperlipidemia (MD = -1.54, 95% CI: -3.15 to 0.07) (**Supplementary Figure 4B**).

#### 3.4.5 Other outcomes

Eleven studies reported the change in HbA1c levels in DKD patients with hyperlipidemia from baseline to post-treatment (21, 22, 24, 27–31, 35, 37, 38). Although Atorvastatin80mg showed a relatively larger point estimate in reducing HbA1c compared to other agents (MD = -0.24, 95% CI: -1.66 to 1.18), the Ranking Probability plot (**Supplementary Figure 3C**) and the Forest plot (**Supplementary Figure 4C**) indicated that the 95% CIs for all interventions intersected the line of null effect. This lack of statistical significance across all comparisons suggests considerable uncertainty. Consequently, based on the current body of evidence, no specific lipid-lowering agent can be definitively identified as superior for glycemic control in this population.

### 3.5 Bias analysis

The Funnel plots were generated for all outcome measures. Visual inspection revealed a generally symmetrical distribution of studies, and the Egger’s test results confirmed no significant publication bias (**Supplementary Figures 5**–**7**).

### 3.6 The network and SUCRA plots analysis

For each outcome (blood lipids, renal function, and adverse events), The Network plots were constructed to visualize the geometry of the evidence network formed by the included studies (**Supplementary Figures 8**–**10**). To evaluate the relative efficacy and ranking of interventions, we calculated ranking probabilities and the corresponding SUCRA values (**Supplementary Figures 11**–**13**). Ranking probability plots display the likelihood of each intervention occupying specific ranks, whereas SUCRA values (range 0–1) provide a numeric summary, with higher values indicating superior estimated efficacy. These supplementary figures offer visual and quantitative support for the network meta-analysis methodology and results. The primary conclusions presented in the text remain based on effect estimates and their Confidence Interval.

### 3.7 Twelve-month outcome pooled analysis and leave-one-out sensitivity analysis

Due to the limited number of studies available for other outcomes, which compromised their representativeness, the outcome with the largest number of included studies (TC) was selected as the representative endpoint for further analysis. The Twelve-Month Outcome Pooled Analysis demonstrated a consistent trend of TC reduction across all lipid-lowering therapies, with effect directions aligned with those of the overall analysis (**Supplementary Figure 14**). Although the Confidence Interval widened owing to the smaller sample size, these findings still support the robustness of the overall conclusions. Sensitivity analysis using the leave-one-out method further confirmed that the pooled effect size remained significantly in favor of TC reduction regardless of which study was excluded, with minimal fluctuations, indicating good between-study consistency and that the conclusions were not unduly influenced by any single study (**Supplementary Figure 15**).

## 4 Discussion

This NMA provides an exploratory comparative framework for evaluating lipid-lowering therapies in patients with DKD. While the findings are informative, they should be interpreted as preliminary due to the limited number and size of available RCTs. In fact, according to the GRADE assessment(Tab S2), the certainty of evidence for most lipid profiles (TC, TG, LDL-C, and HDL-C) and key cardiorenal outcomes (such as mortality and CVER) was rated as “low,” primarily reflecting significant heterogeneity and imprecision across the included trials. Notably, although Cerivastatin showed significant lipid-lowering potency in our analysis, it was withdrawn from the global market in 2001 due to a high risk of rhabdomyolysis; thus, its inclusion serves primarily as a historical benchmark for comparative efficacy within the Bayesian model rather than a clinical recommendation. Overall, this study affirms the differing advantages of various agents in lipid profile modulation and establishes a potential ranking of their relative efficacy across cardiorenal outcomes. This cross-class comparison is necessitated by the unique dyslipidemia profile of CKD, characterized by hypertriglyceridemia and impaired HDL metabolism rather than isolated LDL-C elevation (39). In this context, TG-targeting therapies like fibrates are not merely adjuncts but essential for managing residual CRM risk. Our NMA thus rationalizes the comparison between fibrates and statins to address the specific lipid architecture of renal impairment. Our analysis established a hierarchy of efficacy: Lovastatin may offer advantages in improving eGFR and TG in DKD patients with hyperlipidemia; Cerivastatin and Simvastatin demonstrated prominent performance in reducing TC and LDL-C in DKD patients with hyperlipidemia; while Atorvastatin and Fenofibrate possess the most conclusive evidence for reducing the risk of cardiovascular events in DKD patients with hyperlipidemia. However, it must be emphasized that definitive conclusions remain challenging for the majority of renal outcome indicators, constrained by the limited number, size, or quality of existing studies. Efficacy interpretation is further complicated by substantial variations in baseline renal function, lipid profiles, and follow-up durations across trials. Furthermore, the lack of standardized endpoint definitions adds complexity to direct comparisons of renal outcomes. These limitations collectively reflect a current lack of high-quality research in this field and underscore the urgent need for more rigorously designed clinical trials in the future.

Although our analysis identifies Lovastatin as the top-ranked agent for improving eGFR in patients with DKD, this finding must be interpreted as strictly exploratory. The certainty of evidence for renal outcomes and treatment rankings across the Bayesian model was generally rated as low to moderate (Tab S2), primarily due to serious imprecision, characterized by wide confidence intervals and the limited number of large-scale trials. Consequently, the apparent numerical superiority of any specific agent in our rankings should not be overinterpreted as definitive clinical proof, but rather as a hypothesis-generating observation that warrants validation in future high-powered randomized controlled trials. Current evidence concerning the renal effects of statins is complex and often contradictory: Recent meta-analyses confirm that statins can moderately slow the annual decline rate of eGFR in patients with Chronic Kidney Disease (CKD) (40), and this effect for Atorvastatin may be dose-dependent (41, 42). Conversely, observational studies have suggested that high-intensity statins may be associated with an increased risk of acute kidney injury (AKI) (43), and long-term use might even increase the risk of developing DKD (44). This contradiction may stem from multiple factors. The renal effect of statins may not be achieved through a potent direct mechanism, but rather as an indirect manifestation of their powerful cardiovascular protective actions (45). In DKD, a state of high cardiorenal comorbidity, statins preserve renal function indirectly by stabilizing plaques and preventing major cardiovascular events. This protection avoids the secondary damage to the kidney caused by hemodynamic instability (46, 47). Therefore, the core logic for clinical application should be clear: The primary value of Hypolipidemic Agents in DKD is for the prevention of ASCVD. Any subtle influence on eGFR should be viewed as an extension of the cardiovascular benefits, and not as the primary therapeutic target.

Our study precisely demonstrates the “Fenofibrate Paradox”: despite its low ranking for the eGFR outcome due to the reversible elevation of serum creatinine, it was one of only two agents in this analysis proven to significantly reduce the risk of cardiovascular events. This highlights the limitation of assessing its renal effect solely based on short-term eGFR changes. Mechanistic studies suggest that the increase in serum creatinine does not stem from a true decline in glomerular filtration function, but is more likely caused by the PPAR-α agonist altering creatinine secretion in the renal tubules or affecting creatinine generation in muscle (48). More critically, long-term follow-up data from the ACCORD and FIELD trials clearly demonstrate that, despite this initial “pseudo-decline” in eGFR, Fenofibrate treatment is closely associated with a slower long-term eGFR decline rate and a significant reduction in the risk of both micro- and macroalbuminuria when compared to placebo (49, 50). Furthermore, the most recent cohort study data additionally suggest that the use of Fenofibrate is associated with a reduced risk of hospitalization for heart failure (51). This has crucial implications for clinical practice. Therefore, the mild initial elevation of creatinine should be considered an expected, benign phenomenon in clinical practice, and care should be taken to avoid misclassifying it as nephrotoxicity. Monitoring efforts should shift towards long-term eGFR trends and improvement in proteinuria to accurately assess its comprehensive cardiorenal benefits.

While our analysis provides a relative efficacy ranking, its clinical significance must be understood within the broader CRM framework. Even under intensive management of glucose and blood pressure, patients with DKD continue to face a high residual risk of ASCVD (52, 53). The findings of this study offer crucial insights into addressing this specific issue: The value of agents like Atorvastatin or Fenofibrate lies in their capacity to selectively manage this residual risk. The magnitude of these benefits should be contextualized by baseline laboratory levels. Generally, patients with higher baseline LDL-C or TG levels exhibit more pronounced absolute reductions and derive greater clinical benefit from intensive therapy, consistent with the principle that baseline metabolic risk dictates therapeutic gain. For instance, intensified statin therapy remains necessary for patients whose LDL-C levels in DKD are still above target (54); similarly, adding Fenofibrate may provide additional cardiovascular benefits for DKD patients with persistently elevated TG levels (55).

In summary, this meta-analysis serves as a critical benchmark evaluation of traditional lipid-lowering drugs. Its core contribution is defining the role of these agents within the new therapeutic paradigm as key tools for managing “residual risk.” Clinical strategy has now shifted toward multi-target combination therapy, and our results assist physicians in selecting the most appropriate lipid-lowering agent based on the DKD patient’s specific risk phenotype (persistently high LDL-C or high TG), thereby achieving individualized, comprehensive risk management.

The primary strength of this study lies in the use of NMA, which allowed for the simultaneous comparison of multiple lipid-lowering agents in patients with DKD. Furthermore, our adherence to the PRISMA-NMA guidelines ensures methodological rigor. Several limitations inherent to the source literature and our methodology must be acknowledged for this meta-analysis: 1. Limited Scope and Publication Bias: The analysis was restricted to a relatively small number of published RCTs, which limits the comprehensiveness of the findings and introduces the potential for publication bias. 2. Methodological Quality and Risk of Bias: Significant heterogeneity exists regarding study quality. Specifically, earlier studies provided inadequate reporting on methodological details such as randomization and allocation concealment, leading to uncertainty in the risk of bias assessment. 3. Clinical and Statistical Heterogeneity: Substantial clinical and methodological differences across the included trials likely contributed to the observed statistical heterogeneity. The profiles of patients with DKD varied significantly, particularly concerning demographic factors such as ethnicity and the duration of diabetes. Clinically, there were notable differences in baseline renal function, which ranged from early-stage albuminuria to advanced chronic kidney disease, alongside distinct variations in baseline lipid profiles. Methodologically, discrepancies in follow-up duration and inconsistencies in endpoint definitions across studies introduced further variability. Collectively, these multifaceted variations complicate direct comparisons and may significantly influence the generalizability of the therapeutic rankings, underscoring the need for cautious interpretation. 4. Limited Representation of Contemporary Lipid-Lowering Paradigms: The included randomized trials predominantly focus on traditional agents, leaving a significant evidence gap regarding newer therapies such as PCSK9 inhibitors within DKD cohorts. This restriction to an older evidence base means the analysis may not fully capture the synergistic potential of modern, multi-target combination strategies. Consequently, our findings might underestimate the therapeutic ceiling achievable under current clinical guidelines, which increasingly prioritize these contemporary agents to address high residual cardio-renal-metabolic risk. 5. Data Sparsity and Measurement Inconsistency: Data for critical renal outcomes, such as UACR, were sparse or reported using non-uniform metrics and detection methods. This fragmentation prevented effective pooling and resulted in wide Confidence Intervals. Such limitations align with our GRADE evaluation, where major outcomes were downgraded due to serious imprecision and inconsistency. These findings highlight an evolving evidence landscape and underscore the inherent uncertainty in current conclusions regarding lipid management in diabetic nephropathy. 6. Variability in Sample Size: The substantial range in study sample sizes (from 8 to 4,900) further exacerbated both the overall heterogeneity and the width of the Confidence Interval, thereby impacting the precision and the accuracy of the overall effect inference. 7. Finally, most RCTs reported cardiorenal outcomes independently. The lack of integrated cardio-renal dysfunction as a composite endpoint may underrepresent the synergistic benefits of lipid-lowering therapy on the bidirectional axis. 8. Incomplete Reporting of Baseline Cardiorenal Confounders: There is a widespread lack of granular data concerning baseline cardiovascular events and the concurrent use of foundational therapies, such as renin-angiotensin system (RAS) inhibitors or sodium-glucose cotransporter 2 (SGLT2) inhibitors, across the included studies. Without access to these critical baseline parameters, the analysis cannot fully adjust for the influence of background medications on the observed cardiorenal outcomes. This reporting gap introduces potential confounding bias and may limit the generalizability of our therapeutic rankings to contemporary patients managed with current standard-of-care background therapy.

## 5 Conclusion

This NMA achieves a comprehensive comparative evaluation of lipid-lowering therapies in DKD. The results reveal a distinct divergence between surrogate lipid-lowering potency and clinical endpoints. Specifically, our findings indicate that while simvastatin demonstrates the most pronounced efficacy in reducing LDL-C levels, atorvastatin and fenofibrate provide relatively more consistent evidence within this model for reducing the risk of CVER. Furthermore, this analysis clarifies the “Fenofibrate Paradox” by suggesting that its transient impact on serum creatinine reflects a benign hemodynamic adjustment rather than true nephrotoxicity. This observation supports the role of fenofibrate in long-term cardiovascular risk management for renal patients. Within the CRM framework, traditional lipid-lowering agents remain an independent and essential component of therapy. Given the “low” to “moderate” certainty of evidence, these results should be interpreted as an exploratory foundation for phenotype-driven decision-making. In clinical practice, intensive statin therapy should be prioritized for LDL-C dominant risk, while fenofibrate may be more appropriate for addressing hypertriglyceridemia. These targeted strategies are crucial to mitigate the integrated burden of cardiorenal complications in patients with DKD.

## Data Availability

The original contributions presented in the study are included in the article/**Supplementary Material**. Further inquiries can be directed to the corresponding author/s.

## References

[1] Liu D Chen X He W Lu M Li Q Zhang S . Update on the pathogenesis, diagnosis, and treatment of diabetic tubulopathy. Integr Med Nephrol Androl. (2024) 11(4):e23–00029. doi: 10.1097/IMNA-D-23-00029

[2] Deng Y Zhu H Xing J Gao J Duan J Liu P . The role of natural products in improving lipid metabolism disorder-induced mitochondrial dysfunction of diabetic kidney disease. Front Physiol. (2025) 16:1624077. doi: 10.3389/fphys.2025.1624077 40630391 PMC12234459

[3] Chinese medicine for treating diabetic kidney disease by regulating DNA methylation. Integr Med Nephrol Androl. (2024) 11(3):e24–00018. doi: 10.1097/IMNA-D-24-00018

[4] Cao L An Y Liu H Jiang J Liu W Zhou Y . Global epidemiology of type 2 diabetes in patients with NAFLD or MAFLD: a systematic review and meta-analysis. BMC Med. (2024) 22(1):101. doi: 10.1186/s12916-024-03315-0. PMID: 38448943 PMC10919055

[5] Jung CY Yoo TH . Pathophysiologic mechanisms and potential biomarkers in diabetic kidney disease. Diabetes Metab J. (2022) 46(2):181–97. doi: 10.4093/dmj.2021.0329. PMID: 35385633 PMC8987689

[6] Li X Zhang Y Xing X Li M Liu Y Xu A . Podocyte injury of diabetic nephropathy: Novel mechanism discovery and therapeutic prospects. BioMed Pharmacother. (2023) 168:115670. doi: 10.1016/j.biopha.2023.115670. PMID: 37837883

[7] Samsu N . Diabetic nephropathy: Challenges in pathogenesis, diagnosis, and treatment. BioMed Res Int. (2021) 2021:1497449. doi: 10.1155/2021/1497449. PMID: 34307650 PMC8285185

[8] Crasto W Patel V Davies MJ Khunti K . Prevention of microvascular complications of diabetes. Endocrinol Metab Clin North Am. (2021) 50(3):431–55. 10.1016/j.ecl.2021.05.005 34399955

[9] Zheng G Jin J Wang F Zheng Q Shao J Yao J . Association between atherogenic index of plasma and future risk of cardiovascular disease in individuals with cardiovascular-kidney-metabolic syndrome stages 0-3: a nationwide prospective cohort study. Cardiovasc Diabetol. (2025) 24(1):22. doi: 10.1186/s12933-025-02589-9. PMID: 39827127 PMC11743013

[10] Cosentino F Grant PJ Aboyans V Bailey CJ Ceriello A Delgado V . 2019 ESC guidelines on diabetes, pre-diabetes, and cardiovascular diseases developed in collaboration with the EASD. Eur Heart J. (2020) 41(2):255–323. doi: 10.1093/eurheartj/ehz486. PMID: 31497854

[11] Michos ED Bakris GL Rodbard HW Tuttle KR . Glucagon-like peptide-1 receptor agonists in diabetic kidney disease: A review of their kidney and heart protection. Am J Prev Cardiol. (2023) 14:100502. doi: 10.1016/j.ajpc.2023.100502. PMID: 37313358 PMC10258236

[12] Levin A Ahmed SB Carrero JJ Foster B Francis A Hall RK . Executive summary of the KDIGO 2024 Clinical Practice Guideline for the Evaluation and Management of Chronic Kidney Disease: known knowns and known unknowns. Kidney Int. (2024) 105(4):684–701. doi: 10.1016/j.kint.2023.10.016. PMID: 38519239

[13] American Diabetes Association Professional Practice Committee . 11. Chronic kidney disease and risk management: standards of care in diabetes-2024. Diabetes Care. (2024) 47(Suppl 1):S219–S230. doi: 10.2337/dc24-S011. PMID: 38078574 PMC10725805

[14] Tramontano D Bini S Maiorca C Di Costanzo A Carosi M Castellese J . Renal safety assessment of lipid-lowering drugs: Between old certainties and new questions. Drugs. (2025) 85(6):755–75. doi: 10.1007/s40265-025-02158-0. PMID: 40106181 PMC12098426

[15] Miller M . ACC/AHA lipids & ASCVD guidelines: 2018 update. Metabolism. (2019) 99:116–8. doi: 10.1016/j.metabol.2019.03.008. PMID: 30974110

[16] Singh M McEvoy JW Khan SU Wood DA Graham IM Blumenthal RS . Comparison of transatlantic approaches to lipid management: The AHA/ACC/Multisociety guidelines vs the ESC/EAS guidelines. Mayo Clin Proc. (2020) 95(5):998–1014. doi: 10.1016/j.mayocp.2020.01.011. PMID: 32370858

[17] Zhao J Lu X Wang H Chen Q Wan Y . Associations of the triglyceride-glucose index, triglyceride glucose-body mass index, waist-triglyceride index and modified triglyceride-glucose indices with mortality in cardiovascular-kidney-metabolic syndrome stages 0-4: Evidence from NHANES 1999-2020. J Transl Int Med. doi: 10.1515/jtim-2026-0014 PMC12916274 41727962

[18] Kidney Disease: Improving Global Outcomes (KDIGO) Diabetes Work Group . KDIGO 2022 clinical practice guideline for diabetes management in chronic kidney disease. Kidney International. (2022) 102(5S):S1–S127. doi: 10.1016/j.kint.2022.06.008. PMID: 36272764

[19] Abbasalizadeh F Saleh P Dousti R Piri R Naghavi-Behzad M Abbasalizadeh S . Effects of atorvastatin on proteinuria of type 2 diabetic nephropathy in patients with history of gestational diabetes mellitus: A clinical study. Niger Med J. (2017) 58(2):63–7. doi: 10.4103/0300-1652.219348. PMID: 29269983 PMC5726175

[20] de Zeeuw D Anzalone DA Cain VA Cressman MD Heerspink HJ Molitoris BA . Renal effects of atorvastatin and rosuvastatin in patients with diabetes who have progressive renal disease (PLANET I): a randomised clinical trial. Lancet Diabetes Endocrinol. (2015) 3(3):181–90. doi: 10.1016/S2213-8587(14)70246-3. PMID: 25660356

[21] Takazakura A Sakurai M Bando Y Misu H Takeshita Y Kita Y . Renoprotective effects of atorvastatin compared with pravastatin on progression of early diabetic nephropathy. J Diabetes Investig. (2015) 6(3):346–53. doi: 10.1111/jdi.12296. PMID: 25969721 PMC4420568

[22] Rutter MK Prais HR Charlton-Menys V Gittins M Roberts C Davies RR . Protection against nephropathy in diabetes with atorvastatin (PANDA): a randomized double-blind placebo-controlled trial of high- vs. low-dose atorvastatin(1). Diabetes Med. (2011) 28(1):100–8. doi: 10.1111/j.1464-5491.2010.03139. PMID: 21166851

[23] Colhoun HM Betteridge DJ Durrington PN Hitman GA Neil HA Livingstone SJ . Effects of atorvastatin on kidney outcomes and cardiovascular disease in patients with diabetes: an analysis from the Collaborative Atorvastatin Diabetes Study (CARDS). Am J Kidney Dis. (2009) 54(5):810–9. doi: 10.1053/j.ajkd.2009.03.022. PMID: 19540640

[24] Abe M Maruyama N Okada K Matsumoto S Matsumoto K Soma M . Effects of lipid-lowering therapy with rosuvastatin on kidney function and oxidative stress in patients with diabetic nephropathy. J Atheroscler Thromb. (2011) 18(11):1018–28. doi: 10.5551/jat.9084. PMID: 21921413

[25] Nakamura T Sugaya T Kawagoe Y Ueda Y Osada S Koide H . Effect of pitavastatin on urinary liver-type fatty acid-binding protein levels in patients with early diabetic nephropathy. Diabetes Care. (2005) 28(11):2728–32. doi: 10.2337/diacare.28.11.2728. PMID: 16249547

[26] Cheng IK Lam KS Janus ED Pang RW Lauder IJ . Treatment of hyperlipidaemia in patients with non-insulin-dependent diabetes mellitus with progressive nephropathy. Contrib Nephrol. (1997) 120:79–87. doi: 10.1159/000059826. PMID: 9257050

[27] Lam KS Cheng IK Janus ED Pang RW . Cholesterol-lowering therapy may retard the progression of diabetic nephropathy. Diabetologia. (1996) 39(3):367–8. doi: 10.1007/BF00418356. PMID: 7489845

[28] Tonolo G Ciccarese M Brizzi P Puddu L Secchi G Calvia P . Reduction of albumin excretion rate in normotensive microalbuminuric type 2 diabetic patients during long-term simvastatin treatment. Diabetes Care. (1997) 20(12):1891–5. doi: 10.2337/diacare.20.12.1891. PMID: 9405913

[29] Nielsen S Schmitz O Møller N Pørksen N Klausen IC Alberti KG . Renal function and insulin sensitivity during simvastatin treatment in type 2 (non-insulin-dependent) diabetic patients with microalbuminuria. Diabetologia. (1993) 36(10):1079–86. doi: 10.1007/BF02374502. PMID: 8243858

[30] Hommel E Andersen P Gall MA Nielsen F Jensen B Rossing P . Plasma lipoproteins and renal function during simvastatin treatment in diabetic nephropathy. Diabetologia. (1992) 35(5):447–51. doi: 10.1007/BF02342442. PMID: 1521727

[31] Nakamura T Ushiyama C Hirokawa K Osada S Shimada N Koide H . Effect of cerivastatin on urinary albumin excretion and plasma endothelin-1 concentrations in type 2 diabetes patients with microalbuminuria and dyslipidemia. Am J Nephrol. (2001) 21(6):449–54. doi: 10.1159/000046648. PMID: 11799261

[32] Frazier R Mehta R Cai X Lee J Napoli S Craven T . Associations of fenofibrate therapy with incidence and progression of CKD in patients with type 2 diabetes. Kidney Int Rep. (2018) 4(1):94–102. doi: 10.1016/j.ekir.2018.09.006. PMID: 30596172 PMC6308372

[33] Ting RD Keech AC Drury PL Donoghoe MW Hedley J Jenkins AJ . Benefits and safety of long-term fenofibrate therapy in people with type 2 diabetes and renal impairment: the FIELD Study. Diabetes Care. (2012) 35(2):218–25. doi: 10.2337/dc11-1109. PMID: 22210576 PMC3263870

[34] Davis TM Ting R Best JD Donoghoe MW Drury PL Sullivan DR . Effects of fenofibrate on renal function in patients with type 2 diabetes mellitus: the Fenofibrate Intervention and Event Lowering in Diabetes (FIELD) Study. Diabetologia. (2011) 54(2):280–90. doi: 10.1007/s00125-010-1951-1. PMID: 21052978

[35] Ansquer JC Foucher C Rattier S Taskinen MR Steiner G . Fenofibrate reduces progression to microalbuminuria over 3 years in a placebo-controlled study in type 2 diabetes: results from the Diabetes Atherosclerosis Intervention Study (DAIS). Am J Kidney Dis. (2005) 45(3):485–93. doi: 10.1053/j.ajkd.2004.11.004. PMID: 15754270

[36] Ye Z Zhang L Xiao Z Wang L Cai D Li W . Probucol combined with valsartan in diabetic nephropathy: a 52-week, randomized, double-blind clinical trial. Int J Clin Exp Med. (2018) 11:2161–71.

[37] Endo K Saiki A Yamaguchi T Sakuma K Sasaki H Ban N . Probucol suppresses initiation of chronic hemodialysis therapy and renal dysfunction-related death in diabetic nephropathy patients: Sakura study. J Atheroscler Thromb. (2013) 20(5):494–502. doi: 10.5551/jat.15263. PMID: 23363981

[38] Endo K Miyashita Y Sasaki H Ohira M Saiki A Koide N . Probucol delays progression of diabetic nephropathy. Diabetes Res Clin Pract. (2006) 71(2):156–63. doi: 10.1016/j.diabres.2005.05.012. PMID: 16009446

[39] Naim MAAZ Sumida K Streja E Thomas F Davis RL Kalantar-Zadeh K . Lipid-lowering therapies in patients with chronic kidney disease: A perspective on high-density lipoprotein cholesterol. Drugs. (2026) 86(2):177–202. doi: 10.1007/s40265-025-02275-w. PMID: 41528633

[40] Vedamurthy D Sagheer U Tyagi M Maki KC Kalra DK . Meta-analysis of the impact of statins on change in renal function in patients with moderate or severe chronic kidney disease. J Clin Lipidol. (2025) 19(6):1575–85. doi: 10.1016/j.jacl.2025.09.014. PMID: 41102113

[41] Vogt L Bangalore S Fayyad R Melamed S Hovingh GK DeMicco DA . Atorvastatin has a dose-dependent beneficial effect on kidney function and associated cardiovascular outcomes: Post Hoc analysis of 6 double-blind randomized controlled trials. J Am Heart Assoc. (2019) 8(9):e010827. doi: 10.1161/JAHA.118.010827. PMID: 31020900 PMC6512126

[42] Cannon CP Steinberg BA Murphy SA Mega JL Braunwald E . Meta-analysis of cardiovascular outcomes trials comparing intensive versus moderate statin therapy. J Am Heart Assoc. (2006) 48(3):438–45. doi: 10.1016/j.jacc.2006.04.070. PMID: 16875966

[43] Corrao G Soranna D Casula M Merlino L Porcellini MG Catapano AL . High-potency statins increase the risk of acute kidney injury: evidence from a large population-based study. Atherosclerosis. (2014) 234(1):224–9. doi: 10.1016/j.atherosclerosis.2014.02.022. PMID: 24681912

[44] Guo J Jiang Z Xia Y Wang H Tang Q Meng B . The association between statin use and diabetic nephropathy in US adults: data from NHANES 2005 - 2018. Front Endocrinol (Lausanne). (2024) 15:1381746. doi: 10.3389/fendo.2024.1381746. PMID: 38726340 PMC11079199

[45] Ueki Y Itagaki T Kuwahara K . Lipid-lowering therapy and coronary plaque regression. J Atheroscler Thromb. (2024) 31(11):1479–95. doi: 10.5551/jat.RV22024. PMID: 39111840 PMC11537793

[46] Chou R Cantor A Dana T Wagner J Ahmed AY Fu R . Statin use for the primary prevention of cardiovascular disease in adults: Updated evidence report and systematic review for the US Preventive Services Task Force. Jama. (2022) 328(8):754–71. doi: 10.1001/jama.2022.12138. PMID: 35997724

[47] Ting R-D Keech A . Fenofibrate and renal disease: clinical effects in diabetes. Clin Lipidology. (2013) 8(6):669–80. doi: 10.2217/clp.13.69

[48] American Diabetes Association Professional Practice Committee . 10. Cardiovascular disease and risk management: standards of care in diabetes-2024. Diabetes Care. (2024) 47(Suppl 1):S179–S218. doi: 10.2337/dc24-S010. PMID: 38078592 PMC10725811

[49] Mychaleckyj JC Craven T Nayak U Buse J Crouse JR Elam M . Reversibility of fenofibrate therapy–induced renal function impairment in ACCORD type 2 diabetic participants. Diabetes Care. (2012) 35(5):1008–14. doi: 10.2337/dc11-1811. PMID: 22432114 PMC3329840

[50] Kim JY Kim NH Lee J Kim DH Kim SG . Fenofibrate therapy and risk of heart failure outcomes in patients with Type 2 diabetes: a propensity-matched cohort study. Eur Heart J Cardiovasc Pharmacother. (2025) 11(7):620–9. doi: 10.1093/ehjcvp/pvaf053. PMID: 40685251 PMC12582659

[51] Tu QM Jin HM Yang XH . Lipid abnormality in diabetic kidney disease and potential treatment advancements. Front Endocrinol (Lausanne). (2025) 16:1503711. doi: 10.3389/fendo.2025.1503711. PMID: 40171201 PMC11958226

[52] Bhatt DL Steg PG Miller M Brinton EA Jacobson TA Ketchum SB . Cardiovascular risk reduction with icosapent ethyl for hypertriglyceridemia. N Engl J Med. (2019) 380(1):11–22. doi: 10.1056/NEJMoa1812792. PMID: 30415628

[53] Yang X Su G Zhang T Yang H Tao H Du X . Comparison of admission glycemic variability and glycosylated hemoglobin in predicting major adverse cardiac events among type 2 diabetes patients with heart failure following acute ST-segment elevation myocardial infarction. J Transl Int Med. (2024) 12:188–96. doi:– 10.2478/jtim-2024-0006 PMC11229884 38978967

[54] Ezhov MV Arutyunov GP . Effectiveness and safety of fenofibrate in routine treatment of patients with hypertriglyceridemia and metabolic syndrome. Diseases. (2013) 11(4):140. doi: 10.3390/diseases11040140. PMID: 37873784 PMC10594425

[55] Cai X Huang H Li T . Cardiovascular-kidney-metabolic: Hype or a focus on front-line health?. J Transl Int Med. (2025) 13:512–5. doi: 10.1515/jtim-2025-0049 PMC12721360 41438464

