# Supplementary material for: Comparative efficacy of lipid-lowering therapies on the cardio-renal-metabolic axis in diabetic kidney disease: a Bayesian network meta-analysis addressing residual CRM risk
Source: Front Endocrinol (Lausanne). 2026 May 29;17:1842847. doi: 10.3389/fendo.2026.1842847 (PMC13259902; doi:10.3389/fendo.2026.1842847)
Supplement: Supplementary file 1 [file DataSheet1.docx]

**Comparative Efficacy of Lipid-Lowering Therapies on the Cardio-Renal-Metabolic Axis in Diabetic Kidney Disease: A Bayesian Network Meta-analysis Addressing Residual CRM Risk**

Jun Luo^1#^, Yinzhong Dai^1,2#^, Chenguang Wu^1#^, Chengying Lan^1^, Junwei Shi^1^,Jinfeng Qi^1^, Shimei Hua^1^, Yan An^1^, Lifan Wang^1*^, Ping Li^3*^, Peng Liu^2*^

^1^ Renal Division, Department of Medicine, Heilongjiang Academy of Chinese Medicine Sciences, Harbin, China

^2^ Beijing Key Lab for Immune-Mediated Inflammatory Diseases, China-Japan Friendship Hospital, Beijing, China

^3^ Xiyuan Hospital, China Academy of Chinese Medical Sciences, Beijing, 100091, China.

^#^These authors contributed equally to this work and shared first authorship.

^*^ Corresponding author

Lifan Wang, PhD, Professor, Renal Division, Department of Medicine, Heilongjiang

Academy of Chinese Medicine Sciences, Harbin, 150036, China, E-mail: wlf4648374@163.com

Ping Li, PhD, Professor, Beijing Key Lab for Immune-Mediated Inflammatory Diseases, China-Japan Friendship Hospital, Beijing, 10002, China, E-mail:lp8675@163.com

Peng Liu, PhD, Associate Professor, Department of Nephrology, Xiyuan Hospital, China Academy of Chinese Medical Sciences, Beijing, 100091, China, E-mail: 2025n024@zcmu.edu.cn

**Contents legend:**

**1. Search strategy**: The complete electronic search strategy for the four English databases (PubMed, Web of Science, Embase, and Cochrane Library).................................................................................................................**S3-S41**

**2.Tab. S1.** Characteristics of Included Studies..........................................................**S41**

**3.Tab. S2** Certainty of Evidence for Outcome Measures of Lipid-Lowering Drugs.**S42**

**4.Fig. S1.** The ranking probability plot. A: eGFR; B: SCR; C: UPR............................................................................................................................**S43**

**5.Fig. S2.** The Forest Plot. A: eGFR; B: Scr; C: UPR...............................................**S44**

**6.Fig. S3.** The ranking probability plot. A: Cardiovascular Events; B: Death Events; C: HbA1c .......................................................................................................................**S45**

**7.Fig. S4.** The Forest Plot. A: Cardiovascular Events; B: Death Events; C: HbA1c........................................................................................................................**S46**

**8.Fig. S5.** The Funnel Plot. A: TC; B: TG; C: LDL-C; D: HDL-C...........................**S47**

**9.Fig. S6** The Funnel Plot. A: eGFR; B: SCR; C: UPR.............................................**S48**

**10.Fig. S7** The Funnel Plot. A: Cardiovascular Events; B: Death Events; C: HbA1c........................................................................................................................**S49**

**11.Fig. S8.** The Network Plot A: TC; B: TG; C: LDL-C; D: HDL-C.......................................................................................................................**S50**

**12.Fig. S9.** The Network Plot. A: eGFR; B: SCR; C: UPR.......................................**S51**

**13.Fig. S10.** The Network Plot. A: HbA1C; B: Cardiovascular Events; C: Death Events.........................................................................................................................**S52**

**14.Fig. S11.** The SUCRA Plot. A: TC; B: TG; C: LDL-C; D: HDL-C.....................**S53**

**15.Fig. S12.** The SUCRA Plot. A: eGFR; B: SCR; C: UPR......................................**S54**

**16.Fig. S13.** The SUCRA Plot. A: HbA1c; B: Cardiovascular Events; C: Death Events.........................................................................................................................**S55**

**17.Fig. S14.** The Forest Plot of TC Changes After Treatment in 12 Months............**S56**

**18.Fig. S15.** The Sensitivity Analysis Forest Plot (Leave-One-Out Method)...........**S57**

**Search strategy**

1. **Pubmed**

#1. "Hypolipidemic Agents"[Mesh] 66,433

#2. (((((((((((((((Hypolipidemic Agents[Title/Abstract]) OR (Agents, Hypolipidemic[Title/Abstract])) OR (Antihyperlipidemic[Title/Abstract])) OR (Antilipemic Drug*[Title/Abstract])) OR (Drug*, Antilipemic[Title/Abstract])) OR (Antihyperlipemics[Title/Abstract])) OR (Antihyperlipidemics[Title/Abstract])) OR (Antilipemic Agents[Title/Abstract])) OR (Agents, Antilipemic[Title/Abstract])) OR (Hypolipidemic Drugs[Title/Abstract])) OR (Drugs, Hypolipidemic[Title/Abstract])) OR (Antilipemic*[Title/Abstract])) OR (Hypolipidemic Agent[Title/Abstract])) OR (Agent, Hypolipidemic[Title/Abstract])) OR (Hypolipidemic Drug[Title/Abstract])) OR (Drug, Hypolipidemic[Title/Abstract]) 6,547

#3. ("Hypolipidemic Agents"[Mesh]) OR ((((((((((((((((Hypolipidemic Agents[Title/Abstract]) OR (Agents, Hypolipidemic[Title/Abstract])) OR (Antihyperlipidemic[Title/Abstract])) OR (Antilipemic Drug*[Title/Abstract])) OR (Drug*, Antilipemic[Title/Abstract])) OR (Antihyperlipemics[Title/Abstract])) OR (Antihyperlipidemics[Title/Abstract])) OR (Antilipemic Agents[Title/Abstract])) OR (Agents, Antilipemic[Title/Abstract])) OR (Hypolipidemic Drugs[Title/Abstract])) OR (Drugs, Hypolipidemic[Title/Abstract])) OR (Antilipemic*[Title/Abstract])) OR (Hypolipidemic Agent[Title/Abstract])) OR (Agent, Hypolipidemic[Title/Abstract])) OR (Hypolipidemic Drug[Title/Abstract])) OR (Drug, Hypolipidemic[Title/Abstract])) 69,642

#4. "Hydroxymethylglutaryl-CoA Reductase Inhibitors"[Mesh] 37,079

#5. (((((((((((((((((((((((Hydroxymethylglutaryl-CoA Reductase Inhibitors[Title/Abstract]) OR (Hydroxymethylglutaryl CoA Reductase Inhibitors[Title/Abstract])) OR (Inhibitors, Hydroxymethylglutaryl-CoA Reductase[Title/Abstract])) OR (Reductase Inhibitors, Hydroxymethylglutaryl-CoA[Title/Abstract])) OR (HMG-CoA Reductase Inhibitor[Title/Abstract])) OR (HMG CoA Reductase Inhibitor[Title/Abstract])) OR (Statin*[Title/Abstract])) OR (Inhibitors, HMG-CoA Reductase[Title/Abstract])) OR (Inhibitors, HMG CoA Reductase[Title/Abstract])) OR (Reductase Inhibitors, HMG-CoA[Title/Abstract])) OR (HMG-CoA Reductase Inhibitors[Title/Abstract])) OR (HMG CoA Reductase Inhibitors[Title/Abstract])) OR (Inhibitors, Hydroxymethylglutaryl-Coenzyme A[Title/Abstract])) OR (Hydroxymethylglutaryl-Coenzyme A Inhibitors[Title/Abstract])) OR (Inhibitors, Hydroxymethylglutaryl Coenzyme A[Title/Abstract])) OR (Inhibitors, Hydroxymethylglutaryl-CoA[Title/Abstract])) OR (Hydroxymethylglutaryl-CoA Inhibitors[Title/Abstract])) OR (Inhibitors, Hydroxymethylglutaryl CoA[Title/Abstract])) OR (Hydroxymethylglutaryl-CoA Reductase Inhibitor[Title/Abstract])) OR (Hydroxymethylglutaryl CoA Reductase Inhibitor[Title/Abstract])) OR (Reductase Inhibitor, Hydroxymethylglutaryl-CoA[Title/Abstract])) OR (Statins, HMG-CoA[Title/Abstract])) OR (HMG-CoA Statins[Title/Abstract])) OR (Statins, HMG CoA[Title/Abstract]) 61,067

#6. ("Hydroxymethylglutaryl-CoA Reductase Inhibitors"[Mesh]) OR ((((((((((((((((((((((((Hydroxymethylglutaryl-CoA Reductase Inhibitors[Title/Abstract]) OR (Hydroxymethylglutaryl CoA Reductase Inhibitors[Title/Abstract])) OR (Inhibitors, Hydroxymethylglutaryl-CoA Reductase[Title/Abstract])) OR (Reductase Inhibitors, Hydroxymethylglutaryl-CoA[Title/Abstract])) OR (HMG-CoA Reductase Inhibitor[Title/Abstract])) OR (HMG CoA Reductase Inhibitor[Title/Abstract])) OR (Statin*[Title/Abstract])) OR (Inhibitors, HMG-CoA Reductase[Title/Abstract])) OR (Inhibitors, HMG CoA Reductase[Title/Abstract])) OR (Reductase Inhibitors, HMG-CoA[Title/Abstract])) OR (HMG-CoA Reductase Inhibitors[Title/Abstract])) OR (HMG CoA Reductase Inhibitors[Title/Abstract])) OR (Inhibitors, Hydroxymethylglutaryl-Coenzyme A[Title/Abstract])) OR (Hydroxymethylglutaryl-Coenzyme A Inhibitors[Title/Abstract])) OR (Inhibitors, Hydroxymethylglutaryl Coenzyme A[Title/Abstract])) OR (Inhibitors, Hydroxymethylglutaryl-CoA[Title/Abstract])) OR (Hydroxymethylglutaryl-CoA Inhibitors[Title/Abstract])) OR (Inhibitors, Hydroxymethylglutaryl CoA[Title/Abstract])) OR (Hydroxymethylglutaryl-CoA Reductase Inhibitor[Title/Abstract])) OR (Hydroxymethylglutaryl CoA Reductase Inhibitor[Title/Abstract])) OR (Reductase Inhibitor, Hydroxymethylglutaryl-CoA[Title/Abstract])) OR (Statins, HMG-CoA[Title/Abstract])) OR (HMG-CoA Statins[Title/Abstract])) OR (Statins, HMG CoA[Title/Abstract])) 70,050

#7. "Atorvastatin"[Mesh] 7,910

#8. (((((((((((Atorvastatin[Title/Abstract]) OR ((3R,5R)-7-(2-(4-Fluorophenyl)-5-isopropyl-3-phenyl-4-(phenylcarbamoyl)-1H-pyrrol-1-yl)-3,5-dihydroxyheptanoic acid[Title/Abstract])) OR (Lipitor[Title/Abstract])) OR (Atorvastatin Calcium[Title/Abstract])) OR (Atorvastatin, Calcium Salt[Title/Abstract])) OR (Atorvastatin Calcium Anhydrous[Title/Abstract])) OR (Liptonorm[Title/Abstract])) OR (Atorvastatin Calcium Hydrate[Title/Abstract])) OR (CI 981[Title/Abstract])) OR (CI-981[Title/Abstract])) OR (CI981[Title/Abstract])) OR (Atorvastatin Calcium Trihydrate[Title/Abstract]) 11,440

#9. ("Atorvastatin"[Mesh]) OR ((((((((((((Atorvastatin[Title/Abstract]) OR ((3R,5R)-7-(2-(4-Fluorophenyl)-5-isopropyl-3-phenyl-4-(phenylcarbamoyl)-1H-pyrrol-1-yl)-3,5-dihydroxyheptanoic acid[Title/Abstract])) OR (Lipitor[Title/Abstract])) OR (Atorvastatin Calcium[Title/Abstract])) OR (Atorvastatin, Calcium Salt[Title/Abstract])) OR (Atorvastatin Calcium Anhydrous[Title/Abstract])) OR (Liptonorm[Title/Abstract])) OR (Atorvastatin Calcium Hydrate[Title/Abstract])) OR (CI 981[Title/Abstract])) OR (CI-981[Title/Abstract])) OR (CI981[Title/Abstract])) OR (Atorvastatin Calcium Trihydrate[Title/Abstract])) 12,528

#10. "Simvastatin"[Mesh] 8,747

#11. (((((Simvastatin[Title/Abstract]) OR (Zocor[Title/Abstract])) OR (MK-733[Title/Abstract])) OR (MK733[Title/Abstract])) OR (MK 733[Title/Abstract])) OR (Synvinolin[Title/Abstract]) 11,539

#12. ("Simvastatin"[Mesh]) OR ((((((Simvastatin[Title/Abstract]) OR (Zocor[Title/Abstract])) OR (MK-733[Title/Abstract])) OR (MK733[Title/Abstract])) OR (MK 733[Title/Abstract])) OR (Synvinolin[Title/Abstract])) 12,968

#13. "Rosuvastatin Calcium"[Mesh] 3,203

#14. (((((Rosuvastatin Calcium[Title/Abstract]) OR (Calcium, Rosuvastatin[Title/Abstract])) OR (Rosuvastatin[Title/Abstract])) OR (ZD4522[Title/Abstract])) OR (ZD 4522[Title/Abstract])) OR (Crestor[Title/Abstract]) 4,905

#15. ("Rosuvastatin Calcium"[Mesh]) OR ((((((Rosuvastatin Calcium[Title/Abstract]) OR (Calcium, Rosuvastatin[Title/Abstract])) OR (Rosuvastatin[Title/Abstract])) OR (ZD4522[Title/Abstract])) OR (ZD 4522[Title/Abstract])) OR (Crestor[Title/Abstract])) 5,263

#16. "Lovastatin"[Mesh] 12,363

#17. (((((((((((Lovastatin[Title/Abstract]) OR (6 Methylcompactin[Title/Abstract])) OR (Mevinolin[Title/Abstract])) OR (Monacolin K[Title/Abstract])) OR (Lovastatin, 1 alpha-Isomer[Title/Abstract])) OR (1 alpha-Isomer Lovastatin[Title/Abstract])) OR (alpha-Isomer Lovastatin, 1[Title/Abstract])) OR (Lovastatin, 1 alpha Isomer[Title/Abstract])) OR (MK-803[Title/Abstract])) OR (MK803[Title/Abstract])) OR (MK 803[Title/Abstract])) OR (Mevacor[Title/Abstract]) 4,948

#18. ("Lovastatin"[Mesh]) OR ((((((((((((Lovastatin[Title/Abstract]) OR (6 Methylcompactin[Title/Abstract])) OR (Mevinolin[Title/Abstract])) OR (Monacolin K[Title/Abstract])) OR (Lovastatin, 1 alpha-Isomer[Title/Abstract])) OR (1 alpha-Isomer Lovastatin[Title/Abstract])) OR (alpha-Isomer Lovastatin, 1[Title/Abstract])) OR (Lovastatin, 1 alpha Isomer[Title/Abstract])) OR (MK-803[Title/Abstract])) OR (MK803[Title/Abstract])) OR (MK 803[Title/Abstract])) OR (Mevacor[Title/Abstract])) 14,213

#19. "Pravastatin"[Mesh] 3,597

#20. ((((((((((((((((((((((((((((Pravastatin[Title/Abstract]) OR (Eptastatin[Title/Abstract])) OR (SQ-31000[Title/Abstract])) OR (SQ 31000[Title/Abstract])) OR (SQ-31,000[Title/Abstract])) OR (SQ 31,000[Title/Abstract])) OR (SQ31,000[Title/Abstract])) OR (Pravastatin tert-Octylamine Salt[Title/Abstract])) OR (Pravastatin tert Octylamine Salt[Title/Abstract])) OR (Pravastatin Sodium[Title/Abstract])) OR (Pravastatin Sodium Salt[Title/Abstract])) OR (Sodium Salt, Pravastatin[Title/Abstract])) OR (Pravastatin, (6 beta)-Isomer[Title/Abstract])) OR (Pravastatin Monosodium Salt, (6 beta)-Isomer[Title/Abstract])) OR (CS-514[Title/Abstract])) OR (CS514[Title/Abstract])) OR (CS 514[Title/Abstract])) OR (Pravachol[Title/Abstract])) OR (Pravacol[Title/Abstract])) OR (Lipostat[Title/Abstract])) OR (Elisor[Title/Abstract])) OR (RMS-431[Title/Abstract])) OR (RMS431[Title/Abstract])) OR (RMS 431[Title/Abstract])) OR (Apo Pravastatin[Title/Abstract])) OR (Mevalotin[Title/Abstract])) OR (Lin Pravastatin[Title/Abstract])) OR (Vasten[Title/Abstract])) OR (Nu Pravastatin[Title/Abstract]) 4,562

#21. ("Pravastatin"[Mesh]) OR (((((((((((((((((((((((((((((Pravastatin[Title/Abstract]) OR (Eptastatin[Title/Abstract])) OR (SQ-31000[Title/Abstract])) OR (SQ 31000[Title/Abstract])) OR (SQ-31,000[Title/Abstract])) OR (SQ 31,000[Title/Abstract])) OR (SQ31,000[Title/Abstract])) OR (Pravastatin tert-Octylamine Salt[Title/Abstract])) OR (Pravastatin tert Octylamine Salt[Title/Abstract])) OR (Pravastatin Sodium[Title/Abstract])) OR (Pravastatin Sodium Salt[Title/Abstract])) OR (Sodium Salt, Pravastatin[Title/Abstract])) OR (Pravastatin, (6 beta)-Isomer[Title/Abstract])) OR (Pravastatin Monosodium Salt, (6 beta)-Isomer[Title/Abstract])) OR (CS-514[Title/Abstract])) OR (CS514[Title/Abstract])) OR (CS 514[Title/Abstract])) OR (Pravachol[Title/Abstract])) OR (Pravacol[Title/Abstract])) OR (Lipostat[Title/Abstract])) OR (Elisor[Title/Abstract])) OR (RMS-431[Title/Abstract])) OR (RMS431[Title/Abstract])) OR (RMS 431[Title/Abstract])) OR (Apo Pravastatin[Title/Abstract])) OR (Mevalotin[Title/Abstract])) OR (Lin Pravastatin[Title/Abstract])) OR (Vasten[Title/Abstract])) OR (Nu Pravastatin[Title/Abstract])) 5,354

#22. "Fluvastatin"[Mesh] 1,489

#23. ((((((((((Fluvastatin[Title/Abstract]) OR (Lescol[Title/Abstract])) OR (XU 62-320[Title/Abstract])) OR (XU 62 320[Title/Abstract])) OR (XU 62320[Title/Abstract])) OR (XU-62320[Title/Abstract])) OR (XU62320[Title/Abstract])) OR (Fluvastatin Sodium[Title/Abstract])) OR (Fluindostatin[Title/Abstract])) OR (Fluvastatin Sodium Salt[Title/Abstract])) OR (7-(3-(4-Fluorophenyl)-1-(1-methylethyl)-1H-indol-2-yl)-3,5-dihydroxy-6-heptenoate[Title/Abstract]) 2,166

#24. ("Fluvastatin"[Mesh]) OR (((((((((((Fluvastatin[Title/Abstract]) OR (Lescol[Title/Abstract])) OR (XU 62-320[Title/Abstract])) OR (XU 62 320[Title/Abstract])) OR (XU 62320[Title/Abstract])) OR (XU-62320[Title/Abstract])) OR (XU62320[Title/Abstract])) OR (Fluvastatin Sodium[Title/Abstract])) OR (Fluindostatin[Title/Abstract])) OR (Fluvastatin Sodium Salt[Title/Abstract])) OR (7-(3-(4-Fluorophenyl)-1-(1-methylethyl)-1H-indol-2-yl)-3,5-dihydroxy-6-heptenoate[Title/Abstract])) 2,355

#25. "pitavastatin" [Supplementary Concept] 783

#26. ((((((((((pitavastatin[Title/Abstract]) OR ((E,3R,5S)-7-(2-cyclopropyl-4-(4-fluorophenyl)quinolin-3-yl)-3,5-dihydroxyhept-6-enoic acid[Title/Abstract])) OR (itavastatin[Title/Abstract])) OR (NK 104[Title/Abstract])) OR (NK-104[Title/Abstract])) OR (P 872441[Title/Abstract])) OR (P-872441[Title/Abstract])) OR (pitavastatin lactone[Title/Abstract])) OR (nisvastatin[Title/Abstract])) OR (pitavastatin calcium[Title/Abstract])) OR (itavastatin calcium[Title/Abstract]) 1,290

#27. ("pitavastatin" [Supplementary Concept]) OR (((((((((((pitavastatin[Title/Abstract]) OR ((E,3R,5S)-7-(2-cyclopropyl-4-(4-fluorophenyl)quinolin-3-yl)-3,5-dihydroxyhept-6-enoic acid[Title/Abstract])) OR (itavastatin[Title/Abstract])) OR (NK 104[Title/Abstract])) OR (NK-104[Title/Abstract])) OR (P 872441[Title/Abstract])) OR (P-872441[Title/Abstract])) OR (pitavastatin lactone[Title/Abstract])) OR (nisvastatin[Title/Abstract])) OR (pitavastatin calcium[Title/Abstract])) OR (itavastatin calcium[Title/Abstract])) 1,343

#28. "Fibric Acids"[Mesh] 10,473

#29. (((((((((Fibric Acids[Title/Abstract]) OR (2-Phenoxy Isobutyric Acids[Title/Abstract])) OR (2 Phenoxy Isobutyric Acids[Title/Abstract])) OR (Isobutyric Acids, 2-Phenoxy[Title/Abstract])) OR (2 Phenoxy 2 Methylpropionic Acid Derivatives[Title/Abstract])) OR (Fibric Acid Derivatives[Title/Abstract])) OR (Acid Derivatives, Fibric[Title/Abstract])) OR (Methyl 2 Phenoxypropanoic Acid Derivatives[Title/Abstract])) OR (2-Phenoxy-2-Methylpropionic Acid Derivatives[Title/Abstract])) OR (Fibrate*[Title/Abstract]) 4,207

#30. ("Fibric Acids"[Mesh]) OR ((((((((((Fibric Acids[Title/Abstract]) OR (2-Phenoxy Isobutyric Acids[Title/Abstract])) OR (2 Phenoxy Isobutyric Acids[Title/Abstract])) OR (Isobutyric Acids, 2-Phenoxy[Title/Abstract])) OR (2 Phenoxy 2 Methylpropionic Acid Derivatives[Title/Abstract])) OR (Fibric Acid Derivatives[Title/Abstract])) OR (Acid Derivatives, Fibric[Title/Abstract])) OR (Methyl 2 Phenoxypropanoic Acid Derivatives[Title/Abstract])) OR (2-Phenoxy-2-Methylpropionic Acid Derivatives[Title/Abstract])) OR (Fibrate*[Title/Abstract])) 12,951

#31. "Fenofibrate"[Mesh] 3,102

#32. ((((((((((((((((((((((((((((((((((((((((((((Fenofibrate[Title/Abstract]) OR (Procetofen[Title/Abstract])) OR (Phenofibrate[Title/Abstract])) OR (Procetofene[Title/Abstract])) OR (Lipanthyl[Title/Abstract])) OR (Fe?nofibrate Debat[Title/Abstract])) OR (Debat, Fe?nofibrate[Title/Abstract])) OR (Lipantil[Title/Abstract])) OR (Lipidil-Ter[Title/Abstract])) OR (Lipidil Ter[Title/Abstract])) OR (Lipidil[Title/Abstract])) OR (Secalip[Title/Abstract])) OR (Fenofibrat FPh[Title/Abstract])) OR (LF-178[Title/Abstract])) OR (LF178[Title/Abstract])) OR (LF 178[Title/Abstract])) OR (Apo Feno Micro[Title/Abstract])) OR (Apo-Fenofibrate[Title/Abstract])) OR (Apo Fenofibrate[Title/Abstract])) OR (CiL[Title/Abstract])) OR (Controlip[Title/Abstract])) OR (Fe?nofibrate MSD[Title/Abstract])) OR (Gen Fenofibrate[Title/Abstract])) OR (Livesan[Title/Abstract])) OR (MTW Fenofibrat[Title/Abstract])) OR (Tricor[Title/Abstract])) OR (Normalip[Title/Abstract])) OR (Novo Fenofibrate[Title/Abstract])) OR (Nu Fenofibrate[Title/Abstract])) OR (PMS Fenofibrate Micro[Title/Abstract])) OR (Fenofibrat AbZ[Title/Abstract])) OR (Fenofibrat AL[Title/Abstract])) OR (Fenofibrat AZU[Title/Abstract])) OR (AZU, Fenofibrat[Title/Abstract])) OR (Fenofibrat Heumann[Title/Abstract])) OR (Heumann, Fenofibrat[Title/Abstract])) OR (Fenofibrat Hexal[Title/Abstract])) OR (Hexal, Fenofibrat[Title/Abstract])) OR (Fenofibrat Stada[Title/Abstract])) OR (Stada, Fenofibrat[Title/Abstract])) OR (fenofibrat von ct[Title/Abstract])) OR (Fenofibrat ratiopharm[Title/Abstract])) OR (Antara Micronized Procetofen[Title/Abstract])) OR (Micronized Procetofen, Antara[Title/Abstract])) OR (Procetofen, Antara Micronized[Title/Abstract]) 4,993

#33. ("Fenofibrate"[Mesh]) OR (((((((((((((((((((((((((((((((((((((((((((((Fenofibrate[Title/Abstract]) OR (Procetofen[Title/Abstract])) OR (Phenofibrate[Title/Abstract])) OR (Procetofene[Title/Abstract])) OR (Lipanthyl[Title/Abstract])) OR (Fe?nofibrate Debat[Title/Abstract])) OR (Debat, Fe?nofibrate[Title/Abstract])) OR (Lipantil[Title/Abstract])) OR (Lipidil-Ter[Title/Abstract])) OR (Lipidil Ter[Title/Abstract])) OR (Lipidil[Title/Abstract])) OR (Secalip[Title/Abstract])) OR (Fenofibrat FPh[Title/Abstract])) OR (LF-178[Title/Abstract])) OR (LF178[Title/Abstract])) OR (LF 178[Title/Abstract])) OR (Apo Feno Micro[Title/Abstract])) OR (Apo-Fenofibrate[Title/Abstract])) OR (Apo Fenofibrate[Title/Abstract])) OR (CiL[Title/Abstract])) OR (Controlip[Title/Abstract])) OR (Fe?nofibrate MSD[Title/Abstract])) OR (Gen Fenofibrate[Title/Abstract])) OR (Livesan[Title/Abstract])) OR (MTW Fenofibrat[Title/Abstract])) OR (Tricor[Title/Abstract])) OR (Normalip[Title/Abstract])) OR (Novo Fenofibrate[Title/Abstract])) OR (Nu Fenofibrate[Title/Abstract])) OR (PMS Fenofibrate Micro[Title/Abstract])) OR (Fenofibrat AbZ[Title/Abstract])) OR (Fenofibrat AL[Title/Abstract])) OR (Fenofibrat AZU[Title/Abstract])) OR (AZU, Fenofibrat[Title/Abstract])) OR (Fenofibrat Heumann[Title/Abstract])) OR (Heumann, Fenofibrat[Title/Abstract])) OR (Fenofibrat Hexal[Title/Abstract])) OR (Hexal, Fenofibrat[Title/Abstract])) OR (Fenofibrat Stada[Title/Abstract])) OR (Stada, Fenofibrat[Title/Abstract])) OR (fenofibrat von ct[Title/Abstract])) OR (Fenofibrat ratiopharm[Title/Abstract])) OR (Antara Micronized Procetofen[Title/Abstract])) OR (Micronized Procetofen, Antara[Title/Abstract])) OR (Procetofen, Antara Micronized[Title/Abstract])) 5,439

#34. "Gemfibrozil"[Mesh] 1,448

#35. (((((((((((((((((((((((((((((((((Gemfibrozil[Title/Abstract]) OR (Gemfibrosil[Title/Abstract])) OR (Lopid[Title/Abstract])) OR (Lopid R[Title/Abstract])) OR (Lipur[Title/Abstract])) OR (CI-719[Title/Abstract])) OR (CI719[Title/Abstract])) OR (CI 719[Title/Abstract])) OR (Apo-Gemfibrozil[Title/Abstract])) OR (Apo Gemfibrozil[Title/Abstract])) OR (Ausgem[Title/Abstract])) OR (Bolutol[Title/Abstract])) OR (Chem mart Gemfibrozil[Title/Abstract])) OR (DBL Gemfibrozil[Title/Abstract])) OR (Decrelip[Title/Abstract])) OR (Gemfi 1A Pharma[Title/Abstract])) OR (Gemfibrozilo Ur[Title/Abstract])) OR (Gen Gemfibrozil[Title/Abstract])) OR (GenRX Gemfibrozil[Title/Abstract])) OR (Gemfibrozil, GenRX[Title/Abstract])) OR (Healthsense Gemfibrozil[Title/Abstract])) OR (Gemfibrozil, Healthsense[Title/Abstract])) OR (Jezil[Title/Abstract])) OR (Lipazil[Title/Abstract])) OR (Lipox Gemfi[Title/Abstract])) OR (Litarek[Title/Abstract])) OR (Novo Gemfibrozil[Title/Abstract])) OR (Nu Gemfibrozil[Title/Abstract])) OR (Pilder[Title/Abstract])) OR (PMS Gemfibrozil[Title/Abstract])) OR (SBPA Gemfibrozil[Title/Abstract])) OR (Gemfibrozil, SBPA[Title/Abstract])) OR (Terry White Chemists Gemfibrozil[Title/Abstract])) OR (Trialmin[Title/Abstract]) 2,111

#36. ("Gemfibrozil"[Mesh]) OR ((((((((((((((((((((((((((((((((((Gemfibrozil[Title/Abstract]) OR (Gemfibrosil[Title/Abstract])) OR (Lopid[Title/Abstract])) OR (Lopid R[Title/Abstract])) OR (Lipur[Title/Abstract])) OR (CI-719[Title/Abstract])) OR (CI719[Title/Abstract])) OR (CI 719[Title/Abstract])) OR (Apo-Gemfibrozil[Title/Abstract])) OR (Apo Gemfibrozil[Title/Abstract])) OR (Ausgem[Title/Abstract])) OR (Bolutol[Title/Abstract])) OR (Chem mart Gemfibrozil[Title/Abstract])) OR (DBL Gemfibrozil[Title/Abstract])) OR (Decrelip[Title/Abstract])) OR (Gemfi 1A Pharma[Title/Abstract])) OR (Gemfibrozilo Ur[Title/Abstract])) OR (Gen Gemfibrozil[Title/Abstract])) OR (GenRX Gemfibrozil[Title/Abstract])) OR (Gemfibrozil, GenRX[Title/Abstract])) OR (Healthsense Gemfibrozil[Title/Abstract])) OR (Gemfibrozil, Healthsense[Title/Abstract])) OR (Jezil[Title/Abstract])) OR (Lipazil[Title/Abstract])) OR (Lipox Gemfi[Title/Abstract])) OR (Litarek[Title/Abstract])) OR (Novo Gemfibrozil[Title/Abstract])) OR (Nu Gemfibrozil[Title/Abstract])) OR (Pilder[Title/Abstract])) OR (PMS Gemfibrozil[Title/Abstract])) OR (SBPA Gemfibrozil[Title/Abstract])) OR (Gemfibrozil, SBPA[Title/Abstract])) OR (Terry White Chemists Gemfibrozil[Title/Abstract])) OR (Trialmin[Title/Abstract])) 2,419

#37. "Bezafibrate"[Mesh] 1,317

#38. (((((((((((Bezafibrate[Title/Abstract]) OR (Bezalip[Title/Abstract])) OR (Cedur[Title/Abstract])) OR (BM-15.075[Title/Abstract])) OR (BM 15.075[Title/Abstract])) OR (Lipox[Title/Abstract])) OR (Beza Lande[Title/Abstract])) OR (Beza Puren[Title/Abstract])) OR (Bezafibrat PB[Title/Abstract])) OR (Regadrin B[Title/Abstract])) OR (Be?fizal[Title/Abstract])) OR (Eulitop[Title/Abstract]) 1,880

#39. ("Bezafibrate"[Mesh]) OR ((((((((((((Bezafibrate[Title/Abstract]) OR (Bezalip[Title/Abstract])) OR (Cedur[Title/Abstract])) OR (BM-15.075[Title/Abstract])) OR (BM 15.075[Title/Abstract])) OR (Lipox[Title/Abstract])) OR (Beza Lande[Title/Abstract])) OR (Beza Puren[Title/Abstract])) OR (Bezafibrat PB[Title/Abstract])) OR (Regadrin B[Title/Abstract])) OR (Be?fizal[Title/Abstract])) OR (Eulitop[Title/Abstract])) 2,097

#40. "Ezetimibe"[Mesh] 2,819

#41. ((((((((Ezetimibe[Title/Abstract]) OR ((1-(4-fluorophenyl[Title/Abstract])-(3R)-(3-(4-fluorophenyl)-(3S)-hydroxypropyl)-(4S)-(4-hydroxyphenyl)-2-azetidinone))) OR (Ezetimib[Title/Abstract])) OR (SCH 58235[Title/Abstract])) OR (58235, SCH[Title/Abstract])) OR (SCH-58235[Title/Abstract])) OR (SCH58235[Title/Abstract])) OR (Zetia[Title/Abstract])) OR (Ezetrol[Title/Abstract]) 4,762

#42. ("Ezetimibe"[Mesh]) OR (((((((((Ezetimibe[Title/Abstract]) OR ((1-(4-fluorophenyl[Title/Abstract])-(3R)-(3-(4-fluorophenyl)-(3S)-hydroxypropyl)-(4S)-(4-hydroxyphenyl)-2-azetidinone))) OR (Ezetimib[Title/Abstract])) OR (SCH 58235[Title/Abstract])) OR (58235, SCH[Title/Abstract])) OR (SCH-58235[Title/Abstract])) OR (SCH58235[Title/Abstract])) OR (Zetia[Title/Abstract])) OR (Ezetrol[Title/Abstract])) 5,141

#43. "Diabetic Nephropathies"[Mesh] 32,577

#44. (((((((((((((((((Diabetic Nephropathies[Title/Abstract]) OR (Nephropathies, Diabetic[Title/Abstract])) OR (Nephropathy, Diabetic[Title/Abstract])) OR (Diabetic Kidney Disease[Title/Abstract])) OR (Diabetic Kidney Diseases[Title/Abstract])) OR (Kidney Disease, Diabetic[Title/Abstract])) OR (Kidney Diseases, Diabetic[Title/Abstract])) OR (Diabetic Nephropathy[Title/Abstract])) OR (Diabetic Glomerulosclerosis[Title/Abstract])) OR (Glomerulosclerosis, Diabetic[Title/Abstract])) OR (Intracapillary Glomerulosclerosis[Title/Abstract])) OR (Kimmelstiel-Wilson Disease[Title/Abstract])) OR (Kimmelstiel Wilson Disease[Title/Abstract])) OR (Nodular Glomerulosclerosis[Title/Abstract])) OR (Glomerulosclerosis, Nodular[Title/Abstract])) OR (Kimmelstiel-Wilson Syndrome[Title/Abstract])) OR (Kimmelstiel Wilson Syndrome[Title/Abstract])) OR (Syndrome, Kimmelstiel-Wilson[Title/Abstract]) 32,397

#45. ("Diabetic Nephropathies"[Mesh]) OR ((((((((((((((((((Diabetic Nephropathies[Title/Abstract]) OR (Nephropathies, Diabetic[Title/Abstract])) OR (Nephropathy, Diabetic[Title/Abstract])) OR (Diabetic Kidney Disease[Title/Abstract])) OR (Diabetic Kidney Diseases[Title/Abstract])) OR (Kidney Disease, Diabetic[Title/Abstract])) OR (Kidney Diseases, Diabetic[Title/Abstract])) OR (Diabetic Nephropathy[Title/Abstract])) OR (Diabetic Glomerulosclerosis[Title/Abstract])) OR (Glomerulosclerosis, Diabetic[Title/Abstract])) OR (Intracapillary Glomerulosclerosis[Title/Abstract])) OR (Kimmelstiel-Wilson Disease[Title/Abstract])) OR (Kimmelstiel Wilson Disease[Title/Abstract])) OR (Nodular Glomerulosclerosis[Title/Abstract])) OR (Glomerulosclerosis, Nodular[Title/Abstract])) OR (Kimmelstiel-Wilson Syndrome[Title/Abstract])) OR (Kimmelstiel Wilson Syndrome[Title/Abstract])) OR (Syndrome, Kimmelstiel-Wilson[Title/Abstract])) 45,149

#46. ((((((((((((((("Hypolipidemic Agents"[Mesh]) OR ((((((((((((((((Hypolipidemic Agents[Title/Abstract]) OR (Agents, Hypolipidemic[Title/Abstract])) OR (Antihyperlipidemic[Title/Abstract])) OR (Antilipemic Drug*[Title/Abstract])) OR (Drug*, Antilipemic[Title/Abstract])) OR (Antihyperlipemics[Title/Abstract])) OR (Antihyperlipidemics[Title/Abstract])) OR (Antilipemic Agents[Title/Abstract])) OR (Agents, Antilipemic[Title/Abstract])) OR (Hypolipidemic Drugs[Title/Abstract])) OR (Drugs, Hypolipidemic[Title/Abstract])) OR (Antilipemic*[Title/Abstract])) OR (Hypolipidemic Agent[Title/Abstract])) OR (Agent, Hypolipidemic[Title/Abstract])) OR (Hypolipidemic Drug[Title/Abstract])) OR (Drug, Hypolipidemic[Title/Abstract]))) OR (("Hydroxymethylglutaryl-CoA Reductase Inhibitors"[Mesh]) OR ((((((((((((((((((((((((Hydroxymethylglutaryl-CoA Reductase Inhibitors[Title/Abstract]) OR (Hydroxymethylglutaryl CoA Reductase Inhibitors[Title/Abstract])) OR (Inhibitors, Hydroxymethylglutaryl-CoA Reductase[Title/Abstract])) OR (Reductase Inhibitors, Hydroxymethylglutaryl-CoA[Title/Abstract])) OR (HMG-CoA Reductase Inhibitor[Title/Abstract])) OR (HMG CoA Reductase Inhibitor[Title/Abstract])) OR (Statin*[Title/Abstract])) OR (Inhibitors, HMG-CoA Reductase[Title/Abstract])) OR (Inhibitors, HMG CoA Reductase[Title/Abstract])) OR (Reductase Inhibitors, HMG-CoA[Title/Abstract])) OR (HMG-CoA Reductase Inhibitors[Title/Abstract])) OR (HMG CoA Reductase Inhibitors[Title/Abstract])) OR (Inhibitors, Hydroxymethylglutaryl-Coenzyme A[Title/Abstract])) OR (Hydroxymethylglutaryl-Coenzyme A Inhibitors[Title/Abstract])) OR (Inhibitors, Hydroxymethylglutaryl Coenzyme A[Title/Abstract])) OR (Inhibitors, Hydroxymethylglutaryl-CoA[Title/Abstract])) OR (Hydroxymethylglutaryl-CoA Inhibitors[Title/Abstract])) OR (Inhibitors, Hydroxymethylglutaryl CoA[Title/Abstract])) OR (Hydroxymethylglutaryl-CoA Reductase Inhibitor[Title/Abstract])) OR (Hydroxymethylglutaryl CoA Reductase Inhibitor[Title/Abstract])) OR (Reductase Inhibitor, Hydroxymethylglutaryl-CoA[Title/Abstract])) OR (Statins, HMG-CoA[Title/Abstract])) OR (HMG-CoA Statins[Title/Abstract])) OR (Statins, HMG CoA[Title/Abstract])))) OR (("Atorvastatin"[Mesh]) OR ((((((((((((Atorvastatin[Title/Abstract]) OR ((3R,5R)-7-(2-(4-Fluorophenyl)-5-isopropyl-3-phenyl-4-(phenylcarbamoyl)-1H-pyrrol-1-yl)-3,5-dihydroxyheptanoic acid[Title/Abstract])) OR (Lipitor[Title/Abstract])) OR (Atorvastatin Calcium[Title/Abstract])) OR (Atorvastatin, Calcium Salt[Title/Abstract])) OR (Atorvastatin Calcium Anhydrous[Title/Abstract])) OR (Liptonorm[Title/Abstract])) OR (Atorvastatin Calcium Hydrate[Title/Abstract])) OR (CI 981[Title/Abstract])) OR (CI-981[Title/Abstract])) OR (CI981[Title/Abstract])) OR (Atorvastatin Calcium Trihydrate[Title/Abstract])))) OR (("Simvastatin"[Mesh]) OR ((((((Simvastatin[Title/Abstract]) OR (Zocor[Title/Abstract])) OR (MK-733[Title/Abstract])) OR (MK733[Title/Abstract])) OR (MK 733[Title/Abstract])) OR (Synvinolin[Title/Abstract])))) OR (("Rosuvastatin Calcium"[Mesh]) OR ((((((Rosuvastatin Calcium[Title/Abstract]) OR (Calcium, Rosuvastatin[Title/Abstract])) OR (Rosuvastatin[Title/Abstract])) OR (ZD4522[Title/Abstract])) OR (ZD 4522[Title/Abstract])) OR (Crestor[Title/Abstract])))) OR (("Lovastatin"[Mesh]) OR ((((((((((((Lovastatin[Title/Abstract]) OR (6 Methylcompactin[Title/Abstract])) OR (Mevinolin[Title/Abstract])) OR (Monacolin K[Title/Abstract])) OR (Lovastatin, 1 alpha-Isomer[Title/Abstract])) OR (1 alpha-Isomer Lovastatin[Title/Abstract])) OR (alpha-Isomer Lovastatin, 1[Title/Abstract])) OR (Lovastatin, 1 alpha Isomer[Title/Abstract])) OR (MK-803[Title/Abstract])) OR (MK803[Title/Abstract])) OR (MK 803[Title/Abstract])) OR (Mevacor[Title/Abstract])))) OR (("Pravastatin"[Mesh]) OR (((((((((((((((((((((((((((((Pravastatin[Title/Abstract]) OR (Eptastatin[Title/Abstract])) OR (SQ-31000[Title/Abstract])) OR (SQ 31000[Title/Abstract])) OR (SQ-31,000[Title/Abstract])) OR (SQ 31,000[Title/Abstract])) OR (SQ31,000[Title/Abstract])) OR (Pravastatin tert-Octylamine Salt[Title/Abstract])) OR (Pravastatin tert Octylamine Salt[Title/Abstract])) OR (Pravastatin Sodium[Title/Abstract])) OR (Pravastatin Sodium Salt[Title/Abstract])) OR (Sodium Salt, Pravastatin[Title/Abstract])) OR (Pravastatin, (6 beta)-Isomer[Title/Abstract])) OR (Pravastatin Monosodium Salt, (6 beta)-Isomer[Title/Abstract])) OR (CS-514[Title/Abstract])) OR (CS514[Title/Abstract])) OR (CS 514[Title/Abstract])) OR (Pravachol[Title/Abstract])) OR (Pravacol[Title/Abstract])) OR (Lipostat[Title/Abstract])) OR (Elisor[Title/Abstract])) OR (RMS-431[Title/Abstract])) OR (RMS431[Title/Abstract])) OR (RMS 431[Title/Abstract])) OR (Apo Pravastatin[Title/Abstract])) OR (Mevalotin[Title/Abstract])) OR (Lin Pravastatin[Title/Abstract])) OR (Vasten[Title/Abstract])) OR (Nu Pravastatin[Title/Abstract])))) OR (("Fluvastatin"[Mesh]) OR (((((((((((Fluvastatin[Title/Abstract]) OR (Lescol[Title/Abstract])) OR (XU 62-320[Title/Abstract])) OR (XU 62 320[Title/Abstract])) OR (XU 62320[Title/Abstract])) OR (XU-62320[Title/Abstract])) OR (XU62320[Title/Abstract])) OR (Fluvastatin Sodium[Title/Abstract])) OR (Fluindostatin[Title/Abstract])) OR (Fluvastatin Sodium Salt[Title/Abstract])) OR (7-(3-(4-Fluorophenyl)-1-(1-methylethyl)-1H-indol-2-yl)-3,5-dihydroxy-6-heptenoate[Title/Abstract])))) OR (("pitavastatin" [Supplementary Concept]) OR (((((((((((pitavastatin[Title/Abstract]) OR ((E,3R,5S)-7-(2-cyclopropyl-4-(4-fluorophenyl)quinolin-3-yl)-3,5-dihydroxyhept-6-enoic acid[Title/Abstract])) OR (itavastatin[Title/Abstract])) OR (NK 104[Title/Abstract])) OR (NK-104[Title/Abstract])) OR (P 872441[Title/Abstract])) OR (P-872441[Title/Abstract])) OR (pitavastatin lactone[Title/Abstract])) OR (nisvastatin[Title/Abstract])) OR (pitavastatin calcium[Title/Abstract])) OR (itavastatin calcium[Title/Abstract])))) OR (("Fibric Acids"[Mesh]) OR ((((((((((Fibric Acids[Title/Abstract]) OR (2-Phenoxy Isobutyric Acids[Title/Abstract])) OR (2 Phenoxy Isobutyric Acids[Title/Abstract])) OR (Isobutyric Acids, 2-Phenoxy[Title/Abstract])) OR (2 Phenoxy 2 Methylpropionic Acid Derivatives[Title/Abstract])) OR (Fibric Acid Derivatives[Title/Abstract])) OR (Acid Derivatives, Fibric[Title/Abstract])) OR (Methyl 2 Phenoxypropanoic Acid Derivatives[Title/Abstract])) OR (2-Phenoxy-2-Methylpropionic Acid Derivatives[Title/Abstract])) OR (Fibrate*[Title/Abstract])))) OR (("Fenofibrate"[Mesh]) OR (((((((((((((((((((((((((((((((((((((((((((((Fenofibrate[Title/Abstract]) OR (Procetofen[Title/Abstract])) OR (Phenofibrate[Title/Abstract])) OR (Procetofene[Title/Abstract])) OR (Lipanthyl[Title/Abstract])) OR (Fe?nofibrate Debat[Title/Abstract])) OR (Debat, Fe?nofibrate[Title/Abstract])) OR (Lipantil[Title/Abstract])) OR (Lipidil-Ter[Title/Abstract])) OR (Lipidil Ter[Title/Abstract])) OR (Lipidil[Title/Abstract])) OR (Secalip[Title/Abstract])) OR (Fenofibrat FPh[Title/Abstract])) OR (LF-178[Title/Abstract])) OR (LF178[Title/Abstract])) OR (LF 178[Title/Abstract])) OR (Apo Feno Micro[Title/Abstract])) OR (Apo-Fenofibrate[Title/Abstract])) OR (Apo Fenofibrate[Title/Abstract])) OR (CiL[Title/Abstract])) OR (Controlip[Title/Abstract])) OR (Fe?nofibrate MSD[Title/Abstract])) OR (Gen Fenofibrate[Title/Abstract])) OR (Livesan[Title/Abstract])) OR (MTW Fenofibrat[Title/Abstract])) OR (Tricor[Title/Abstract])) OR (Normalip[Title/Abstract])) OR (Novo Fenofibrate[Title/Abstract])) OR (Nu Fenofibrate[Title/Abstract])) OR (PMS Fenofibrate Micro[Title/Abstract])) OR (Fenofibrat AbZ[Title/Abstract])) OR (Fenofibrat AL[Title/Abstract])) OR (Fenofibrat AZU[Title/Abstract])) OR (AZU, Fenofibrat[Title/Abstract])) OR (Fenofibrat Heumann[Title/Abstract])) OR (Heumann, Fenofibrat[Title/Abstract])) OR (Fenofibrat Hexal[Title/Abstract])) OR (Hexal, Fenofibrat[Title/Abstract])) OR (Fenofibrat Stada[Title/Abstract])) OR (Stada, Fenofibrat[Title/Abstract])) OR (fenofibrat von ct[Title/Abstract])) OR (Fenofibrat ratiopharm[Title/Abstract])) OR (Antara Micronized Procetofen[Title/Abstract])) OR (Micronized Procetofen, Antara[Title/Abstract])) OR (Procetofen, Antara Micronized[Title/Abstract])))) OR (("Gemfibrozil"[Mesh]) OR ((((((((((((((((((((((((((((((((((Gemfibrozil[Title/Abstract]) OR (Gemfibrosil[Title/Abstract])) OR (Lopid[Title/Abstract])) OR (Lopid R[Title/Abstract])) OR (Lipur[Title/Abstract])) OR (CI-719[Title/Abstract])) OR (CI719[Title/Abstract])) OR (CI 719[Title/Abstract])) OR (Apo-Gemfibrozil[Title/Abstract])) OR (Apo Gemfibrozil[Title/Abstract])) OR (Ausgem[Title/Abstract])) OR (Bolutol[Title/Abstract])) OR (Chem mart Gemfibrozil[Title/Abstract])) OR (DBL Gemfibrozil[Title/Abstract])) OR (Decrelip[Title/Abstract])) OR (Gemfi 1A Pharma[Title/Abstract])) OR (Gemfibrozilo Ur[Title/Abstract])) OR (Gen Gemfibrozil[Title/Abstract])) OR (GenRX Gemfibrozil[Title/Abstract])) OR (Gemfibrozil, GenRX[Title/Abstract])) OR (Healthsense Gemfibrozil[Title/Abstract])) OR (Gemfibrozil, Healthsense[Title/Abstract])) OR (Jezil[Title/Abstract])) OR (Lipazil[Title/Abstract])) OR (Lipox Gemfi[Title/Abstract])) OR (Litarek[Title/Abstract])) OR (Novo Gemfibrozil[Title/Abstract])) OR (Nu Gemfibrozil[Title/Abstract])) OR (Pilder[Title/Abstract])) OR (PMS Gemfibrozil[Title/Abstract])) OR (SBPA Gemfibrozil[Title/Abstract])) OR (Gemfibrozil, SBPA[Title/Abstract])) OR (Terry White Chemists Gemfibrozil[Title/Abstract])) OR (Trialmin[Title/Abstract])))) OR (("Bezafibrate"[Mesh]) OR ((((((((((((Bezafibrate[Title/Abstract]) OR (Bezalip[Title/Abstract])) OR (Cedur[Title/Abstract])) OR (BM-15.075[Title/Abstract])) OR (BM 15.075[Title/Abstract])) OR (Lipox[Title/Abstract])) OR (Beza Lande[Title/Abstract])) OR (Beza Puren[Title/Abstract])) OR (Bezafibrat PB[Title/Abstract])) OR (Regadrin B[Title/Abstract])) OR (Be?fizal[Title/Abstract])) OR (Eulitop[Title/Abstract])))) OR (("Ezetimibe"[Mesh]) OR (((((((((Ezetimibe[Title/Abstract]) OR ((1-(4-fluorophenyl[Title/Abstract])-(3R)-(3-(4-fluorophenyl)-(3S)-hydroxypropyl)-(4S)-(4-hydroxyphenyl)-2-azetidinone))) OR (Ezetimib[Title/Abstract])) OR (SCH 58235[Title/Abstract])) OR (58235, SCH[Title/Abstract])) OR (SCH-58235[Title/Abstract])) OR (SCH58235[Title/Abstract])) OR (Zetia[Title/Abstract])) OR (Ezetrol[Title/Abstract])))) AND (("Diabetic Nephropathies"[Mesh]) OR ((((((((((((((((((Diabetic Nephropathies[Title/Abstract]) OR (Nephropathies, Diabetic[Title/Abstract])) OR (Nephropathy, Diabetic[Title/Abstract])) OR (Diabetic Kidney Disease[Title/Abstract])) OR (Diabetic Kidney Diseases[Title/Abstract])) OR (Kidney Disease, Diabetic[Title/Abstract])) OR (Kidney Diseases, Diabetic[Title/Abstract])) OR (Diabetic Nephropathy[Title/Abstract])) OR (Diabetic Glomerulosclerosis[Title/Abstract])) OR (Glomerulosclerosis, Diabetic[Title/Abstract])) OR (Intracapillary Glomerulosclerosis[Title/Abstract])) OR (Kimmelstiel-Wilson Disease[Title/Abstract])) OR (Kimmelstiel Wilson Disease[Title/Abstract])) OR (Nodular Glomerulosclerosis[Title/Abstract])) OR (Glomerulosclerosis, Nodular[Title/Abstract])) OR (Kimmelstiel-Wilson Syndrome[Title/Abstract])) OR (Kimmelstiel Wilson Syndrome[Title/Abstract])) OR (Syndrome, Kimmelstiel-Wilson[Title/Abstract]))) 650

1. **Embase**

#1. 'antilipemic agent'/exp 435231

#2. 'anti hyperlipidemic agent':ab,ti OR 'antihyperlipidemic agent':ab,ti OR 'antihyperlipidemic drug':ab,ti OR antihyperlipidemics:ab,ti OR 'antihypertriglyceridic agent':ab,ti OR 'antihypertriglyceridic drug':ab,ti OR 'antilipaemia agent*':ab,ti OR 'antilipaemic drug':ab,ti OR 'antilipemia agent':ab,ti OR 'antilipemia drug':ab,ti OR 'antilipemic agents':ab,ti OR 'antilipemic drug':ab,ti OR 'antilipidemic agent':ab,ti OR 'antilipidemic drug':ab,ti OR 'hypolipaemic agent':ab,ti OR 'hypolipemic agent':ab,ti OR 'hypolipidemic agent':ab,ti OR 'hypolipidemic agents':ab,ti OR 'hypolipidemic drug':ab,ti OR 'hypotriglyceridic agent':ab,ti OR 'hypotriglyceridic drug':ab,ti OR 'lipid depressing agent':ab,ti OR 'lipid depressing drug':ab,ti OR 'lipid lowering agent':ab,ti OR 'lipid lowering drug':ab,ti OR 'antilipemic agent':ab,ti 4870

#3. #1 OR #2 435788

#4. 'hydroxymethylglutaryl coenzyme a reductase inhibitor'/exp 219269

#5. 'hmg coa reductase inhibitor':ab,ti OR 'hmg coa reductase inhibitors':ab,ti OR 'hmg coenzyme a reductase inhibitor':ab,ti OR 'hmg-coa reductase inhibitors':ab,ti OR 'hydroxymethylglutaryl coa reductase inhibitors':ab,ti OR 'hydroxymethylglutaryl-coa reductase inhibitors':ab,ti OR (statin:ab,ti AND drug:ab,ti) OR statins:ab,ti OR 'hydroxymethylglutaryl coenzyme a reductase inhibitor':ab,ti 66869

#6. #4 OR #5 225482

#7. 'atorvastatin'/exp 52970

#8. atorvastatin:ab,ti OR (2:ab,ti AND '4 fluorophenyl':ab,ti AND 'beta, delta dihydroxy 5':ab,ti AND '2 propanyl':ab,ti AND '3 phenyl 4 phenylaminocarbonyl 1h pyrrole 1 heptanoic acid':ab,ti) OR (2:ab,ti AND '4 fluorophenyl':ab,ti AND 'beta, delta dihydroxy 5 isopropyl 3 phenyl 4 phenylcarbamoyl 1h pyrrole 1 heptanoic acid':ab,ti) OR ('7 [2':ab,ti AND '4 fluorophenyl':ab,ti AND '3 phenyl 4':ab,ti AND phenylaminocarbonyl:ab,ti AND '5 isopropyl 1 pyrrolyl] 3,5 dihydroxyheptanoic acid':ab,ti) OR ('7 [2':ab,ti AND '4 fluorophenyl':ab,ti AND '3 phenyl 4':ab,ti AND phenylcarbamoyl:ab,ti AND 5:ab,ti AND '2 propanyl':ab,ti AND '1 pyrrolyl] 3,5 dihydroxyheptanoic acid':ab,ti) OR ('7 [2':ab,ti AND '4 fluorophenyl':ab,ti AND '3 phenyl 4':ab,ti AND phenylcarbamoyl:ab,ti AND '5 propan 2 ylpyrrol 1 yl] 3, 5 dihydroxyheptanoic acid':ab,ti) OR a2581175:ab,ti OR amicor:ab,ti OR antorcin:ab,ti OR 'apo atorva':ab,ti OR arkas:ab,ti OR artas:ab,ti OR ascord:ab,ti OR astator:ab,ti OR atofast:ab,ti OR ator:ab,ti OR atorab:ab,ti OR atorin:ab,ti OR atorlip:ab,ti OR atormax:ab,ti OR atorstad:ab,ti OR atorstat:ab,ti OR atorvadivid:ab,ti OR atorvalan:ab,ti OR atorvaliq:ab,ti OR 'atorvastatin calcium':ab,ti OR atorvasterol:ab,ti OR atorvox:ab,ti OR atostat:ab,ti OR atovans:ab,ti OR atovarol:ab,ti OR atractin:ab,ti OR atrox:ab,ti OR calipra:ab,ti OR cardiostyl:ab,ti OR cardyl:ab,ti OR ci981:ab,ti OR decholest:ab,ti OR delipost:ab,ti OR gletor:ab,ti OR lambrinex:ab,ti OR larus:ab,ti OR lipibec:ab,ti OR lipimed:ab,ti OR liprimar:ab,ti OR lowlipen:ab,ti OR nelibat:ab,ti OR obradon:ab,ti OR omegastatin:ab,ti OR orbeos:ab,ti OR orvasta:ab,ti OR pd13429838a:ab,ti OR prevencor:ab,ti OR rafitin:ab,ti OR rotacor:ab,ti OR rotova:ab,ti OR s05153:ab,ti OR statorva:ab,ti OR tahor:ab,ti OR torvacard:ab,ti OR 'torvacard neo':ab,ti OR torvas:ab,ti OR vastat:ab,ti OR (vastatin:ab,ti AND atorvastatin:ab,ti) OR vastazor:ab,ti OR xarator:ab,ti OR ym548:ab,ti OR zarator:ab,ti 23100

#9. #7 OR #8 56401

#10. 'simvastatin'/exp 46080

#11. simvastatin:ab,ti OR ('8 [2':ab,ti AND '4 hydroxy 6 oxotetrahydro 2h pyran 2 yl':ab,ti AND 'ethyl] 1, 2, 3, 7, 8, 8a hexahydro 3,7 dimethyl 1 naphthalenyl 2,2 dimethylbutanoate':ab,ti) OR ('8 [2':ab,ti AND '4 hydroxy 6 oxotetrahydro 2h pyran 2 yl':ab,ti AND 'ethyl] 3, 7 dimethyl 1, 2, 3, 7, 8,8a hexahydro 1 naphthalenyl 2,2 dimethylbutanoate':ab,ti) OR ('8 [2':ab,ti AND '4 hydroxy 6 oxotetrahydro 2h pyran 2 yl':ab,ti AND 'ethyl] 3, 7 dimethyl 1, 2, 3,7,8,8a hexahydro 1 naphthalenyl 2,2 dimethylbutanoate':ab,ti) OR alcosin:ab,ti OR 'apo simva':ab,ti OR belmalip:ab,ti OR cholestat:ab,ti OR colastatina:ab,ti OR colemin:ab,ti OR colestricon:ab,ti OR covastin:ab,ti OR denan:ab,ti OR epistatin:ab,ti OR esvat:ab,ti OR eucor:ab,ti OR flolipid:ab,ti OR glipal:ab,ti OR ifistatin:ab,ti OR ipramid:ab,ti OR jabastatina:ab,ti OR kavelor:ab,ti OR kolestevan:ab,ti OR l644128:ab,ti OR labistatin:ab,ti OR lepur:ab,ti OR lipcut:ab,ti OR liporex:ab,ti OR medipo:ab,ti OR mersivas:ab,ti OR mk0733:ab,ti OR nezatin:ab,ti OR nivelipol:ab,ti OR normofat:ab,ti OR nyzoc:ab,ti OR omistat:ab,ti OR pantok:ab,ti OR pravostin:ab,ti OR ranzolont:ab,ti OR rechol:ab,ti OR simar:ab,ti OR simbatrix:ab,ti OR simcovas:ab,ti OR simovil:ab,ti OR simvacor:ab,ti OR simvastatine:ab,ti OR simvor:ab,ti OR sintenal:ab,ti OR sinvalip:ab,ti OR starzoco:ab,ti OR stativer:ab,ti OR vasotenal:ab,ti OR vazim:ab,ti OR velkastatin:ab,ti OR ximve:ab,ti OR xipocol:ab,ti OR zeplan:ab,ti OR zocor:ab,ti OR zorced:ab,ti OR zovar:ab,ti OR zovast:ab,ti 18379

#12. #10 OR #11 47369

#13. 'rosuvastatin'/exp 23177

#14. rosuvastatin:ab,ti OR ('7 [4':ab,ti AND '4 fluorophenyl':ab,ti AND 2:ab,ti AND 'n methylmethanesulfonamido':ab,ti AND 6:ab,ti AND '2 propanyl':ab,ti AND '5 pyrimidinyl] 3,5 dihydroxy 6 heptenoic acid':ab,ti) OR ('7 [4':ab,ti AND '4 fluorophenyl':ab,ti AND 2:ab,ti AND 'n methylmethylsulfonamido':ab,ti AND 6:ab,ti AND '2 propanyl':ab,ti AND '5 pyrimidinyl] 3,5 dihydroxy 6 heptenoic acid':ab,ti) OR ('7 [4':ab,ti AND '4 fluorophenyl':ab,ti AND '2 [':ab,ti AND methanesulfonyl:ab,ti AND methyl:ab,ti AND 'amino] 6':ab,ti AND '1 methylethyl':ab,ti AND '5 pyrimidinyl] 3,5 dihydroxy 6 heptenoic acid':ab,ti) OR ('7 [4':ab,ti AND '4 fluorophenyl':ab,ti AND '2 [':ab,ti AND methanesulfonyl:ab,ti AND methyl:ab,ti AND 'amino] 6 propan 2 ylpyrimidin 5 yl] 3,5 dihydroxyhept 6 enoic acid':ab,ti) OR ('7 [4':ab,ti AND '4 fluorophenyl':ab,ti AND '2 [methyl':ab,ti AND methylsulfonyl:ab,ti AND 'amino] 6 propan 2 ylpyrimidin 5 yl] 3,5 dihydroxyhept 6 enoic acid':ab,ti) OR ('7 [4':ab,ti AND '4 fluorophenyl':ab,ti AND 6:ab,ti AND '1 methylethyl':ab,ti AND 2:ab,ti AND 'n methylmethylsulfonamido':ab,ti AND '5 pyrimidinyl] 3,5 dihydroxy 6 heptenoic acid':ab,ti) OR ('7 [4':ab,ti AND '4 fluorophenyl':ab,ti AND '6 isopropyl 2':ab,ti AND 'n methylmethanesulfonamido':ab,ti AND '5 pyrimidinyl] 3,5 dihydroxy 6 heptenoic acid':ab,ti) OR ('7 [4':ab,ti AND '4 fluorophenyl':ab,ti AND '6 isopropyl 2':ab,ti AND 'n methylmethylsulfonamido':ab,ti AND '5 pyrimidinyl] 3,5 dihydroxy 6 heptenoic acid':ab,ti) OR ('7 [4':ab,ti AND '4 fluorophenyl':ab,ti AND '6 isopropyl 2 [':ab,ti AND methanesulfonyl:ab,ti AND methyl:ab,ti AND 'amino] 5 pyrimidinyl] 3,5 dihydroxy 6 heptenoic acid':ab,ti) OR ('7 [4':ab,ti AND '4 fluorophenyl':ab,ti AND '6 isopropyl 2 [methyl':ab,ti AND methylsulfonyl:ab,ti AND 'amino] 5 pyrimidinyl] 3,5 dihydroxy 6 heptenoic acid':ab,ti) OR (alzil:ab,ti AND rosuvastatin:ab,ti) OR coupet:ab,ti OR creston:ab,ti OR crestor:ab,ti OR crosuvo:ab,ti OR enebium:ab,ti OR epri:ab,ti OR ezallor:ab,ti OR 'ezallor sprinkle':ab,ti OR 'hgp 0816':ab,ti OR hgp0816:ab,ti OR lyn047:ab,ti OR mertenil:ab,ti OR provisacor:ab,ti OR romazic:ab,ti OR ropitor:ab,ti OR ropuido:ab,ti OR rosix:ab,ti OR rosuben:ab,ti OR rosuvador:ab,ti OR rosuvastatina:ab,ti OR roxera:ab,ti OR s4522:ab,ti OR sorvasta:ab,ti OR (visacor:ab,ti AND rosuvastatin:ab,ti) OR 'x plended':ab,ti OR xeter:ab,ti OR zahron:ab,ti OR zaranta:ab,ti OR zd4522:ab,ti OR zelfusor:ab,ti 9815

#15. #13 OR #14 23878

#16. 'mevinolin'/exp 18139

#17. mevinolin:ab,ti OR altocor:ab,ti OR altoprev:ab,ti OR artein:ab,ti OR belvas:ab,ti OR birotin:ab,ti OR cholestra:ab,ti OR cysin:ab,ti OR ellanco:ab,ti OR elstatin:ab,ti OR 'l 654969':ab,ti OR lipdip:ab,ti OR lipivas:ab,ti OR lofacol:ab,ti OR lomar:ab,ti OR lostatin:ab,ti OR lovacel:ab,ti OR lovacol:ab,ti OR lovahexal:ab,ti OR lovalip:ab,ti OR lovalord:ab,ti OR lovastan:ab,ti OR lovastatin:ab,ti OR lovasterol:ab,ti OR lovastin:ab,ti OR lovatadin:ab,ti OR lowachol:ab,ti OR lozutin:ab,ti OR medostatin:ab,ti OR mevacor:ab,ti OR mevinacor:ab,ti OR mk0803:ab,ti OR 'monacolin k':ab,ti OR 'monakolin k':ab,ti OR neolipid:ab,ti OR nergadan:ab,ti OR ovasta:ab,ti OR rovacor:ab,ti OR taucor:ab,ti 6392

#18. #16 OR #17 18841

#19. 'pravastatin'/exp 22781

#20. pravastatin:ab,ti OR ('1, 2, 6, 7, 8, 8a hexahydro beta, delta, 6 trihydroxy 2 methyl 8':ab,ti AND '2 methyl 1 oxobutoxy':ab,ti AND '1 naphthaleneheptanoic acid':ab,ti) OR ('1, 2, 6, 7, 8, 8a hexahydro beta, delta, 6 trihydroxy 2 methyl 8':ab,ti AND '2 methyl 1 oxobutoxy':ab,ti AND '1 naphthaleneheptanoic acid':ab,ti) OR ('3, 5 dihydroxy 7 [1, 2, 6, 7, 8, 8a hexahydro 6 hydroxy 2 methyl 8':ab,ti AND '2 methylbutanoyloxy':ab,ti AND '1 naphthalenyl]heptanoic acid':ab,ti) OR ('3, 5 dihydroxy 7 [6 hydroxy 2 methyl 8 [':ab,ti AND '2 methylbutyryl':ab,ti AND 'oxy] 1, 2, 6, 7, 8, 8a hexahydro 1 naphthalenyl]heptanoic acid':ab,ti) OR ('7 [1, 2, 6, 7, 8, 8a hexahydro 6 hydroxy 2 methyl 8':ab,ti AND '2 methylbutanoyloxy':ab,ti AND '1 naphthalenyl] 3,5 dihydroxyheptanoic acid':ab,ti) OR ('7 [1, 2, 6, 7, 8, 8a hexahydro 6 hydroxy 2 methyl 8':ab,ti AND '2 methylbutyryloxy':ab,ti AND '1 naphthalenyl] 3,5 dihydroxyheptanoic acid':ab,ti) OR ('7 [6 hydroxy 2 methyl 8':ab,ti AND '2 methylbutanoyl':ab,ti AND 'oxy 1, 2, 6, 7, 8, 8a hexahydronaphthalen 1 yl] 3,5 dihydroxyheptanoic acid':ab,ti) OR ('7 [6 hydroxy 2 methyl 8 [':ab,ti AND '2 methylbutyryl':ab,ti AND 'oxy] 1, 2, 6, 7, 8, 8a hexahydro 1 naphthalenyl] 3,5 dihydroxyheptanoic acid':ab,ti) OR aplactin:ab,ti OR astin:ab,ti OR bristacol:ab,ti OR cholespar:ab,ti OR cs514:ab,ti OR 'dehypotin protect':ab,ti OR elisor:ab,ti OR epatostantin:ab,ti OR 'eptastatin sodium':ab,ti OR kenstatin:ab,ti OR lipemol:ab,ti OR liplat:ab,ti OR liprevil:ab,ti OR maxudin:ab,ti OR minuscol:ab,ti OR prareduct:ab,ti OR prascolend:ab,ti OR prava:ab,ti OR pravasin:ab,ti OR 'pravastatin natrium mayrho fer':ab,ti OR pravastatine:ab,ti OR pravator:ab,ti OR pravyl:ab,ti OR sanaprav:ab,ti OR selektine:ab,ti OR sq31000:ab,ti OR stanidine:ab,ti OR vasopran:ab,ti OR vasten:ab,ti OR versatab:ab,ti OR xipral:ab,ti 6775

#21. #19 OR #20 23291

#22. 'fluvastatin'/exp 11180

#23. fluvastatin:ab,ti OR ('7 [3':ab,ti AND '4 fluorophenyl':ab,ti AND 1:ab,ti AND '1 methylethyl':ab,ti AND '1 hydro 2 indolyl] 3, 5 dihydroxy 6 heptenoic acid':ab,ti) OR ('7 [3':ab,ti AND '4 fluorophenyl':ab,ti AND 1:ab,ti AND '2 propanyl':ab,ti AND '1 hydro 2 indolyl] 3, 5 dihydroxy 6 heptenoic acid':ab,ti) OR ('7 [3':ab,ti AND '4 fluorophenyl':ab,ti AND 1:ab,ti AND methylethyl:ab,ti AND '2 indolyl] 3, 5 dihydroxy 6 heptenoic acid':ab,ti) OR ('7 [3':ab,ti AND '4 fluorophenyl':ab,ti AND 1:ab,ti AND 'propan 2 yl':ab,ti AND 'indol 2 yl] 3, 5 dihydroxyhept 6 enoic acid':ab,ti) OR ('7 [3':ab,ti AND '4 fluorophenyl':ab,ti AND '1 isopropyl 1 hydroindol 2 yl] 3, 5 dihydroxyhept 6 enoic acid':ab,ti) OR ('7 [3':ab,ti AND '4 fluorophenyl':ab,ti AND '1 isopropyl 2 indolyl] 3, 5 dihydroxy 6 heptenoic acid':ab,ti) OR ('7 [3':ab,ti AND '4 fluorophenyl':ab,ti AND '1 propan 2 yl 1h indol 2 yl] 3, 5 dihydroxyhept 6 enoic acid':ab,ti) OR almastatin:ab,ti OR canef:ab,ti OR (cardiol:ab,ti AND fluindostatin:ab,ti) OR 'cardiol xl':ab,ti OR cranoc:ab,ti OR digardil:ab,ti OR digaril:ab,ti OR fluindostatin:ab,ti OR 'fluvastatin sodium':ab,ti OR fluvastatina:ab,ti OR fluvastatine:ab,ti OR 'fractal lp':ab,ti OR 'leposit prolib':ab,ti OR 'lescol exel':ab,ti OR 'lescol mr':ab,ti OR leucol:ab,ti OR (lipaxin:ab,ti AND fluvastatin:ab,ti) OR 'liposit prolib':ab,ti OR lochol:ab,ti OR lymetel:ab,ti OR primexin:ab,ti OR 'sri 62320':ab,ti OR sri62320:ab,ti OR vaditon:ab,ti OR (vastin:ab,ti AND fluindostatin:ab,ti) OR 'xu 62320':ab,ti OR xu62320:ab,ti 3115

#24. #22 OR #23 11331

#25. 'pitavastatin'/exp 4939

#26. pitavastatin:ab,ti OR ('7 [2 cyclopropyl 4':ab,ti AND '4 fluorophenyl':ab,ti AND '3 quinolyl] 3, 5 dihydroxy 6 heptenoic acid':ab,ti) OR ('7 [2 cyclopropyl 4':ab,ti AND '4 fluorophenyl':ab,ti AND 'quinolin 3 yl] 3, 5 dihydroxy 6 heptanoic acid':ab,ti) OR ('7 [2 cyclopropyl 4':ab,ti AND '4 fluorophenyl':ab,ti AND 'quinolin 3 yl] 3, 5 dihydroxy 6 heptenoic acid':ab,ti) OR ('7 [2 cyclopropyl 4':ab,ti AND '4 fluorophenyl':ab,ti AND 'quinolin 3 yl] 3, 5 dihydroxyhept 6 enoic acid':ab,ti) OR ('7 [2 cyclopropyl 4':ab,ti AND '4 fluorophenyl':ab,ti AND 'quinolin 3 yl] 3, 5 dihydroxyheptan 6 oic acid':ab,ti) OR alipza:ab,ti OR itavastatin:ab,ti OR 'itavastatin calcium':ab,ti OR lippiza:ab,ti OR livalo:ab,ti OR livazo:ab,ti OR ('monocalcium bis [7 [2 cyclopropyl 4':ab,ti AND '4 fluorophenyl':ab,ti AND '3 quinolyl] 3, 5 dihydroxy 6 heptenoate':ab,ti) OR nikita:ab,ti OR nisvastatin:ab,ti OR 'nk 104':ab,ti OR nk104:ab,ti OR 'nks 104':ab,ti OR nks104:ab,ti OR 'p 872441':ab,ti OR p872441:ab,ti OR pitava:ab,ti OR 'pitavastatin calcium':ab,ti OR 'pitavastatin magnesium':ab,ti OR 'pitavastatin sodium':ab,ti OR redevant:ab,ti OR ribar:ab,ti OR trolise:ab,ti OR vezepra:ab,ti OR zypitamag:ab,ti 2302

#27. #25 OR #26 5131

#28. 'fibric acid derivative'/exp 42329

#29. 'fibric acid derivative':ab,ti OR fibrate:ab,ti OR 'fibrate derivative':ab,ti OR fibrates:ab,ti OR 'fibric acid':ab,ti OR 'fibric acids':ab,ti 6002

#30. #28 OR #29 43417

#31. 'fenofibrate'/exp 14002

#32. fenofibrate:ab,ti OR ('1 methylethyl':ab,ti AND '2 [4':ab,ti AND '4 chlorobenzoyl':ab,ti AND 'phenoxy] 2 methylpropanoate':ab,ti) OR ('2 propanyl':ab,ti AND '2 [4':ab,ti AND '4 chlorobenzoyl':ab,ti AND 'phenoxy] 2 methylpropanoate':ab,ti) OR ('2 propanyl':ab,ti AND '[4 [':ab,ti AND '4 chlorophenyl':ab,ti AND 'carbonyl] 2 phenoxy 2 methyl] propionate':ab,ti) OR ('2 propyl':ab,ti AND '[4 [':ab,ti AND '4 chlorophenyl':ab,ti AND 'carbonyl] 2 phenoxy 2 methyl] propanoate':ab,ti) OR ('2 [4':ab,ti AND '4 chlorobenzoyl':ab,ti AND 'phenoxy] 2 methylpropionic acid 2 propanyl ester':ab,ti) OR ('2 [4 [':ab,ti AND '4 chlorophenyl':ab,ti AND 'carbonyl] phenoxy] 2 methylpropanoic acid propan 2 yl ester':ab,ti) OR ('2 [4 [':ab,ti AND '4 chlorophenyl':ab,ti AND 'carbonyl] phenoxy] 2 methylpropionic acid propan 2 yl ester':ab,ti) OR antara:ab,ti OR (antara:ab,ti AND micronized:ab,ti) OR apteor:ab,ti OR biofibrat:ab,ti OR catalip:ab,ti OR climage:ab,ti OR durafenat:ab,ti OR elipsia:ab,ti OR evothyl:ab,ti OR fegenor:ab,ti OR felosma:ab,ti OR fenochol:ab,ti OR (fenofibrate:ab,ti AND micronized:ab,ti) OR fenogal:ab,ti OR 'fenogal lidose':ab,ti OR fenomax:ab,ti OR fenox:ab,ti OR grs001:ab,ti OR hyperchol:ab,ti OR ('isopropyl 2 [4':ab,ti AND '4 chlorobenzoyl':ab,ti AND 'phenoxy] 2 methylpropanoate':ab,ti) OR ('isopropyl [4':ab,ti AND '4 chlorobenzoyl':ab,ti AND '2 phenoxy 2 methyl] propanoate':ab,ti) OR ('isopropyl [4 [':ab,ti AND '4 chlorophenyl':ab,ti AND 'carbonyl] 2 phenoxy 2 methyl] propionate':ab,ti) OR katalip:ab,ti OR lf178:ab,ti OR 'lifenoz u':ab,ti OR lipanthyl:ab,ti OR 'lipanthyl penta':ab,ti OR 'lipanthyl supra':ab,ti OR 'lipantil nano':ab,ti OR lipantyl:ab,ti OR 'lipidil ter':ab,ti OR lipoclar:ab,ti OR 'nopid 200':ab,ti OR nubrex:ab,ti OR procetofen:ab,ti OR procetofene:ab,ti OR ('propan 2 yl 2 [4 [':ab,ti AND '4 chlorophenyl':ab,ti AND 'carbonyl] phenoxy] 2 methylpropanoate':ab,ti) OR ('propan 2 yl [4':ab,ti AND '4 chlorobenzoyl':ab,ti AND '2 phenoxy 2 methyl] propionate':ab,ti) OR ('propan 2 yl [4 [':ab,ti AND '4 chlorophenyl':ab,ti AND 'carbonyl] 2 phenoxy 2 methyl] propionate':ab,ti) OR qualipantyl:ab,ti OR rapidil:ab,ti OR rorit:ab,ti OR rp1824:ab,ti OR secalip:ab,ti OR sigurtil:ab,ti OR suprelip:ab,ti OR trichol:ab,ti OR tricor:ab,ti OR triglide:ab,ti OR trolip:ab,ti OR xafenor:ab,ti OR zerlubron:ab,ti OR ziglip:ab,ti OR zigotrig:ab,ti OR zumafib:ab,ti 6498

#33. #31 OR #32 14520

#34. 'gemfibrozil'/exp 9866

#35. gemfibrozil:ab,ti OR ('2, 2 dimethyl 5':ab,ti AND '2, 5 xylyloxy':ab,ti AND 'pentanoic acid':ab,ti) OR ('2, 2 dimethyl 5':ab,ti AND '2, 5 xylyloxy':ab,ti AND 'valeric acid':ab,ti) OR (5:ab,ti AND '2, 5 dimethylphenoxy':ab,ti AND '2, 2 dimethylvaleric acid':ab,ti) OR 'apo gemfibrozil':ab,ti OR ausgem:ab,ti OR bolutol:ab,ti OR brozil:ab,ti OR chlorestrol:ab,ti OR ci719:ab,ti OR clearol:ab,ti OR decrelip:ab,ti OR elmogan:ab,ti OR fibralip:ab,ti OR fibrocit:ab,ti OR gemfibril:ab,ti OR gemlipid:ab,ti OR gemzil:ab,ti OR gevilon:ab,ti OR gozid:ab,ti OR hidil:ab,ti OR hipolixan:ab,ti OR ipolipid:ab,ti OR jezil:ab,ti OR lanaterom:ab,ti OR lipidys:ab,ti OR lipira:ab,ti OR lipostorol:ab,ti OR 'lopid o.d.':ab,ti OR manobrozil:ab,ti OR mersikol:ab,ti OR normolip:ab,ti OR polyxit:ab,ti OR progemzal:ab,ti OR regulip:ab,ti OR triglizil:ab,ti OR uragem:ab,ti OR zilop:ab,ti 2751

#36. #34 OR #35 10105

#37. 'bezafibrate'/exp 6151

#38. bezafibrate:ab,ti OR ('2 [4 [2':ab,ti AND '4 chlorobenzamido':ab,ti AND 'ethyl] phenoxy] 2 methylpropanoic acid':ab,ti) OR ('2 [4 [2':ab,ti AND '4 chlorobenzamido':ab,ti AND 'ethyl] phenoxy] 2 methylpropionic acid':ab,ti) OR befizal:ab,ti OR benzafibrate:ab,ti OR benzofibrate:ab,ti OR 'bezafibrate retard':ab,ti OR bezalip:ab,ti OR 'bezalip mono':ab,ti OR 'bezalip retard':ab,ti OR bezatol:ab,ti OR bezifal:ab,ti OR bezofibrate:ab,ti OR bf759:ab,ti OR 'bm 15075':ab,ti OR bm15075:ab,ti OR calberzol:ab,ti OR 'calberzol xl':ab,ti OR cedur:ab,ti OR 'cedur retard':ab,ti OR lipozate:ab,ti OR lo44:ab,ti OR norlip:ab,ti 2500

#39. #37 OR #38 6360

#40. 'ezetimibe'/exp 16465

#41. ezetimibe:ab,ti OR (1:ab,ti AND '3 [3':ab,ti AND '4 fluorophenyl':ab,ti AND '3 hydroxypropyl] 4':ab,ti AND '4 hydroxyphenyl':ab,ti AND '2 azetidinone':ab,ti) OR absorcol:ab,ti OR adezop:ab,ti OR azibe:ab,ti OR carditimib:ab,ti OR cexado:ab,ti OR cildar:ab,ti OR coltowan:ab,ti OR corintus:ab,ti OR delipid:ab,ti OR egitim:ab,ti OR elanix:ab,ti OR erezel:ab,ti OR esetin:ab,ti OR etibax:ab,ti OR ezdivule:ab,ti OR ezedoc:ab,ti OR ezegelan:ab,ti OR ezegros:ab,ti OR ezehron:ab,ti OR ezelip:ab,ti OR ezetebmib:ab,ti OR ezetiben:ab,ti OR ezgal:ab,ti OR ezicor:ab,ti OR hgp1404:ab,ti OR intestat:ab,ti OR kobey:ab,ti OR lipegis:ab,ti OR lipobon:ab,ti OR mibecol:ab,ti OR mibezet:ab,ti OR noxetib:ab,ti OR olufsent:ab,ti OR quver:ab,ti OR sch58235:ab,ti OR tezzimi:ab,ti OR trizibe:ab,ti OR viemm:ab,ti OR vyeve:ab,ti OR zertya:ab,ti OR zetratak:ab,ti OR zient:ab,ti 8521

#42. #40 OR #41 17616

#43. 'diabetic nephropathy'/exp 65211

#44. 'diabetes nephropathy':ab,ti OR 'diabetic kidney disease':ab,ti OR 'diabetic nephropathies':ab,ti OR 'diabetic renal disease':ab,ti OR 'diabetic nephropathy':ab,ti 45450

#45. #43 OR #44 71943

#46. #3 OR #6 OR #9 OR #12 OR #15 OR #18 OR #21 OR #24 OR #27 OR #30 OR #33 OR #36 OR #39 OR #42 445920

#47. #45 AND #46 3877

1. **Cochrane Library**

#1. MeSH descriptor: [Hypolipidemic Agents] explode all trees 9138

#2. (Hypolipidemic Agent*):ti,ab,kw OR (Antihyperlipemic*):ti,ab,kw OR (Antilipemic Drug*):ti,ab,kw OR (Drug*, Hypolipidemic):ti,ab,kw OR (Agent*, Antilipemic):ti,ab,kw 2415

#3. (Agent*, Hypolipidemic):ti,ab,kw OR (Antilipemic):ti,ab,kw OR (Antilipemic Agent*):ti,ab,kw OR (Hypolipidemic Drug*):ti,ab,kw OR (Drug*, Antilipemic*):ti,ab,kw 2436

#4. #1 OR #2 OR #3 9687

#5. MeSH descriptor: [Hydroxymethylglutaryl-CoA Reductase Inhibitors] explode all trees 5046

#6. (Hydroxymethylglutaryl-CoA Reductase Inhibitors):ti,ab,kw OR (Inhibitors, Hydroxymethylglutaryl-Coenzyme A):ti,ab,kw OR (Inhibitors, HMG-CoA Reductase):ti,ab,kw OR (Statin):ti,ab,kw OR (HMG-CoA Reductase Inhibitors):ti,ab,kw 8571

#7. (Inhibitors, HMG CoA Reductase):ti,ab,kw OR (HMG-CoA Statins):ti,ab,kw OR (Reductase Inhibitor, Hydroxymethylglutaryl-CoA):ti,ab,kw OR (Hydroxymethylglutaryl CoA Reductase Inhibitor):ti,ab,kw OR (Inhibitors, Hydroxymethylglutaryl Coenzyme A):ti,ab,kw 1042

#8. (Reductase Inhibitors, Hydroxymethylglutaryl-CoA):ti,ab,kw OR (HMG-CoA Reductase Inhibitor):ti,ab,kw OR (Inhibitors, Hydroxymethylglutaryl-CoA):ti,ab,kw OR (Statins, HMG CoA):ti,ab,kw OR (Hydroxymethylglutaryl-Coenzyme A Inhibitors):ti,ab,kw 640

#9. (HMG CoA Reductase Inhibitors):ti,ab,kw OR (Statins; Hydroxymethylglutaryl-CoA Inhibitors):ti,ab,kw OR (Hydroxymethylglutaryl CoA Reductase Inhibitors):ti,ab,kw OR (Inhibitors, Hydroxymethylglutaryl CoA; Statins, HMG-CoA):ti,ab,kw OR (Inhibitors, Hydroxymethylglutaryl-CoA Reductase):ti,ab,kw 202

#10. (HMG CoA Reductase Inhibitor):ti,ab,kw OR (Reductase Inhibitors, HMG-CoA):ti,ab,kw OR (Hydroxymethylglutaryl-CoA Reductase Inhibitor):ti,ab,kw 143

#11 . #5 OR #6 OR #7 OR #8 OR #9 OR #10 10827

#12. MeSH descriptor: [Atorvastatin] explode all trees 2951

#13. (Atorvastatin):ti,ab,kw OR (CI 981):ti,ab,kw OR (CI-981):ti,ab,kw OR (CI981):ti,ab,kw OR (Atorvastatin Calcium Trihydrate):ti,ab,kw 6761

#14. (Atorvastatin Calcium Hydrate):ti,ab,kw OR (Liptonorm):ti,ab,kw 2

#15. #12 OR #13 OR #14 6761

#16. MeSH descriptor: [Simvastatin] explode all trees 2169

#17. (Simvastatin):ti,ab,kw OR (MK 733):ti,ab,kw OR (MK733):ti,ab,kw OR (Synvinolin):ti,ab,kw OR (Zocor):ti,ab,kw 4253

#18. #16 OR #17 4253

#19. MeSH descriptor: [Rosuvastatin Calcium] explode all trees 1437

#20. (Rosuvastatin Calcium):ti,ab,kw OR (ZD4522):ti,ab,kw OR (Rosuvastatin):ti,ab,kw OR (Crestor):ti,ab,kw OR (Calcium):ti,ab,kw 36846

#21. #19 OR #20 36846

#22. MeSH descriptor: [Lovastatin] explode all trees 2582

#23. (Lovastatin):ti,ab,kw OR (alpha-Isomer Lovastatin, 1):ti,ab,kw OR (Lovastatin, 1 alpha-Isomer):ti,ab,kw OR (Lovastatin, 1 alpha Isomer):ti,ab,kw OR (1 alpha-Isomer Lovastatin):ti,ab,kw 1013

#24. (Mevacor):ti,ab,kw OR (MK803):ti,ab,kw OR (Monacolin K):ti,ab,kw OR (6 Methylcompactin):ti,ab,kw OR (Mevinolin):ti,ab,kw 164

#25. #21 OR #22 OR #23 39493

#26. MeSH descriptor: [Pravastatin] explode all trees 1224

#27. (Pravastatin Sodium Salt):ti,ab,kw OR (SQ31000):ti,ab,kw OR (Liplat):ti,ab,kw OR (Prareduct):ti,ab,kw OR (Mevalotin):ti,ab,kw 7

#28. (Bristacol):ti,ab,kw OR (Pravastatin tert Octylamine Salt):ti,ab,kw OR (Apo Pravastatin):ti,ab,kw OR (Lipemol):ti,ab,kw OR (Lin Pravastatin):ti,ab,kw 103

#29. (Pravasin):ti,ab,kw OR (Selektine):ti,ab,kw OR (Pravacol):ti,ab,kw OR (Lipostat):ti,ab,kw OR (Pravachol):ti,ab,kw 18

#30. (Elisor):ti,ab,kw OR (CS514):ti,ab,kw OR (Nu Pravastatin):ti,ab,kw OR (Eptastatin):ti,ab,kw 3

#31. #26 OR #27 OR #28 OR #29 OR #30 1275

#32. MeSH descriptor: [Fluvastatin] explode all trees 377

#33. (Fluvastatin):ti,ab,kw OR (XU62320):ti,ab,kw OR (Fluindostatin):ti,ab,kw OR (Fluvastatin Sodium Salt):ti,ab,kw OR (Fluvastatin Sodium):ti,ab,kw 777

#34. #32 OR #33 777

#35. MeSH descriptor: [Fibric Acids] explode all trees 1521

#36. (Fibric Acids):ti,ab,kw OR (Acid Derivatives, Fibric):ti,ab,kw OR (Fibric Acid Derivatives):ti,ab,kw OR (Methyl 2 Phenoxypropanoic Acid Derivatives):ti,ab,kw OR (Fibrate):ti,ab,kw 306

#37. #35 OR #36 1662

#38. MeSH descriptor: [Fenofibrate] explode all trees 687

#39. (Fenofibrate):ti,ab,kw OR (Fenofibrat Stada):ti,ab,kw OR (Stada, Fenofibrat):ti,ab,kw OR (Heumann, Fenofibrat):ti,ab,kw OR (Fenofibrat Heumann):ti,ab,kw 1167

#40. (Gen Fenofibrate):ti,ab,kw OR (Fenofibrat Hexal):ti,ab,kw OR (Lipidil):ti,ab,kw OR (Fenofibrat FPh):ti,ab,kw OR (Secalip):ti,ab,kw 15

#41. (Lipantil):ti,ab,kw OR (Supralip):ti,ab,kw OR (Tricor):ti,ab,kw OR (Apo Fenofibrate):ti,ab,kw OR (Fenofanton):ti,ab,kw 151

#42. (Novo Fenofibrate):ti,ab,kw OR (AZU, Fenofibrat):ti,ab,kw OR (durafenat):ti,ab,kw OR (Antara Micronized Procetofen):ti,ab,kw OR (LF178):ti,ab,kw 3

#43. (PMS Fenofibrate Micro):ti,ab,kw OR (Procetofen):ti,ab,kw OR (Procetofene):ti,ab,kw OR (Phenofibrate):ti,ab,kw OR (Lofibra):ti,ab,kw 22

#44. #38 OR #39 OR #40 OR #41 OR #42 OR #43 1180

#45. MeSH descriptor: [Gemfibrozil] explode all trees 370

#46. (Gemfibrozil):ti,ab,kw OR (Lipox Gemfi):ti,ab,kw OR (Jezil):ti,ab,kw OR (Gemfibrozil, Healthsense):ti,ab,kw OR (Healthsense Gemfibrozil):ti,ab,kw 562

#47. (Gemfi 1A Pharma):ti,ab,kw OR (Lipazil):ti,ab,kw OR (Gemfibrosil):ti,ab,kw OR (Trialmin):ti,ab,kw OR (Chem mart Gemfibrozil):ti,ab,kw 2

#48. (Bayvit, Gemfibrozilo):ti,ab,kw OR (CI719):ti,ab,kw OR (Nu-Gemfibrozil):ti,ab,kw OR (NuGemfibrozil):ti,ab,kw OR (Apo Gemfibrozil):ti,ab,kw 51

#49. #45 OR #46 OR #47 OR #48 562

#50. MeSH descriptor: [Bezafibrate] explode all trees 274

#51. (Bezafibrate):ti,ab,kw OR (Beza-Lande):ti,ab,kw OR (Bezacur):ti,ab,kw OR (Regadrin B):ti,ab,kw OR (Reducterol):ti,ab,kw 487

#52. (Cedur):ti,ab,kw OR (Sklerofibrat):ti,ab,kw OR (Bezalip):ti,ab,kw OR (Azufibrat):ti,ab,kw OR (Lipox):ti,ab,kw 29

#53. (BM 15.075):ti,ab,kw OR (Difaterol):ti,ab,kw OR (Beza Puren):ti,ab,kw OR (Solibay):ti,ab,kw OR (Eulitop):ti,ab,kw 2

#54. #50 OR #51 OR #52 OR #53 499

#55. MeSH descriptor: [Ezetimibe] explode all trees 1025

#56. (Ezetimibe):ti,ab,kw OR (Ezetrol):ti,ab,kw OR (Ezetimib):ti,ab,kw OR (SCH58235):ti,ab,kw OR (Zetia):ti,ab,kw 2122

#57. #55 OR #56 2122

#58. MeSH descriptor: [Diabetic Nephropathies] explode all trees 2046

#59. (Diabetic Nephropathies):ti,ab,kw OR (Syndrome, Kimmelstiel-Wilson):ti,ab,kw OR (Glomerulosclerosis, Nodular):ti,ab,kw OR (Kimmelstiel Wilson Syndrome):ti,ab,kw OR (Intracapillary Glomerulosclerosis):ti,ab,kw 2114

#60. (Kimmelstiel-Wilson Syndrome):ti,ab,kw OR (Kimmelstiel-Wilson Disease):ti,ab,kw OR (Nodular Glomerulosclerosis):ti,ab,kw OR (Kidney Disease*, Diabetic):ti,ab,kw OR (Nephropathies, Diabetic):ti,ab,kw 2114

#61. #58 OR #59 OR #60 2144

#62. #4 OR #11 OR #15 OR #18 OR #21 OR #25 OR #31 OR #34 OR #37 OR #44 OR #49 OR #54 OR #57 55812

#63. #61 and #62 236

1. **Web of Science**

#127."(((((((((((((((((((((((((((((((((((((((((((((((((((((((((((((((((((((((((((((((((((((((((((((((((((((((((((((((((((((((((((((((((((((((((((((((((((((((((((((((((((TS=(Hypolipidemic Agent*)) OR TS=(Antihyperlipidemic*)) OR TS=(Antilipemic Drug*)) OR TS=(Antihyperlipemics)) OR TS=(Antilipemic*)) OR TS=(Hypolipidemic Drug*)) OR TS=(Lipid-lowering drug*)) OR TS=(lipid-lowering agent*)) OR TS=(Hydroxymethylglutaryl-CoA Reductase Inhibitor*)) OR TS=(HMG CoA Reductase Inhibitor*)) OR TS=(Hydroxymethylglutaryl-Coenzyme A Inhibitors)) OR TS=(Hydroxymethylglutaryl-CoA Inhibitors)) OR TS=(HMG-CoA Statins)) OR TS=(Statin*)) OR TS=(Atorvastatin)) OR TS=((3R,5R)-7-(2-(4-Fluorophenyl)-5-isopropyl-3-phenyl-4-(phenylcarbamoyl)-1H-pyrrol-1-yl)-3,5-dihydroxyheptanoic acid)) OR TS=(Lipitor)) OR TS=(Liptonorm)) OR TS=(CI 981)) OR TS=(CI-981)) OR TS=(CI981)) OR TS=(Simvastatin)) OR TS=(Zocor)) OR TS=(MK-733)) OR TS=(MK733)) OR TS=(MK 733)) OR TS=(Synvinolin)) OR TS=(Rosuvastatin)) OR TS=(ZD4522)) OR TS=(ZD 4522)) OR TS=(Crestor)) OR TS=(Lovastatin)) OR TS=(6 Methylcompactin)) OR TS=(Mevinolin)) OR TS=(Monacolin K)) OR TS=(Lovastatin)) OR TS=(MK-803)) OR TS=(MK803)) OR TS=(MK 803)) OR TS=(Mevacor)) OR TS=(Pravastatin)) OR TS=(Eptastatin)) OR TS=(SQ-31000)) OR TS=(SQ 31000)) OR TS=(SQ-31,000)) OR TS=(CS-514)) OR TS=(CS 514)) OR TS=(Pravachol)) OR TS=(Lipostat)) OR TS=(Elisor)) OR TS=(RMS-431)) OR TS=(RMS431)) OR TS=(Fluvastatin)) OR TS=(Lescol)) OR TS=(XU 62-320)) OR TS=(XU 62 320)) OR TS=(pitavastatin)) OR TS=((E,3R,5S)-7-(2-cyclopropyl-4-(4-fluorophenyl)quinolin-3-yl)-3,5-dihydroxyhept-6-enoic acid)) OR TS=(itavastatin)) OR TS=(NK 104)) OR TS=(NK-104)) OR TS=(P 872441)) OR TS=(P-872441)) OR TS=(pitavastatin)) OR TS=(nisvastatin)) OR TS=(Fibric Acid*)) OR TS=(2 Phenoxy Isobutyric Acids)) OR TS=(Isobutyric Acids, 2-Phenoxy)) OR TS=(2 Phenoxy 2 Methylpropionic Acid Derivatives)) OR TS=(Methyl 2 Phenoxypropanoic Acid Derivatives)) OR TS=(Fibrate*)) OR TS=(Fenofibrate)) OR TS=(Procetofen*)) OR TS=(Phenofibrate)) OR TS=(Lipanthyl)) OR TS=(LF-178)) OR TS=(LF178)) OR TS=(Apo Feno Micro)) OR TS=(CiL)) OR TS=(Controlip)) OR TS=(Livesan)) OR TS=(Tricor)) OR TS=(Gemfibrozil)) OR TS=(Gemfibrosil*)) OR TS=(Lopid)) OR TS=(Lipur)) OR TS=(CI-719)) OR TS=(CI719)) OR TS=(Ausgem)) OR TS=(Bolutol)) OR TS=(Decrelip)) OR TS=(Gemfi 1A Pharma)) OR TS=(Jezil)) OR TS=(Lipazil)) OR TS=(Litarek)) OR TS=(Novo Gemfibrozil)) OR TS=(Pilder)) OR TS=(Bezafibrate)) OR TS=(Bezalip)) OR TS=(Cedur)) OR TS=(BM-15.075)) OR TS=(Lipox)) OR TS=(Beza Lande)) OR TS=(Beza Puren)) OR TS=(Bezafibrat)) OR TS=(Regadrin B)) OR TS=(Eulitop)) OR TS=(Ezetimibe)) OR TS=(1-(4-fluorophenyl）)) OR TS=(SCH 58235)) OR TS=(SCH-58235)) OR TS=(SCH58235)) OR TS=(Zetia)) OR TS=(Ezetrol)) and Preprint Citation Index (Exclude – Database)" All Databases [110040]

#128. "((((((((TS=(Diabetic Nephropathies)) OR TS=(Diabetic Kidney Disease*)) OR TS=(Diabetic Nephropathy)) OR TS=(Diabetic Glomerulosclerosis)) OR TS=(Intracapillary Glomerulosclerosis)) OR TS=(Kimmelstiel-Wilson)) OR TS=(Kimmelstiel Wilson)) OR TS=(Nodular Glomerulosclerosis)) OR TS=(Glomerulosclerosis, Nodular) and Preprint Citation Index (Exclude – Database)" All Databases [36089]

#129. "#127 AND #128 and Preprint Citation Index (Exclude – Database)" All Databases [831]

**Tab. S1.** Characteristics of Included Studies

| **Outcome** | **No. of studies** | **Study design** | **Risk of bias** | **Inconsistency**  **(i²)** | **Indirectness** | **Imprecision** | **Publication bias** | **Overall certainty**  **(GRADE)** | **Comments** |
| --- | --- | --- | --- | --- | --- | --- | --- | --- | --- |
| TC | 14 | RCT | Not serious (0) | Serious (-1) | Serious (-1) | Serious (-1) | Not serious (0) | low | Downgraded due to high heterogeneity (I²=87.4%), wide CI crossing the null and indirect evidence. |
| TG | 12 | RCT | Not serious (0) | Serious (-1) | Serious (-1) | Serious (-1) | Not serious (0) | low | Downgraded due to high heterogeneity (I²=52.8%), wide CI crossing the null and indirect evidence. |
| LDL-C | 12 | RCT | Not serious (0) | Serious (-1) | Serious (-1) | Serious (-1) | Not serious (0) | low | Downgraded due to high heterogeneity (I²=81%), wide CI crossing the null and indirect evidence. |
| HDL-C | 13 | RCT | Not serious (0) | Serious (-1) | Serious (-1) | Serious (-1) | Not serious (0) | low | Downgraded due to high heterogeneity (I²=52%), wide CI crossing the null and indirect evidence. |
| eGFR | 12 | RCT | Not serious (0) | Not serious (0) | Serious (-1) | Serious (-1) | Not serious (0) | Moderate | wide CI crossing the null and indirect evidence. |
| 24hUTP | 5 | RCT | Not serious (0) | Serious (-1) | Not serious (0) | Serious (-1) | Not serious (0) | Moderate | Downgraded due to high heterogeneity (I²=61%) and wide CI crossing the null. |
| Scr | 9 | RCT | Not serious (0) | Not serious (0) | Serious (-1) | Serious (-1) | Not serious (0) | Moderate | wide CI crossing the null and indirect evidence. |
| Cardiovascular Events | 3 | RCT | Not serious (0) | Serious (-1) | Not serious (0) | Serious (-1) | Not serious (0) | low | Downgraded due to high heterogeneity (I²=55.2%), wide CI crossing the null and limited number of studies. |
| Mortality Rate | 4 | RCT | Not serious (0) | Serious (-1) | Serious (-1) | Serious (-1) | Not serious (0) | low | Downgraded due to high heterogeneity (I²=48.7%), wide CI crossing the null and Limited number of studies. |
| HbA1c | 11 | RCT | Not serious (0) | serious (-1) | Serious (-1) | Serious (-1) | Not serious (0) | low | Downgraded due to high heterogeneity (I²=49.3%), wide CI crossing the null and indirect evidence. |

**Tab. S1.** Certainty of Evidence for Outcome Measures of Lipid-Lowering Drugs

**
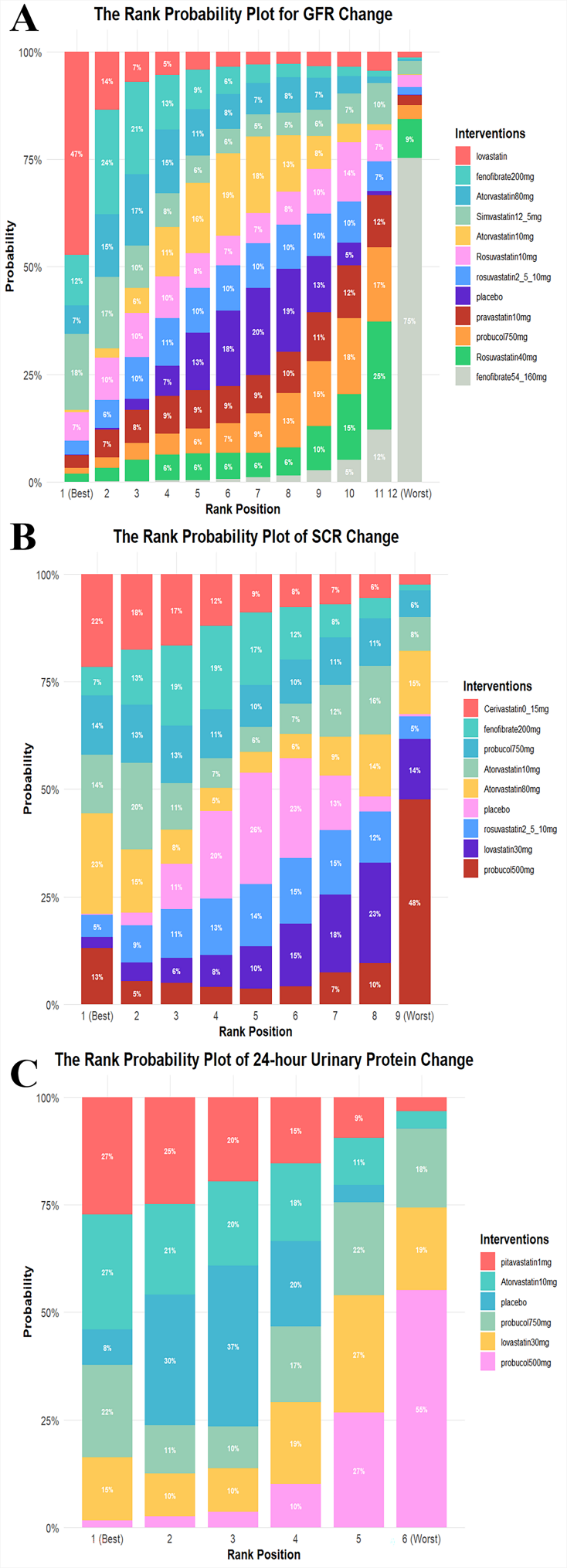
**

**Fig. S1.** The ranking probability plot. A: eGFR; B: SCR; C: UPR

**
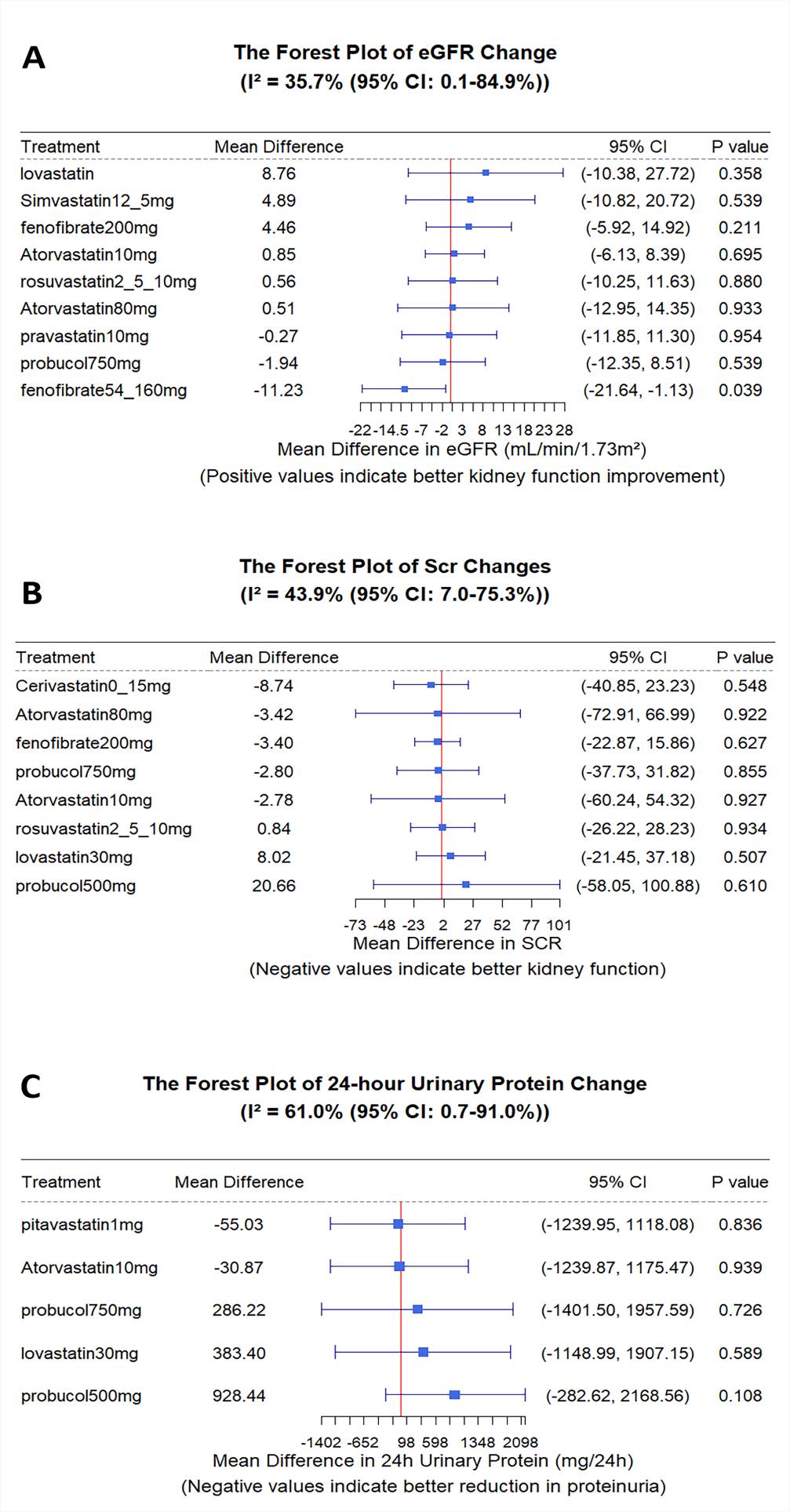
**

**Fig. S2.** The Forest Plot. A: eGFR; B: SCR; C: UPR

**
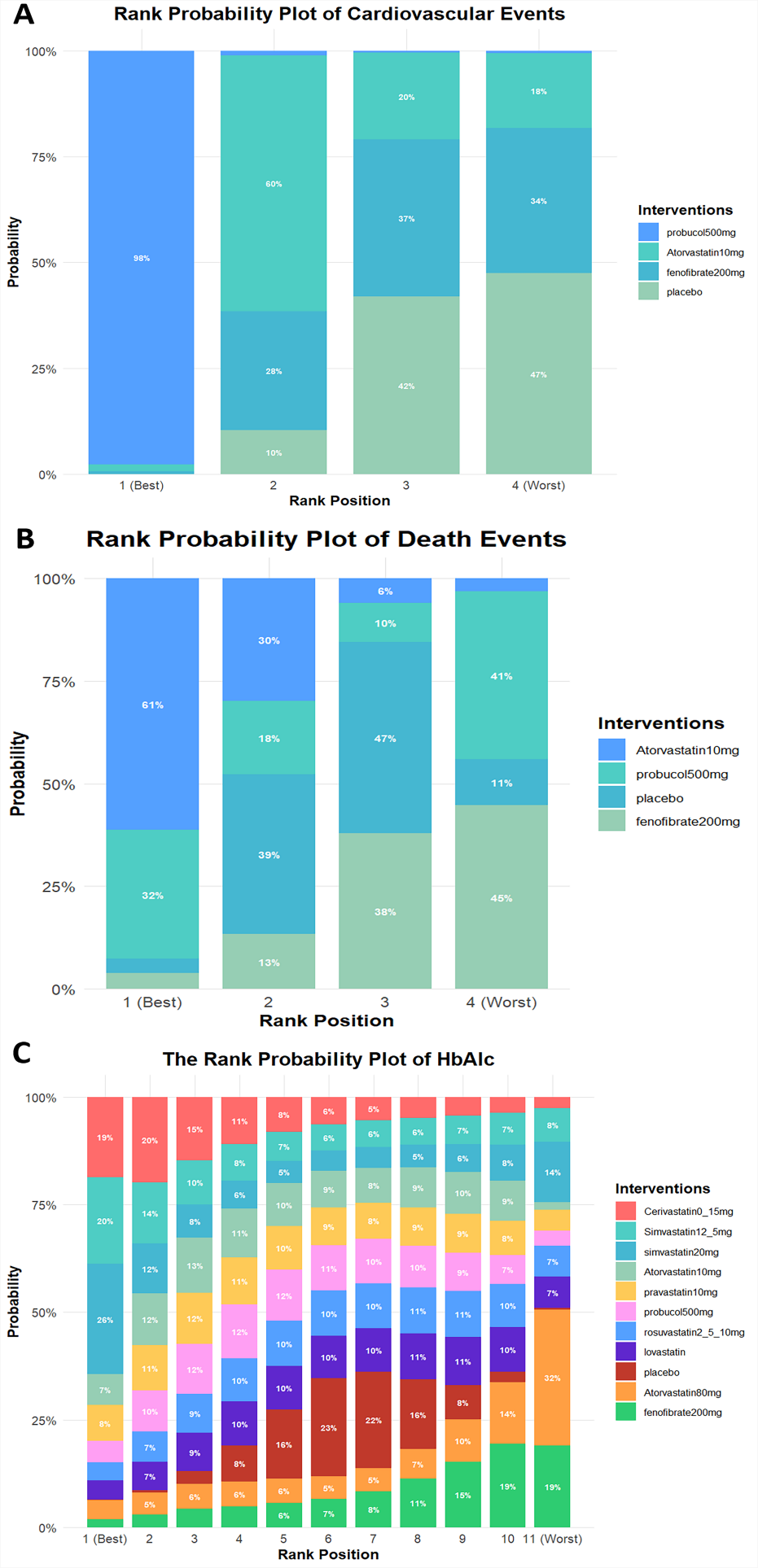
**

**Fig. S3.** The ranking probability plot. A:Cardiovascular Events; B: Death Events; C: HbA1c

**
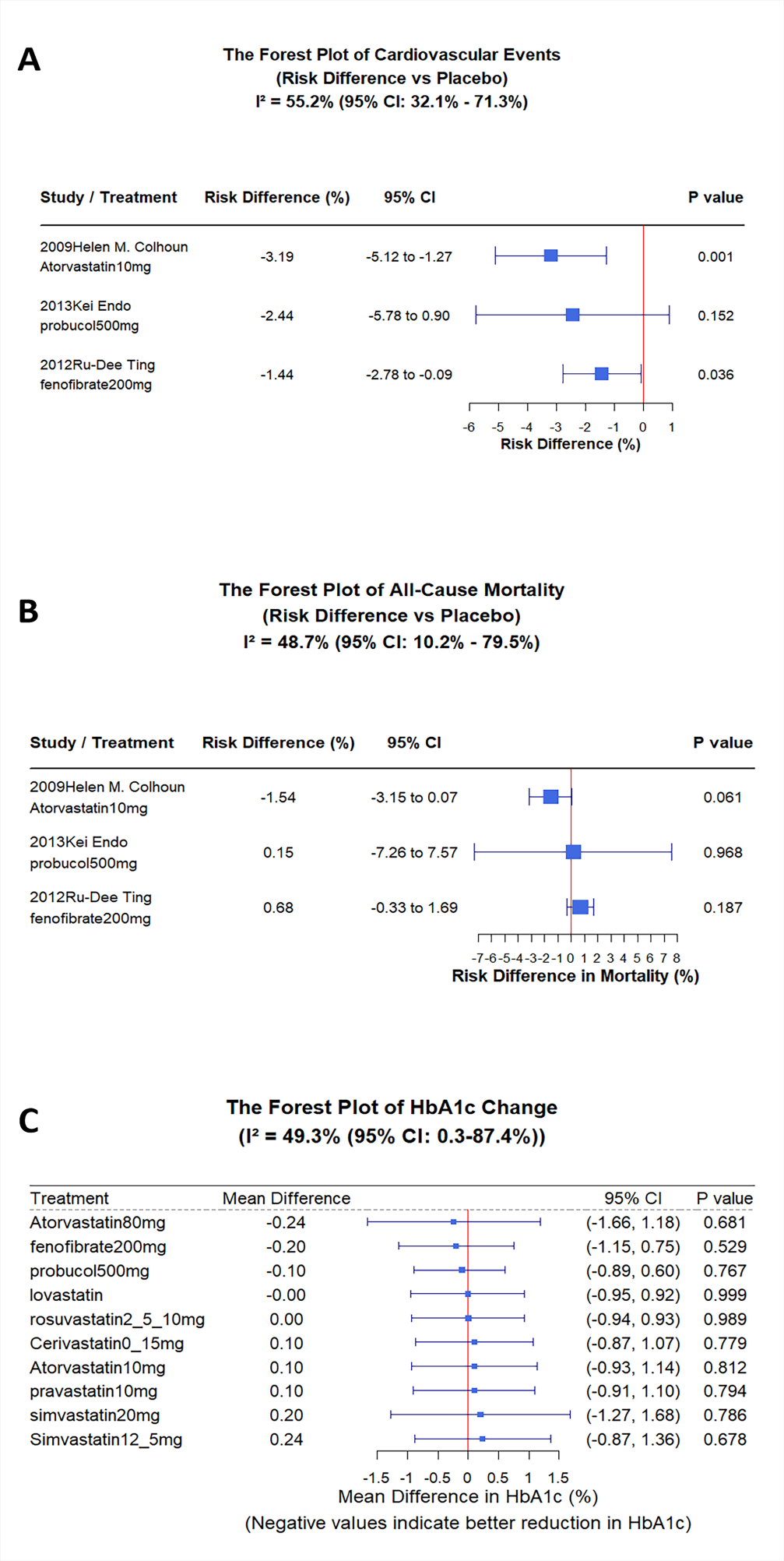
**

**Fig. S4.** The Forest Plot. A:Cardiovascular Events; B: Death Events; C: HbA1c

**
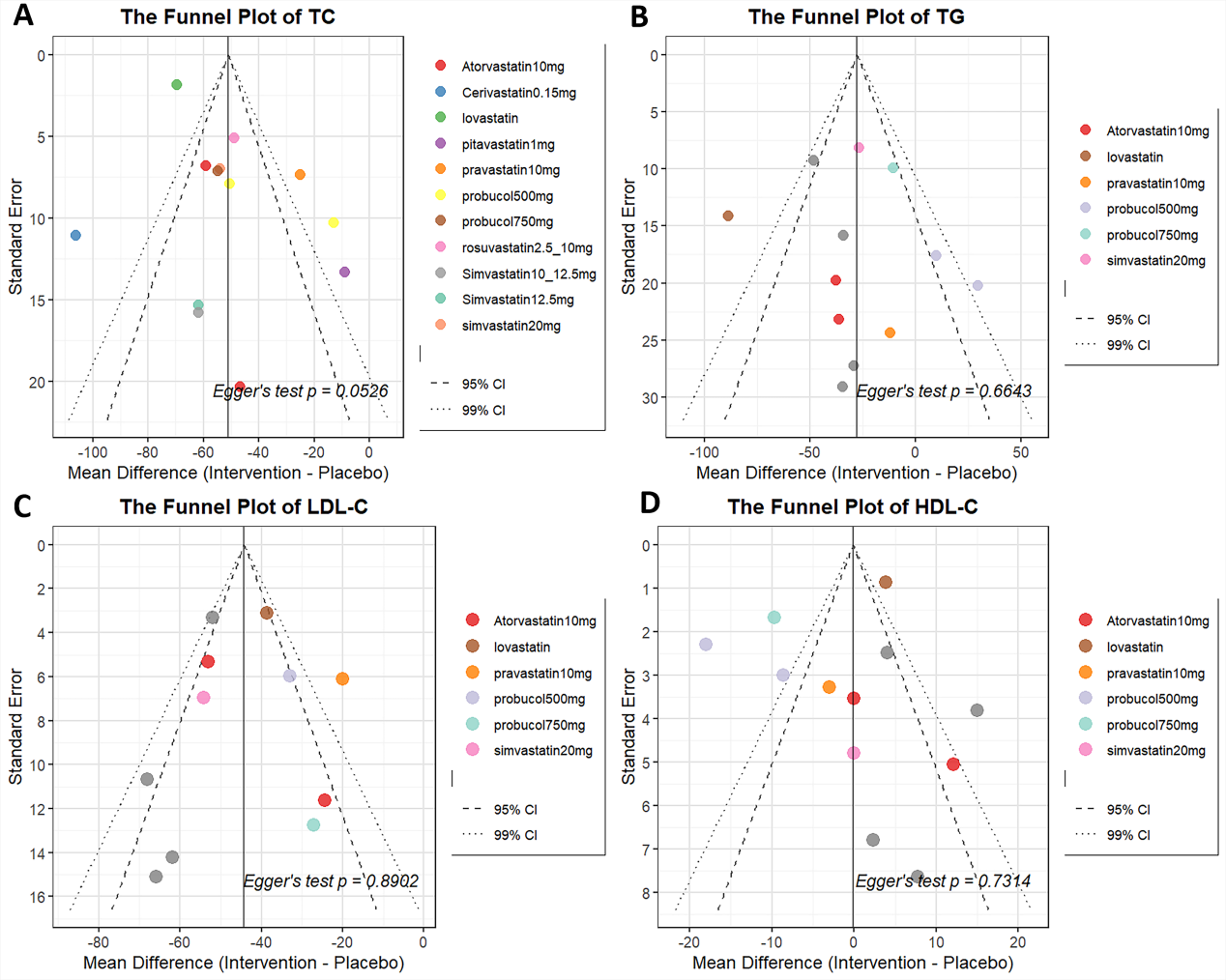
**

**Fig. S5.** The Funnel Plot. A: TC; B: TG; C: LDL-C; D: HDL-C


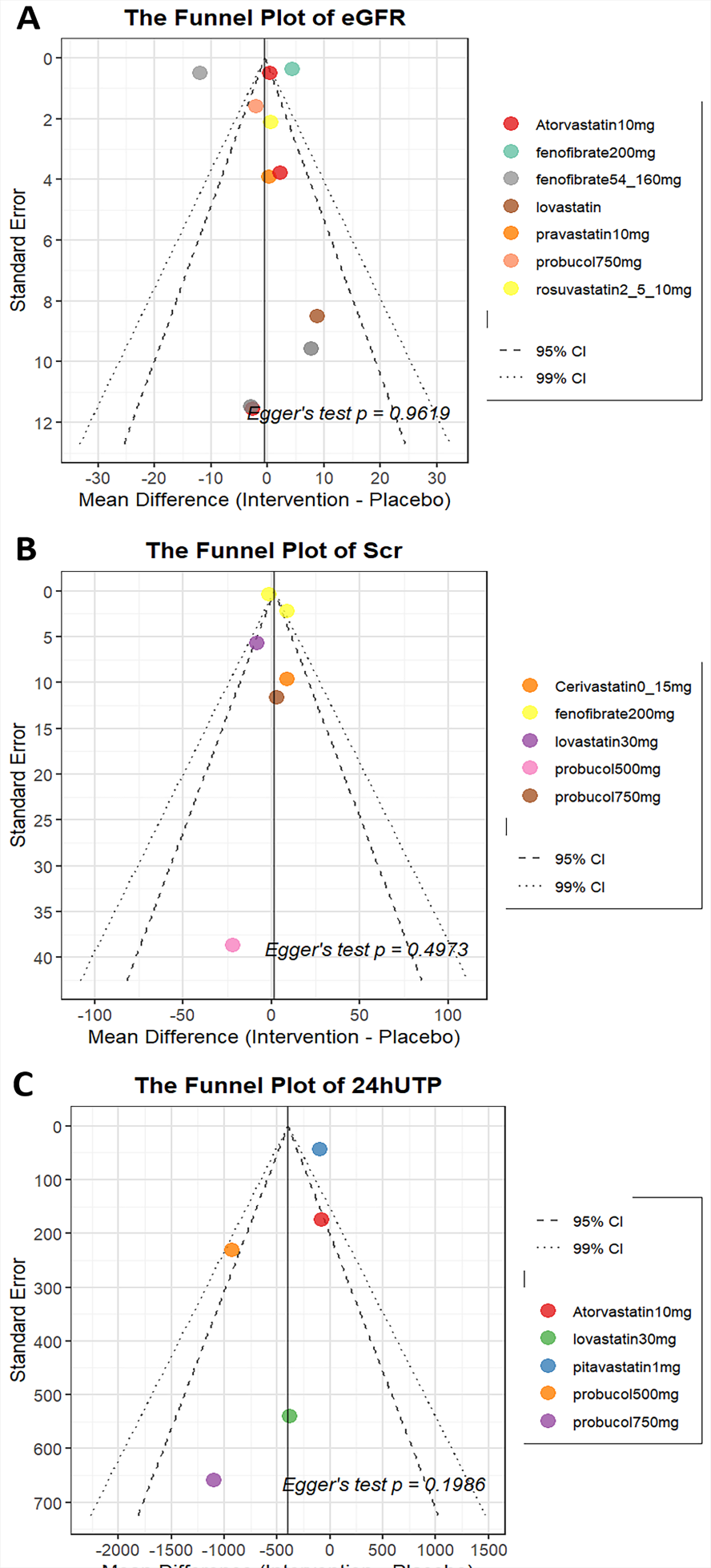


**Fig. S6.** The Funnel Plot. A: eGFR; B: SCR; C: UPR


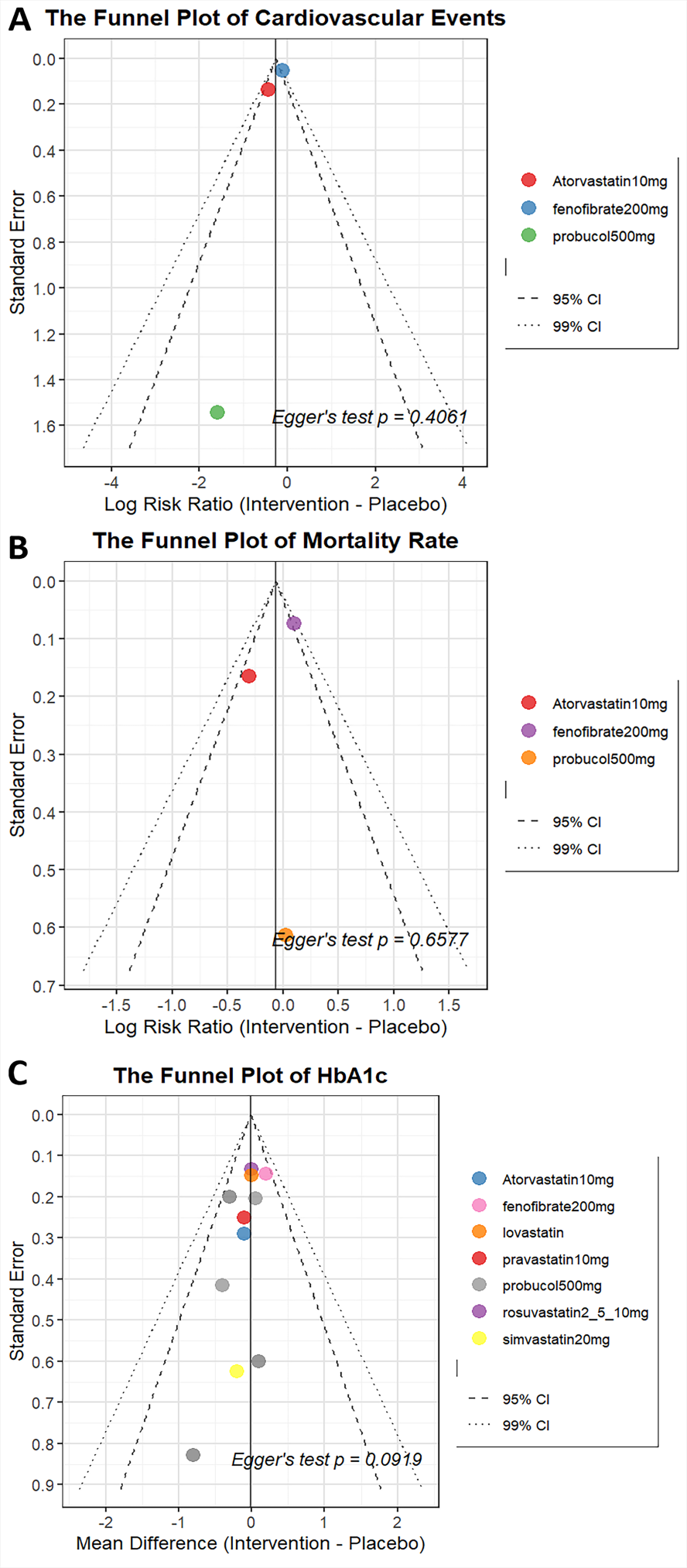


**Fig. S7.** The Funnel Plot. A: HbA1C; B: Cardiovascular Events; C: Death Events

**
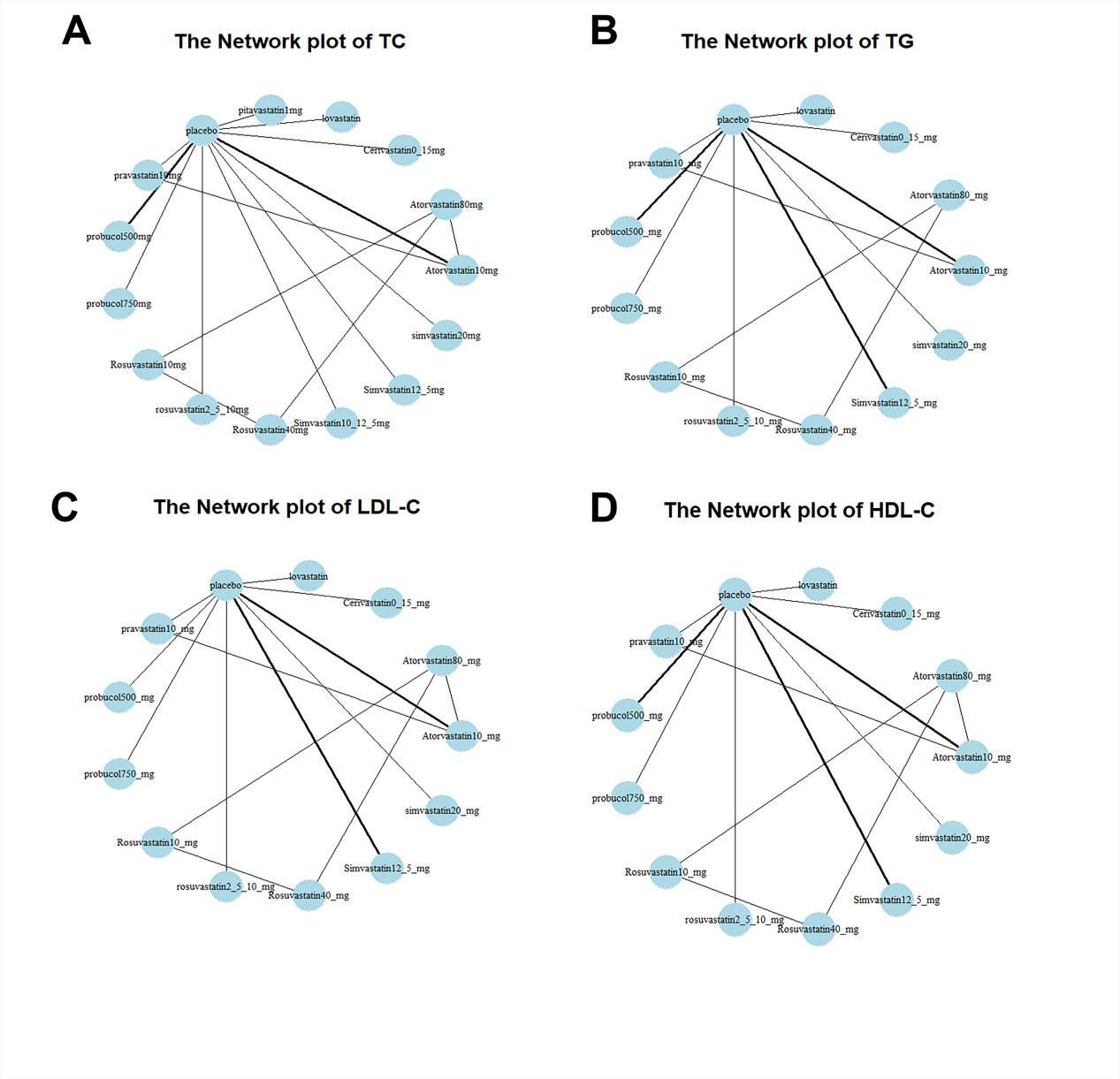
Fig. S8.** The Network Plot A: TC; B: TG; C: LDL-C; D: HDL-C

**
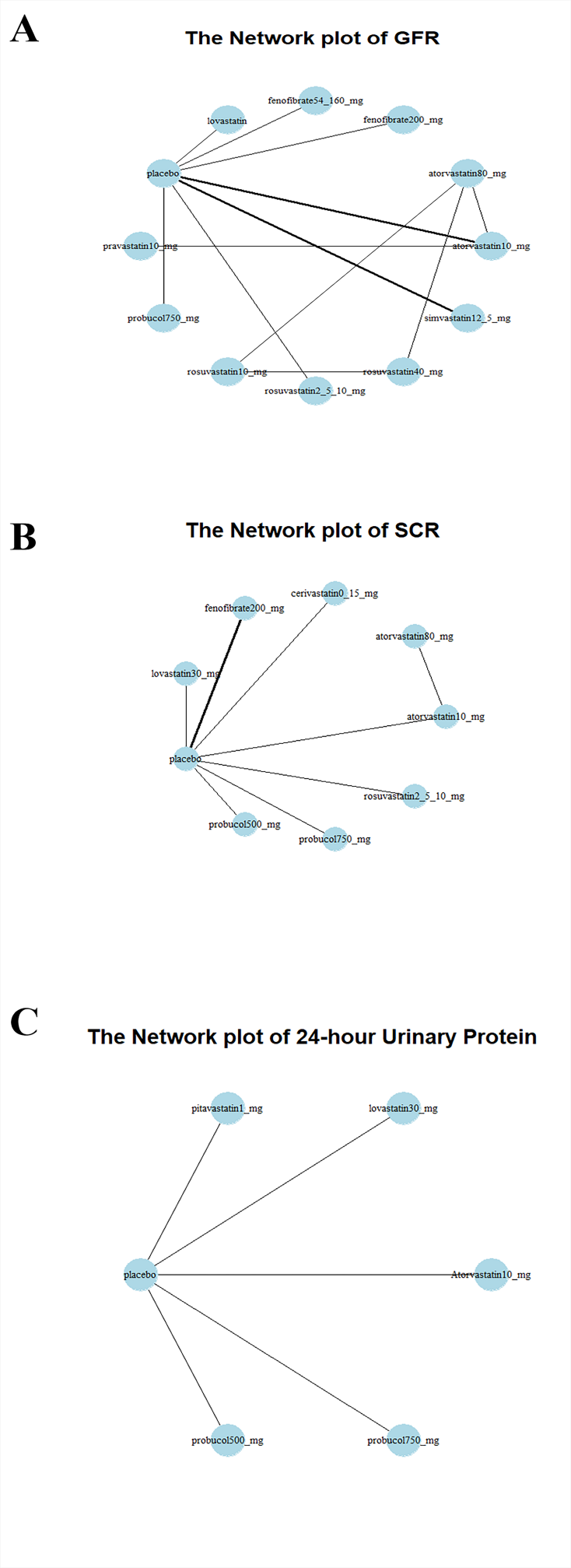
**

**Fig. S9.** The Network Plot. A: eGFR; B: SCR; C: UPR

**
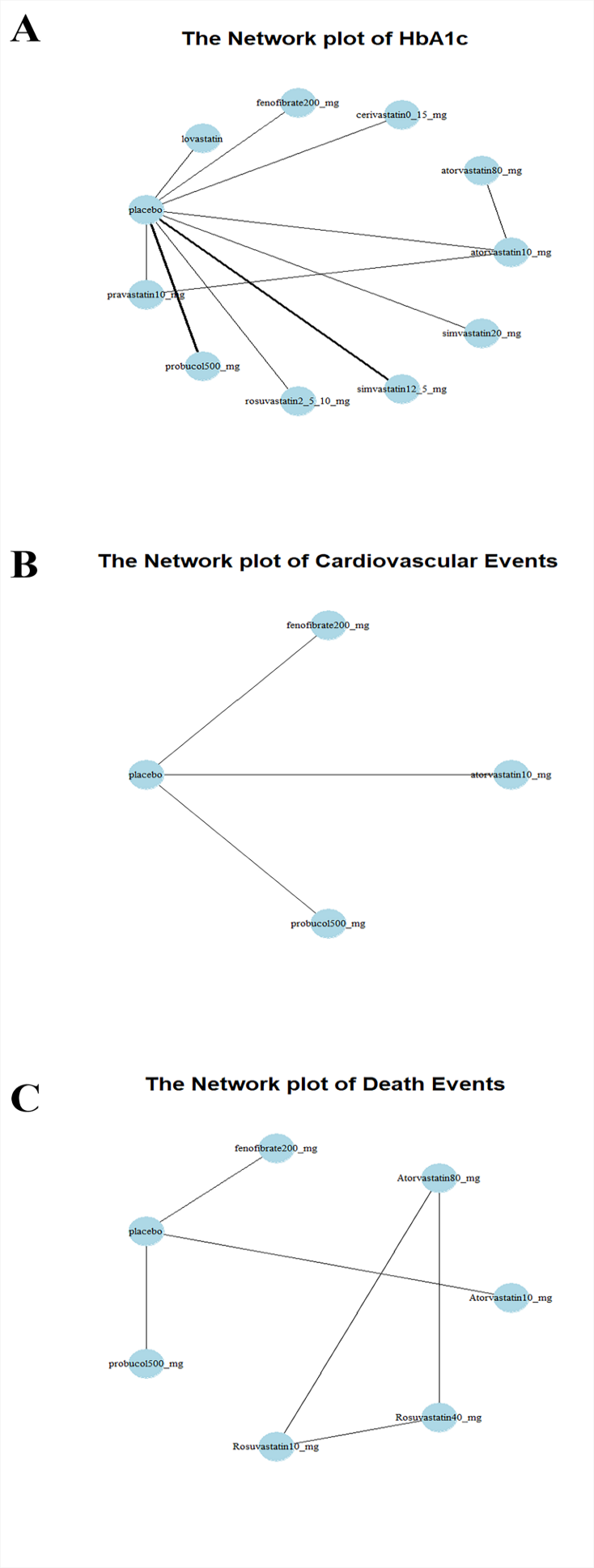
**

**Fig. S10.** The Network Plot. A: HbA1C; B: Cardiovascular Events; C: Death Events

**
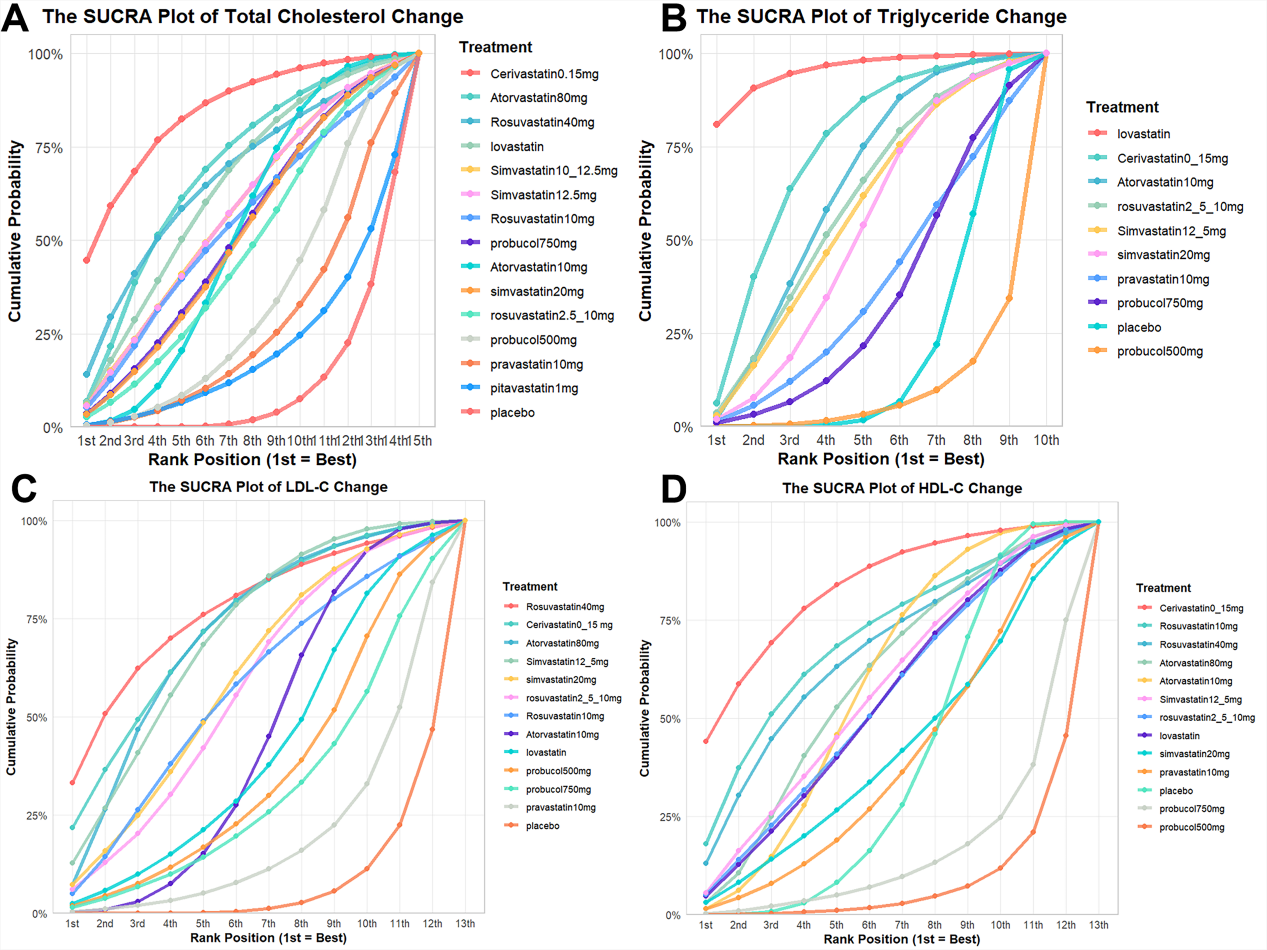
Fig. S11.** The SUCRA Plot. A: TC; B: TG; C: LDL-C; D: HDL-C

**
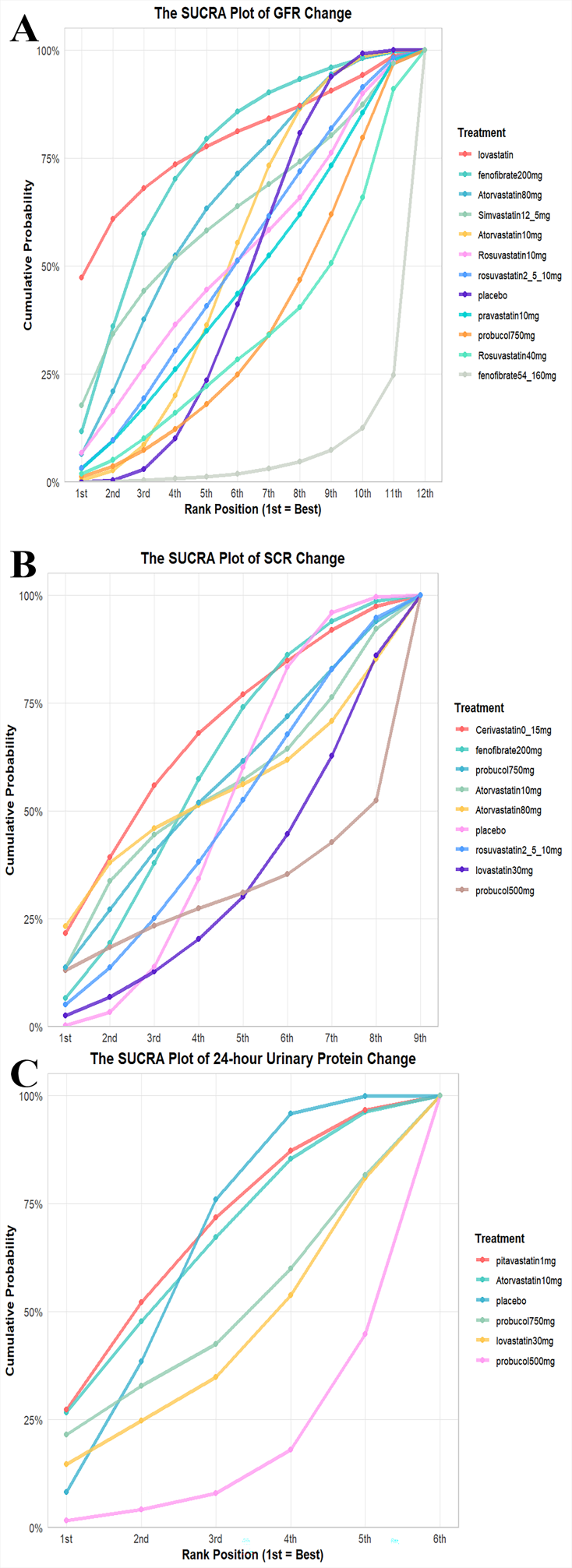
**

**Fig. S12.** The SUCRA Plot A: eGFR; B: SCR; C: UPR

**
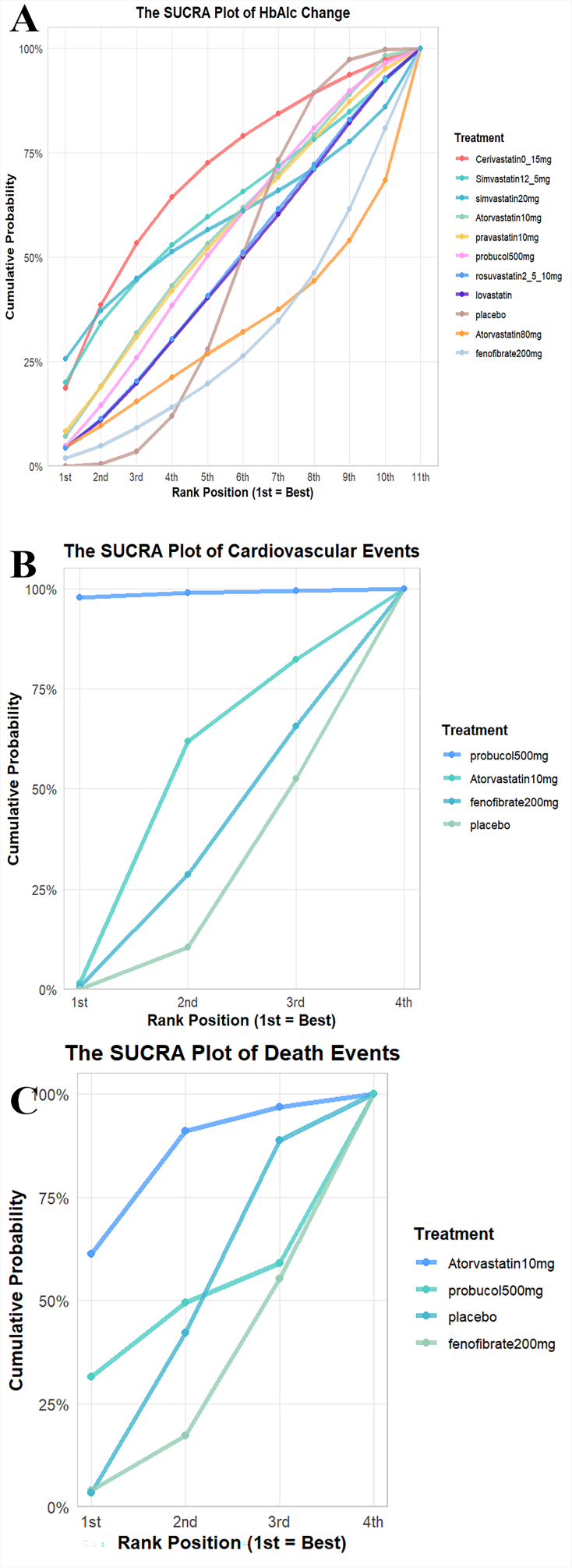
**

**Fig. S13.** The SUCRA Plot A: HbA1c; B: Cardiovascular Events; C: Death Events


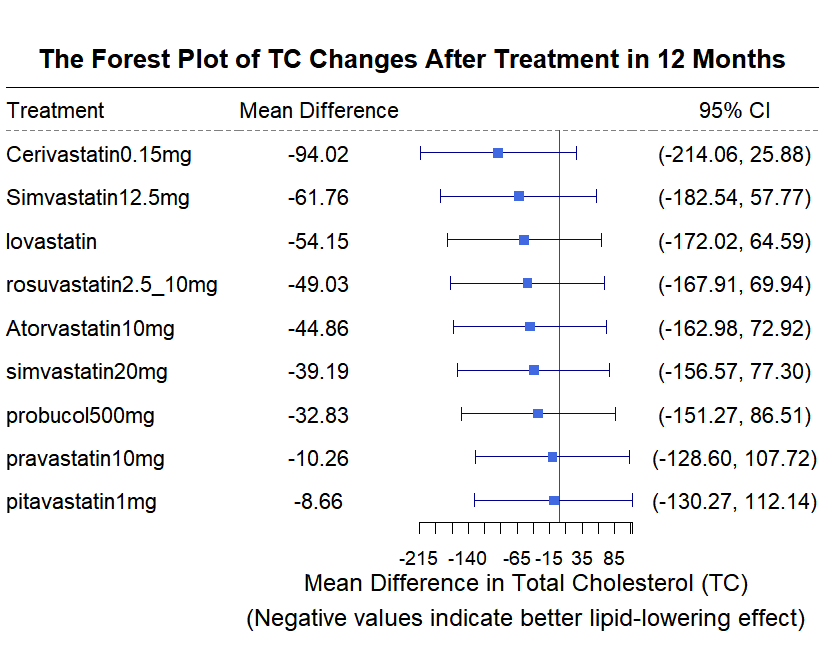


**Fig. S14.** The Forest Plot of TC Changes After Treatment in 12 Months


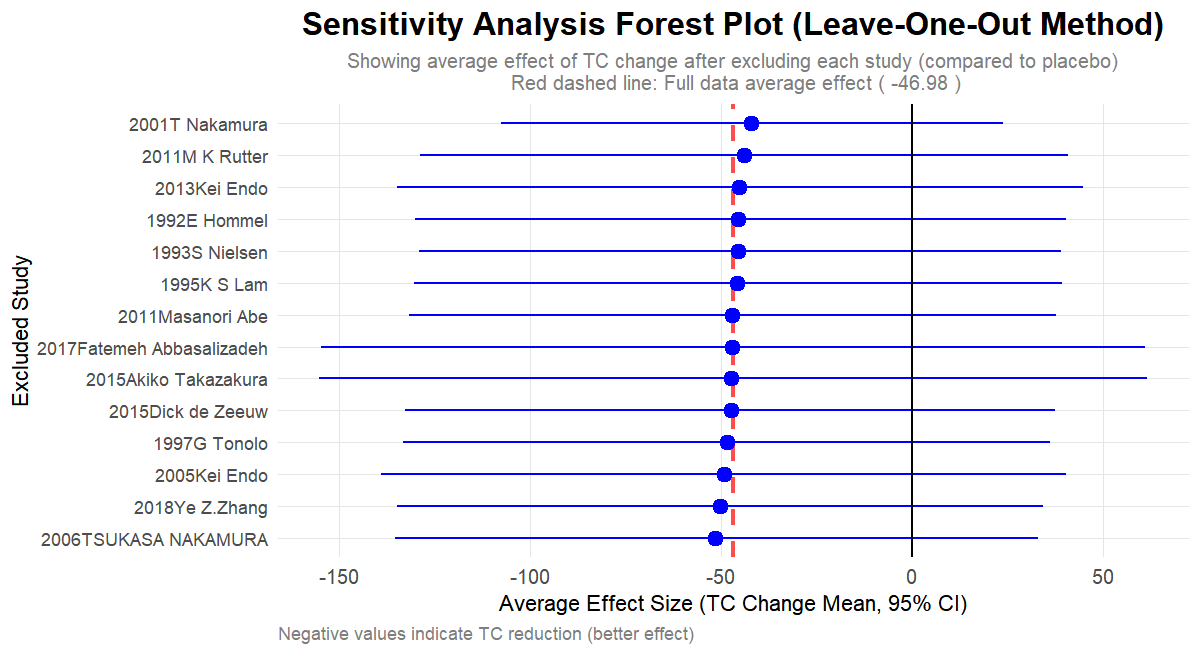


**Fig. S15.** The Sensitivity Analysis Forest Plot (Leave-One-Out Method)
